# Supplementary material for: Boundaries in ground beetle (Coleoptera: Carabidae) and environmental variables at the edges of forest patches with residential developments
Source: PeerJ. 2018 Jan 8;6:e4226. doi: 10.7717/peerj.4226 (PMC5764035; doi:10.7717/peerj.4226)
Supplement: Supplemental Information 1 — Habitat associations and abundances (total numbers of individuals per trap collection) of ground beetle species found at study sites. [file peerj-06-4226-s001.docx]

**Table S1.** Habitat associations and abundances (total numbers of individuals per trap collection) of ground beetle species found at study sites.

| Species | Habitat association | Rural site | Suburban site | Urban site | All sites |
| --- | --- | --- | --- | --- | --- |
| *Agonum punctiforme* | Generalist | 17.87 | 33.73 | 14.77 | 66.37 |
| *Amara aenea* | Open | 14.95 | 8.72 | 8.76 | 32.42 |
| *Amara basillaris* | Unknown | 0.00 | 0.73 | 2.59 | 3.32 |
| *Amara chalcea* | Open | 0.10 | 0.00 | 0.20 | 0.30 |
| *Amara familiaris* | Generalist | 0.31 | 1.26 | 0.71 | 2.28 |
| *Amara impuncticollis* | Generalist | 0.51 | 4.54 | 0.10 | 5.15 |
| *Amphasia interstitialis* | Forest | 0.00 | 0.49 | 0.11 | 0.60 |
| *Anisodactylus dulcicollis* | Generalist | 11.36 | 3.23 | 2.52 | 17.11 |
| *Anisodactylus furvus* | Open | 0.21 | 0.22 | 0.87 | 1.30 |
| *Anisodactylus haplomus* | Forest | 0.00 | 0.09 | 0.11 | 0.20 |
| *Anisodactylus harpaloides* | Open | 0.11 | 0.09 | 0.10 | 0.30 |
| *Anisodactylus nigerrimus* | Generalist | 0.00 | 0.19 | 0.00 | 0.19 |
| *Anisodactylus opaculus* | Generalist | 1.13 | 0.18 | 0.24 | 1.55 |
| *Anisodactylus ovularis* | Generalist | 0.00 | 0.22 | 0.11 | 0.33 |
| *Anisodactylus rusticus* | Generalist | 0.91 | 2.09 | 1.54 | 4.55 |
| *Apenes sinuatus* | Forest | 0.00 | 0.00 | 0.10 | 0.10 |
| *Bembidion versicolor* | Generalist | 0.00 | 0.33 | 0.00 | 0.33 |
| *Calathus opaculus* | Generalist | 0.10 | 0.00 | 0.43 | 0.53 |
| *Chlaenius amoenus* | Forest | 0.00 | 0.55 | 0.00 | 0.55 |
| *Chlaenius emarginatus* | Generalist | 0.30 | 0.61 | 0.00 | 0.91 |
| *Chlaenius prasinus* | Wetland | 0.11 | 9.03 | 0.00 | 9.14 |
| *Chlaenius tomentosus tomentosus* | Generalist | 0.20 | 0.00 | 0.11 | 0.31 |
| *Chlaenius tricolor tricolor* | Generalist | 0.20 | 7.19 | 3.64 | 11.03 |
| *Cyclotrachelus sigillatus* | Generalist | 3.33 | 0.13 | 20.26 | 23.72 |
| *Dicaelus dilatatus dilatatus* | Generalist | 0.00 | 1.25 | 0.00 | 1.25 |
| *Galerita bicolor* | Generalist | 0.51 | 0.32 | 0.00 | 0.83 |
| *Galerita janus* | Generalist | 3.40 | 1.03 | 0.00 | 4.43 |
| *Harpalus erythropus* | Generalist | 0.00 | 0.20 | 0.00 | 0.20 |
| *Harpalus herbivagus* | Generalist | 1.30 | 0.19 | 0.00 | 1.49 |
| *Harpalus longicollis* | Generalist | 0.00 | 0.14 | 1.66 | 1.80 |
| *Harpalus pensylvanicus* | Generalist | 2.48 | 5.10 | 7.56 | 15.15 |
| *Harpalus protractus* | Open | 0.00 | 0.00 | 0.32 | 0.32 |
| *Lebia vittata* | Open | 0.10 | 0.09 | 0.00 | 0.19 |
| *Loxandrus uniformis* | Unknown | 0.00 | 0.21 | 0.00 | 0.21 |
| *Olisthopus parmatus* | Generalist | 0.00 | 0.10 | 0.00 | 0.10 |
| *Oodes fluvialis* | Wetland | 0.00 | 3.91 | 0.00 | 3.91 |
| *Pasimachus punctulatus* | Generalist | 0.42 | 0.00 | 0.00 | 0.42 |
| *Platynus decentis* | Forest | 0.00 | 0.00 | 0.41 | 0.41 |
| *Poecilus lucublandus lucublandus* | Generalist | 1.06 | 7.38 | 0.58 | 9.02 |
| *Pterostichus sculptus* | Generalist | 24.03 | 19.47 | 14.08 | 57.58 |
| *Scaphinotus unicolor unicolor* | Forest | 0.00 | 0.11 | 0.00 | 0.11 |
| *Scarites quadriceps* | Generalist | 11.27 | 5.30 | 9.46 | 26.03 |
| *Scarites subterraneus* | Generalist | 0.10 | 5.21 | 0.00 | 5.31 |
| *Selenophorus opalinus* | Generalist | 0.10 | 0.09 | 0.00 | 0.19 |
| *Sphaeroderus stenostomus lecontei* | Generalist | 15.91 | 0.00 | 9.98 | 25.89 |
| *Stenolophus ochropezus* | Generalist | 0.00 | 0.49 | 0.14 | 0.63 |
| *Stenolophus rotundatus* | Open | 0.10 | 0.64 | 0.22 | 0.96 |
| *Trichotichnus autumnalis* | Forest | 0.00 | 0.00 | 0.24 | 0.24 |
| *Trichotichnus dichrous* | Forest | 0.20 | 0.00 | 0.00 | 0.20 |
| *Trichotichnus fulgens* | Generalist | 2.51 | 7.15 | 1.06 | 10.72 |
