# Supplementary material for: Boundaries in ground beetle (Coleoptera: Carabidae) and environmental variables at the edges of forest patches with residential developments
Source: PeerJ. 2018 Jan 8;6:e4226. doi: 10.7717/peerj.4226 (PMC5764035; doi:10.7717/peerj.4226)
Supplement: Supplemental Information 2 — Boundary statistics calculated for environmental and ground beetle variables. Beetle community matrices were analyzed using raw abundances and species abundances weighted by the inverse of the species’ proportion of total abundance at the site. NS: total number of boundaries and singletons; N1: number of singletons; Lmean: mean length of boundaries and singletons (number of candidate boundary elements); Lmax: maximum length; Dmean: mean diameter of boundaries (minimum number of links between the farthest pair of candidate boundary elements); Dmax: maximum diameter; D∕L: mean diameter-to-length ratio. ∗p < 0.10. [file peerj-06-4226-s002.docx]

**Table S2.** Boundary statistics calculated for environmental and ground beetle variables measured at the rural site at the small spatial scale. Beetle community matrices were analyzed using raw abundances and species abundances weighted by the inverse of the species' proportion of total abundance at the site. *N_S_*: total number of boundaries and singletons; *N_1_*: number of singletons; *L_mean_*: mean length of boundaries and singletons (number of candidate boundary elements); *L_max_*: maximum length; *D_mean_*: mean diameter of boundaries (minimum number of links between the farthest pair of candidate boundary elements); *D_max_*: maximum diameter; *D/L*: mean diameter-to-length ratio. **p* < 0.10.

| Variable | *N_s_* | *N_1_* | *L_mean_* | *L_max_* | *D_mean_* | *D_max_* | *D/L* |
| --- | --- | --- | --- | --- | --- | --- | --- |
| Temperature | 9 | 5 | 2.444 | 6 | 2.444^*^ | 6 | 1.000 |
| Humidity | 12 | 7 | 1.833 | 5 | 1.833 | 5 | 1.000 |
| Microrelief | 13 | 6 | 1.923 | 4 | 1.923 | 4 | 1.000 |
| Slope | 13 | 7 | 1.923 | 4 | 1.923 | 4 | 1.000 |
| Leaf litter depth | 12 | 5 | 2.083 | 7^*^ | 2.083 | 7^*^ | 1.000 |
| Canopy cover | 5^*^ | 1^*^ | 5.000^*^ | 11^*^ | 5.000^*^ | 11^*^ | 1.000 |
| Forb cover | 9^*^ | 3 | 2.778^*^ | 7^*^ | 2.778^*^ | 7^*^ | 1.000 |
| Grass cover | 9^*^ | 2^*^ | 2.778^*^ | 6^*^ | 2.778^*^ | 6^*^ | 1.000 |
| Shrub cover | 16 | 8 | 1.563 | 3 | 1.563 | 3 | 1.000 |
| Vine cover | 8^*^ | 4 | 3.125^*^ | 11^*^ | 3.125^*^ | 11^*^ | 1.000 |
| Bare ground cover | 11 | 4 | 2.273 | 4 | 2.273 | 4 | 1.000 |
| Rock cover | 7^*^ | 1 | 3.571^*^ | 7 | 3.286^*^ | 7 | 0.959^*^ |
| Impervious surface cover | 9 | 2 | 2.778 | 9 | 2.444 | 6 | 0.963 |
| Coarse woody debris cover | 12 | 5 | 2.083 | 5 | 1.917 | 3 | 0.967^*^ |
| All environmental variables | 13 | 6 | 1.923 | 4 | 1.846 | 4 | 0.981^*^ |
| Total abundance | 11 | 5 | 2.273 | 6 | 2.273 | 6 | 1.000 |
| Total species richness | 13 | 4 | 1.923 | 3 | 1.923 | 3 | 1.000 |
| Total species evenness | 11 | 4 | 1.909^*^ | 4 | 1.909^*^ | 4 | 1.000 |
| Open-habitat species abundance | 6^*^ | 0^*^ | 4.167^*^ | 6 | 4.167^*^ | 6 | 1.000 |
| Open-habitat species richness | 11 | 4 | 2.273 | 5 | 2.273 | 5 | 1.000 |
| Generalist species abundance | 10 | 5 | 2.500 | 7 | 2.500 | 7 | 1.000 |
| Generalist species richness | 11 | 3 | 2.273 | 5 | 2.273 | 5 | 1.000 |
| Generalist species evenness | 12 | 6 | 1.750 | 4 | 1.750 | 4 | 1.000 |
| All species | 12^*^ | 6^*^ | 2.083^*^ | 4 | 2.083^*^ | 4 | 1.000 |
| All species weighted | 18 | 13 | 1.389 | 4 | 1.389 | 4 | 1.000 |
| Open-habitat species | 13 | 7 | 1.923 | 5 | 1.923 | 5 | 1.000 |
| Open-habitat species weighted | 15 | 9 | 1.667 | 4 | 1.667 | 4 | 1.000 |
| Generalist species | 17 | 11 | 1.471 | 3 | 1.471 | 3 | 1.000 |
| Generalist species weighted | 14 | 10 | 1.786 | 7 | 1.786 | 7 | 1.000 |
| *Agonum punctiforme* | 8 | 3 | 3.125 | 8 | 3.125 | 8 | 1.000 |
| *Amara aenea* | 6^*^ | 0^*^ | 4.167^*^ | 6 | 4.167^*^ | 6 | 1.000 |
| *Anisodactylus dulcicollis* | 12 | 5 | 2.083 | 6 | 2.083 | 6 | 1.000 |
| *Anisodactylus opaculus* | 9 | 3 | 2.778 | 5 | 2.778 | 5 | 1.000 |
| *Anisodactylus rusticus* | 11 | 2 | 2.273 | 5 | 2.273 | 5 | 1.000 |
| *Cyclotrachelus sigillatus* | 11 | 5 | 2.273 | 5 | 2.273 | 5 | 1.000 |
| *Galerita janus* | 10 | 3 | 2.500 | 5 | 2.500 | 5 | 1.000 |
| *Harpalus pensylvanicus* | 7 | 0^*^ | 3.571 | 7 | 3.286 | 5 | 0.959 |
| *Poecilus lucublandus lucublandus* | 8 | 3 | 3.125 | 10 | 3.000 | 9 | 0.988 |
| *Pterostichus sculptus* | 10 | 4 | 2.500 | 7 | 2.500 | 7 | 1.000 |
| *Scarites subterraneus* | 9 | 2 | 2.778 | 6 | 2.778 | 6 | 1.000 |
| *Sphaeroderus stenostomus lecontei* | 14^*^ | 6 | 1.786^*^ | 4 | 1.786^*^ | 4 | 1.000 |
| *Trichotichnus fulgens* | 11 | 3 | 2.273 | 6 | 2.091 | 4^*^ | 0.970 |

**Table S3.** Boundary statistics calculated for environmental and ground beetle variables measured at the rural site at the large spatial scale. Beetle community matrices were analyzed using raw abundances and species abundances weighted by the inverse of the species' proportion of total abundance at the site. *N_S_*: total number of boundaries and singletons; *N_1_*: number of singletons; *L_mean_*: mean length of boundaries and singletons (number of candidate boundary elements); *L_max_*: maximum length; *D_mean_*: mean diameter of boundaries (minimum number of links between the farthest pair of candidate boundary elements); *D_max_*: maximum diameter; *D/L*: mean diameter-to-length ratio. **p* < 0.10.

| Variable | *N_s_* | *N_1_* | *L_mean_* | *L_max_* | *D_mean_* | *D_max_* | *D/L* |
| --- | --- | --- | --- | --- | --- | --- | --- |
| Temperature | 2^*^ | 0 | 3.500^*^ | 4 | 3.500^*^ | 4 | 1.000 |
| Humidity | 3 | 2 | 2.333 | 5^*^ | 2.333 | 5^*^ | 1.000 |
| Microrelief | 5 | 3 | 1.400 | 2 | 1.400 | 2 | 1.000 |
| Slope | 3 | 2 | 2.333 | 5^*^ | 2.333 | 5^*^ | 1.000 |
| Leaf litter depth | 5 | 4^*^ | 1.400 | 3 | 1.400 | 3 | 1.000 |
| Canopy cover | 3 | 0 | 2.333 | 3 | 2.333 | 3 | 1.000 |
| Forb cover | 2 | 1 | 3.500 | 6^*^ | 3.500 | 6^*^ | 1.000 |
| Grass cover | 3 | 1 | 2.333 | 4 | 2.333 | 4 | 1.000 |
| Creeping forb cover | 3 | 1 | 2.333 | 3 | 2.333 | 3 | 1.000 |
| Shrub cover | 4 | 3 | 1.750 | 4 | 1.750 | 4 | 1.000 |
| Vine cover | 4 | 3 | 1.750 | 4 | 1.750 | 4 | 1.000 |
| Bare ground cover | 6^*^ | 5^*^ | 1.167^*^ | 2 | 1.167^*^ | 2 | 1.000 |
| Rock cover | 2 | 0 | 3.500 | 4 | 3.500 | 4 | 1.000 |
| Impervious surface cover | 4 | 2 | 1.750 | 3 | 1.750 | 3 | 1.000 |
| Coarse woody debris cover | 4 | 2 | 1.750 | 3 | 1.750 | 3 | 1.000 |
| Tree cover | 3 | 0 | 2.333 | 3 | 2.333 | 3 | 1.000 |
| Moss cover | 4 | 1 | 1.750 | 2 | 1.750 | 2 | 1.000 |
| All environmental variables | 4 | 3 | 1.750 | 4 | 1.750 | 4 | 1.000 |
| Total abundance | 4 | 2 | 1.750 | 3 | 1.750 | 3 | 1.000 |
| Total species richness | 3 | 1 | 2.333 | 4 | 2.333 | 4 | 1.000 |
| Total species evenness | 2^*^ | 1 | 3.500^*^ | 6^*^ | 3.500^*^ | 6^*^ | 1.000 |
| Open-habitat species abundance | 3 | 1 | 2.333 | 3 | 2.333 | 3 | 1.000 |
| Open-habitat species richness | 5 | 3 | 1.400 | 2 | 1.400 | 2 | 1.000 |
| Generalist species abundance | 3 | 2 | 2.333 | 5 | 2.333 | 5^*^ | 1.000 |
| Generalist species richness | 5 | 4^*^ | 1.400 | 3 | 1.400 | 3 | 1.000 |
| Generalist species evenness | 2^*^ | 1 | 3.500^*^ | 6^*^ | 3.500^*^ | 6^*^ | 1.000 |
| All species | 3 | 1 | 2.333 | 4 | 2.000 | 3 | 0.917^*^ |
| All species weighted | 3 | 1 | 2.333 | 4 | 2.000 | 3 | 0.917^*^ |
| Open-habitat species | 3 | 1 | 2.333 | 3 | 2.333 | 3 | 1.000 |
| Open-habitat species weighted | 4 | 1 | 1.750 | 2 | 1.750 | 2 | 1.000 |
| Generalist species | 2 | 0 | 3.500 | 5 | 3.000 | 4 | 0.900^*^ |
| Generalist species weighted | 3 | 1 | 2.333 | 3 | 2.333 | 3 | 1.000 |
| *Agonum punctiforme* | 4 | 3 | 1.750 | 4 | 1.750 | 4 | 1.000 |
| *Amara aenea* | 3 | 0 | 2.333 | 3 | 2.333 | 3 | 1.000 |
| *Amara familiaris* | 4 | 2 | 1.750 | 3 | 1.750 | 3 | 1.000 |
| *Anisodactylus dulcicollis* | 2^*^ | 1 | 3.500^*^ | 6^*^ | 3.500^*^ | 6^*^ | 1.000 |
| *Anisodactylus opaculus* | 5^*^ | 4^*^ | 1.400^*^ | 3 | 1.400^*^ | 3 | 1.000 |
| *Anisodactylus rusticus* | 2 | 1 | 3.500 | 6 | 3.500 | 6^*^ | 1.000 |
| *Chlaenius emarginatus* | 3 | 1 | 2.333 | 4 | 2.333 | 4 | 1.000 |
| *Chlaenius prasinus* | 1^*^ | 0^*^ | 7.000 | 7^*^ | 7.000^*^ | 7^*^ | 1.000^*^ |
| *Chlaenius tomentosus tomentosus* | 2 | 0 | 3.500 | 4 | 3.500 | 4 | 1.000 |
| *Chlaenius tricolor tricolor* | 3 | 0 | 2.333 | 3 | 2.333 | 3 | 1.000 |
| *Cyclotrachelus sigillatus* | 3 | 1 | 2.333 | 3 | 2.333 | 3 | 1.000 |
| *Galerita bicolor* | 4 | 2 | 1.750 | 3 | 1.750 | 3 | 1.000 |
| *Galerita janus* | 4 | 3^*^ | 1.750 | 4 | 1.750 | 4 | 1.000 |
| *Harpalus herbivagus* | 3 | 1 | 2.333 | 3 | 2.333 | 3 | 1.000 |
| *Harpalus pensylvanicus* | 3 | 1 | 2.333 | 3 | 2.333 | 3 | 1.000 |
| *Lebia vittata* | 1^*^ | 0^*^ | 7.000 | 7^*^ | 5.000 | 5^*^ | 0.714^*^ |
| *Pasimachus punctulatus* | 2 | 0 | 3.500 | 5 | 3.500 | 5 | 1.000 |
| *Poecilus lucublandus lucublandus* | 3 | 0 | 2.333 | 3 | 2.333 | 3 | 1.000 |
| *Pterostichus sculptus* | 3 | 1 | 2.333 | 3 | 2.333 | 3 | 1.000 |
| *Scarites subterraneus* | 4 | 2 | 1.750 | 3 | 1.750 | 3 | 1.000 |
| *Sphaeroderus stenostomus lecontei* | 4 | 2 | 1.750 | 3 | 1.750 | 3 | 1.000 |
| *Trichotichnus fulgens* | 2 | 0 | 3.500 | 5 | 3.500 | 5 | 1.000 |

**Table S4.** Boundary statistics calculated for environmental and ground beetle variables measured at the suburban site at the small spatial scale. Beetle community matrices were analyzed using raw abundances and species abundances weighted by the inverse of the species' proportion of total abundance at the site. *N_S_*: total number of boundaries and singletons; *N_1_*: number of singletons; *L_mean_*: mean length of boundaries and singletons (number of candidate boundary elements); *L_max_*: maximum length; *D_mean_*: mean diameter of boundaries (minimum number of links between the farthest pair of candidate boundary elements); *D_max_*: maximum diameter; *D/L*: mean diameter-to-length ratio. **p* < 0.10.

| Variable | *N_s_* | *N_1_* | *L_mean_* | *L_max_* | *D_mean_* | *D_max_* | *D/L* |
| --- | --- | --- | --- | --- | --- | --- | --- |
| Temperature | 12 | 6 | 1.917 | 5 | 1.917 | 5 | 1.000 |
| Humidity | 12 | 7 | 1.917 | 5 | 1.917 | 5 | 1.000 |
| Microrelief | 13 | 5 | 1.923 | 4 | 1.923 | 4 | 1.000 |
| Slope | 9^*^ | 4 | 2.778^*^ | 8^*^ | 2.556^*^ | 6 | 0.972^*^ |
| Leaf litter depth | 10^*^ | 4 | 2.500^*^ | 8^*^ | 2.500^*^ | 8^*^ | 1.000 |
| Canopy cover | 13 | 7 | 1.923 | 5 | 1.923 | 5 | 1.000 |
| Forb cover | 9^*^ | 2 | 2.778^*^ | 6 | 2.778^*^ | 6 | 1.000 |
| Grass cover | 12^*^ | 5 | 2.083^*^ | 6^*^ | 2.083^*^ | 6^*^ | 1.000 |
| Creeping forb cover | 9^*^ | 4 | 2.778^*^ | 7^*^ | 2.556^*^ | 6 | 0.968^*^ |
| Shrub cover | 10^*^ | 4 | 2.500^*^ | 5 | 2.500^*^ | 5 | 1.000 |
| Vine cover | 13 | 4 | 1.923 | 3 | 1.923 | 3 | 1.000 |
| Bare ground cover | 12 | 3 | 2.083 | 4 | 2.083 | 4 | 1.000 |
| Impervious surface cover | 9 | 4 | 2.778 | 8 | 2.667 | 7 | 0.986 |
| Coarse woody debris cover | 10 | 3 | 2.500 | 6 | 2.500 | 6 | 1.000 |
| Moss cover | 12^*^ | 7 | 2.083 | 7 | 2.083 | 7 | 1.000 |
| All environmental variables | 13 | 7 | 1.923 | 7^*^ | 1.769 | 5 | 0.978^*^ |
| Total abundance | 14 | 7 | 1.786 | 5 | 1.786 | 5 | 1.000 |
| Total species richness | 12 | 6 | 2.083 | 5 | 2.083 | 5 | 1.000 |
| Total species evenness | 12^*^ | 4 | 1.833 | 4^*^ | 1.833 | 4^*^ | 1.000 |
| Forest species abundance | 13 | 4 | 1.923 | 3 | 1.923 | 3 | 1.000 |
| Forest species richness | 13 | 5 | 1.923 | 4 | 1.923 | 4 | 1.000 |
| Open-habitat species abundance | 8 | 3 | 3.125 | 9 | 3.125 | 9 | 1.000 |
| Open-habitat species richness | 13 | 2 | 1.923 | 3 | 1.923 | 3 | 1.000 |
| Generalist species abundance | 12 | 6 | 2.083 | 6 | 2.083 | 6 | 1.000 |
| Generalist species richness | 13 | 5 | 1.923 | 5 | 1.923 | 5 | 1.000 |
| Generalist species evenness | 11^*^ | 3 | 2.000^*^ | 4^*^ | 2.000^*^ | 4^*^ | 1.000 |
| All species | 17 | 13 | 1.471 | 5 | 1.471 | 5 | 1.000 |
| All species weighted | 20 | 16 | 1.250 | 3 | 1.250 | 3 | 1.000 |
| Forest species | 18 | 14 | 1.389 | 3 | 1.389 | 3 | 1.000 |
| Forest species weighted | 16 | 11 | 1.563 | 4 | 1.563 | 4 | 1.000 |
| Open-habitat species | 14 | 7 | 1.786 | 4 | 1.714 | 4 | 0.982^*^ |
| Open-habitat species weighted | 16 | 11 | 1.563 | 4 | 1.563 | 4 | 1.000 |
| Generalist species | 18 | 15 | 1.389 | 5 | 1.389 | 5 | 1.000 |
| Generalist species weighted | 20 | 15 | 1.250 | 2^*^ | 1.250 | 2^*^ | 1.000 |
| *Agonum punctiforme* | 13 | 5 | 1.923 | 4 | 1.923 | 4 | 1.000 |
| *Amara aenea* | 6 | 1 | 4.167 | 8 | 4.167^*^ | 8 | 1.000 |
| *Amara basillaris* | 9 | 2 | 2.778 | 6 | 2.778 | 6 | 1.000 |
| *Amara familiaris* | 11 | 3 | 2.273 | 7 | 2.273 | 7 | 1.000 |
| *Amara impuncticollis* | 10 | 1 | 2.500 | 7 | 2.300 | 5 | 0.971 |
| *Anisodactylus dulcicollis* | 9 | 2 | 2.778 | 6 | 2.556 | 6 | 0.956^*^ |
| *Anisodactylus rusticus* | 8 | 1 | 3.125 | 6 | 3.125 | 6 | 1.000 |
| *Chlaenius prasinus* | 8 | 1 | 3.125 | 7 | 2.875 | 5 | 0.964 |
| *Chlaenius tricolor tricolor* | 8 | 3 | 3.125 | 9 | 2.875 | 7 | 0.972 |
| *Dicaelus dilatatus dilatatus* | 9 | 1 | 2.778 | 5 | 2.556 | 5 | 0.956^*^ |
| *Galerita janus* | 12 | 5 | 2.083 | 5 | 2.083 | 5 | 1.000 |
| *Harpalus pensylvanicus* | 9 | 3 | 2.778 | 7 | 2.778 | 7 | 1.000 |
| *Oodes fluvialis* | 8 | 4 | 3.125 | 8 | 2.875 | 7 | 0.969 |
| *Poecilus lucublandus lucublandus* | 11 | 7^*^ | 2.273 | 7 | 2.273 | 7 | 1.000 |
| *Pterostichus sculptus* | 15^*^ | 8^*^ | 1.667^*^ | 4 | 1.667^*^ | 4 | 1.000 |
| *Scarites subterraneus* | 11 | 1 | 2.273 | 4^*^ | 2.273 | 4 | 1.000 |
| *Trichotichnus fulgens* | 10 | 3 | 2.500 | 7 | 2.500 | 7 | 1.000 |

**Table S5.** Boundary statistics calculated for environmental and ground beetle variables measured at the suburban site at the large spatial scale. Beetle community matrices were analyzed using raw abundances and species abundances weighted by the inverse of the species' proportion of total abundance at the site. *N_S_*: total number of boundaries and singletons; *N_1_*: number of singletons; *L_mean_*: mean length of boundaries and singletons (number of candidate boundary elements); *L_max_*: maximum length; *D_mean_*: mean diameter of boundaries (minimum number of links between the farthest pair of candidate boundary elements); *D_max_*: maximum diameter; *D/L*: mean diameter-to-length ratio. **p* < 0.10.

| Variable | *N_s_* | *N_1_* | *L_mean_* | *L_max_* | *D_mean_* | *D_max_* | *D/L* |
| --- | --- | --- | --- | --- | --- | --- | --- |
| Temperature | 4 | 2 | 1.750 | 3 | 1.750 | 3 | 1.000 |
| Humidity | 4 | 2 | 1.750 | 3 | 1.750 | 3 | 1.000 |
| Microrelief | 3 | 0^*^ | 2.333 | 3 | 2.333 | 3 | 1.000 |
| Slope | 4 | 1 | 1.750 | 2 | 1.750 | 2 | 1.000 |
| Leaf litter depth | 2^*^ | 0 | 3.500^*^ | 5^*^ | 3.500^*^ | 5^*^ | 1.000 |
| Canopy cover | 6^*^ | 5^*^ | 1.167^*^ | 2 | 1.167^*^ | 2 | 1.000 |
| Forb cover | 5 | 3 | 1.400 | 2 | 1.400 | 2 | 1.000 |
| Grass cover | 4 | 2 | 1.750 | 3 | 1.750 | 3 | 1.000 |
| Creeping forb cover | 5 | 3 | 1.400 | 2 | 1.400 | 2 | 1.000 |
| Shrub cover | 4 | 2 | 1.750 | 3 | 1.750 | 3 | 1.000 |
| Vine cover | 3 | 2 | 2.333 | 5^*^ | 2.333 | 5^*^ | 1.000 |
| Bare ground cover | 5 | 3 | 1.400 | 2 | 1.400 | 2 | 1.000 |
| Rock cover | 2 | 0 | 3.500 | 5 | 3.500 | 5 | 1.000 |
| Impervious surface cover | 4 | 2 | 1.750 | 3 | 1.750 | 3 | 1.000 |
| Coarse woody debris cover | 5 | 3 | 1.400 | 2 | 1.400 | 2 | 1.000 |
| Moss cover | 3 | 1 | 2.333 | 4 | 2.333 | 4 | 1.000 |
| Mulch cover | 3 | 1 | 2.333 | 4 | 2.333 | 4 | 1.000 |
| All environmental variables | 4 | 2 | 1.750 | 3 | 1.750 | 3 | 1.000 |
| Total abundance | 2^*^ | 1 | 3.500^*^ | 6^*^ | 3.500^*^ | 6^*^ | 1.000 |
| Total species richness | 4 | 1 | 1.750 | 2 | 1.750 | 2 | 1.000 |
| Total species evenness | 5 | 4 | 1.400 | 3 | 1.400 | 3 | 1.000 |
| Forest species abundance | 5 | 3 | 1.400 | 2 | 1.400 | 2 | 1.000 |
| Forest species richness | 5 | 4^*^ | 1.400 | 3 | 1.400 | 3 | 1.000 |
| Open-habitat species abundance | 3 | 0 | 2.333 | 3 | 2.333 | 3 | 1.000 |
| Open-habitat species richness | 3 | 1 | 2.333 | 4 | 2.333 | 4 | 1.000 |
| Generalist species abundance | 2 | 1 | 3.500 | 6^*^ | 3.500^*^ | 6^*^ | 1.000 |
| Generalist species richness | 4 | 2 | 1.750 | 3 | 1.750 | 3 | 1.000 |
| Generalist species evenness | 5 | 4 | 1.400 | 3 | 1.400 | 3 | 1.000 |
| All species | 3 | 2 | 2.333 | 5^*^ | 2.333 | 5^*^ | 1.000 |
| All species weighted | 3 | 1 | 2.333 | 4 | 2.000 | 3 | 0.917^*^ |
| Forest species | 5 | 3 | 1.400 | 2 | 1.400 | 2 | 1.000 |
| Forest species weighted | 5 | 4 | 1.400 | 3 | 1.400 | 3 | 1.000 |
| Open-habitat species | 3 | 0^*^ | 2.333 | 3 | 2.333 | 3 | 1.000 |
| Open-habitat species weighted | 2 | 1 | 3.500 | 6^*^ | 3.000 | 5 | 0.917 |
| Generalist species | 3 | 2 | 2.333 | 5 | 2.333 | 5^*^ | 1.000 |
| Generalist species weighted | 5 | 4 | 1.400 | 3 | 1.400 | 3 | 1.000 |
| *Agonum punctiforme* | 4 | 3 | 1.750 | 4 | 1.750 | 4 | 1.000 |
| *Amara aenea* | 3 | 0 | 2.333 | 3 | 2.333 | 3 | 1.000 |
| *Amara basillaris* | 4 | 1 | 1.750 | 2 | 1.750 | 2 | 1.000 |
| *Amara familiaris* | 4 | 1 | 1.750 | 2 | 1.750 | 2 | 1.000 |
| *Amara impuncticollis* | 4 | 2 | 1.750 | 3 | 1.750 | 3 | 1.000 |
| *Amphasia interstitialis* | 4 | 2 | 1.750 | 3 | 1.750 | 3 | 1.000 |
| *Anisodactylus dulcicollis* | 3 | 1 | 2.333 | 4 | 2.333 | 4 | 1.000 |
| *Anisodactylus nigerrimus* | 2 | 1 | 3.500 | 6 | 3.500 | 6 | 1.000 |
| *Anisodactylus opaculus* | 3 | 0 | 2.333 | 3 | 2.333 | 3 | 1.000 |
| *Anisodactylus ovularis* | 2 | 0 | 3.500 | 4 | 3.500 | 4 | 1.000 |
| *Anisodactylus rusticus* | 4 | 2 | 1.750 | 3 | 1.750 | 3 | 1.000 |
| *Chlaenius amoenus* | 3 | 2 | 2.333 | 5 | 2.333 | 5 | 1.000 |
| *Chlaenius emarginatus* | 5 | 3 | 1.400 | 2 | 1.400 | 2 | 1.000 |
| *Chlaenius prasinus* | 4 | 2 | 1.750 | 3 | 1.750 | 3 | 1.000 |
| *Chlaenius tricolor tricolor* | 4 | 1 | 1.750 | 2 | 1.750 | 2 | 1.000 |
| *Cyclotrachelus sigillatus* | 1 | 0 | 7.000 | 7 | 5.000 | 5 | 0.714^*^ |
| *Dicaelus dilatatus dilatatus* | 2 | 0 | 3.500 | 5 | 3.500 | 5 | 1.000 |
| *Galerita bicolor* | 5 | 3 | 1.400 | 2 | 1.400 | 2 | 1.000 |
| *Galerita janus* | 3 | 0 | 2.333 | 3 | 2.333 | 3 | 1.000 |
| *Harpalus pensylvanicus* | 3 | 0 | 2.333 | 3 | 2.333 | 3 | 1.000 |
| *Olisthopus parmatus* | 1 | 0 | 7.000 | 7 | 5.000 | 5 | 0.714^*^ |
| *Oodes fluvialis* | 4 | 1 | 1.750 | 2 | 1.750 | 2 | 1.000 |
| *Poecilus lucublandus lucublandus* | 2^*^ | 1 | 3.500^*^ | 6^*^ | 3.500^*^ | 6^*^ | 1.000 |
| *Pterostichus sculptus* | 4 | 3 | 1.750 | 4 | 1.750 | 4 | 1.000 |
| *Scarites subterraneus* | 1^*^ | 0 | 7.000^*^ | 7^*^ | 5.000^*^ | 5 | 0.714^*^ |
| *Scarites quadriceps* | 1 | 0 | 7.000 | 7 | 7.000^*^ | 7^*^ | 1.000 |
| *Stenolophus rotundatus* | 2^*^ | 1 | 3.500^*^ | 6^*^ | 3.500^*^ | 6^*^ | 1.000 |
| *Trichotichnus fulgens* | 3 | 1 | 2.333 | 4 | 2.333 | 4 | 1.000 |

**Table S6.** Boundary statistics calculated for environmental and ground beetle variables measured at the urban site at the small spatial scale. Beetle community matrices were analyzed using raw abundances and species abundances weighted by the inverse of the species' proportion of total abundance at the site. *N_S_*: total number of boundaries and singletons; *N_1_*: number of singletons; *L_mean_*: mean length of boundaries and singletons (number of candidate boundary elements); *L_max_*: maximum length; *D_mean_*: mean diameter of boundaries (minimum number of links between the farthest pair of candidate boundary elements); *D_max_*: maximum diameter; *D/L*: mean diameter-to-length ratio. **p* < 0.10.

| Variable | *N_s_* | *N_1_* | *L_mean_* | *L_max_* | *D_mean_* | *D_max_* | *D/L* |
| --- | --- | --- | --- | --- | --- | --- | --- |
| Temperature | 9 | 3 | 2.444^*^ | 6^*^ | 2.444^*^ | 6^*^ | 1.000 |
| Humidity | 10 | 3 | 2.200 | 6^*^ | 2.200 | 6^*^ | 1.000 |
| Microrelief | 12 | 6 | 1.917 | 4 | 1.917 | 4 | 1.000 |
| Slope | 9 | 4 | 2.556^*^ | 5 | 2.556^*^ | 5 | 1.000 |
| Leaf litter depth | 10 | 2 | 2.300^*^ | 4 | 2.300^*^ | 4 | 1.000 |
| Canopy cover | 8 | 2 | 2.875^*^ | 7^*^ | 2.875^*^ | 7^*^ | 1.000 |
| Forb cover | 12^*^ | 6 | 1.917 | 4 | 1.917 | 4 | 1.000 |
| Grass cover | 8 | 1^*^ | 2.875^*^ | 6^*^ | 2.750^*^ | 5 | 0.979^*^ |
| Shrub cover | 8 | 2 | 2.875^*^ | 5 | 2.875^*^ | 5 | 1.000 |
| Vine cover | 14^*^ | 8^*^ | 1.643 | 4 | 1.643 | 4 | 1.000 |
| Bare ground cover | 11^*^ | 5^*^ | 2.091 | 5 | 2.091 | 5 | 1.000 |
| Impervious surface cover | 8 | 2 | 2.875 | 8^*^ | 2.875 | 8^*^ | 1.000 |
| Coarse woody debris cover | 11 | 5 | 2.091 | 5 | 2.091 | 5 | 1.000 |
| All environmental variables | 12 | 7 | 1.917 | 4 | 1.833 | 4 | 0.979^*^ |
| Total abundance | 15^*^ | 6 | 1.667^*^ | 3^*^ | 1.667^*^ | 3^*^ | 1.000 |
| Total species richness | 13 | 5 | 1.923 | 4 | 1.923 | 4 | 1.000 |
| Total species evenness | 14^*^ | 8^*^ | 1.500 | 3 | 1.500 | 3 | 1.000 |
| Forest species abundance | 6^*^ | 1 | 4.167^*^ | 9 | 3.833^*^ | 9^*^ | 0.944^*^ |
| Forest species richness | 6^*^ | 1 | 4.167^*^ | 9 | 3.833^*^ | 9^*^ | 0.944^*^ |
| Open-habitat species abundance | 9 | 3 | 2.778 | 8 | 2.778 | 8 | 1.000 |
| Open-habitat species richness | 9 | 1 | 2.778 | 6 | 2.778 | 6 | 1.000 |
| Generalist species abundance | 15^*^ | 7 | 1.667^*^ | 4 | 1.667^*^ | 4 | 1.000 |
| Generalist species richness | 12 | 4 | 2.083 | 4 | 2.083 | 4 | 1.000 |
| Generalist species evenness | 14^*^ | 9^*^ | 1.500 | 4 | 1.500 | 4 | 1.000 |
| All species | 14 | 9 | 1.786 | 5 | 1.786 | 5 | 1.000 |
| All species weighted | 16 | 13 | 1.563 | 4 | 1.563 | 4 | 1.000 |
| Forest species | 11^*^ | 7 | 2.273^*^ | 7 | 2.182^*^ | 6 | 0.987 |
| Forest species weighted | 10 | 5 | 2.500 | 6 | 2.400 | 5 | 0.983 |
| Open-habitat species | 12 | 7 | 2.083 | 9^*^ | 2.000 | 8^*^ | 0.991 |
| Open-habitat species weighted | 13 | 8 | 1.923 | 5 | 1.923 | 5 | 1.000 |
| Generalist species | 15 | 11 | 1.667 | 5 | 1.667 | 5 | 1.000 |
| Generalist species weighted | 14 | 8 | 1.786 | 4 | 1.786 | 4 | 1.000 |
| *Agonum punctiforme* | 14^*^ | 10^*^ | 1.786^*^ | 5 | 1.786^*^ | 5 | 1.000 |
| *Amara aenea* | 9 | 4 | 2.778 | 8 | 2.778 | 8 | 1.000 |
| *Anisodactylus dulcicollis* | 12^*^ | 5 | 2.083^*^ | 6 | 2.083^*^ | 6 | 1.000 |
| *Anisodactylus furvus* | 5 | 0 | 5.000 | 7 | 5.000^*^ | 7 | 1.000^*^ |
| *Chlaenius tricolor tricolor* | 11^*^ | 4 | 2.273^*^ | 7 | 2.091^*^ | 5 | 0.974 |
| *Cyclotrachelus sigillatus* | 9 | 4 | 2.778 | 6 | 2.778 | 6 | 1.000 |
| *Harpalus longicollis* | 9 | 0^*^ | 2.778 | 4^*^ | 2.778 | 4 | 1.000 |
| *Harpalus pensylvanicus* | 8 | 3 | 3.125 | 7 | 3.125 | 7 | 1.000 |
| *Pterostichus sculptus* | 11 | 3 | 2.273 | 5 | 2.273 | 5 | 1.000 |
| *Scarites subterraneus* | 8 | 4 | 3.125 | 9^*^ | 3.125 | 9^*^ | 1.000 |
| *Sphaeroderus stenostomus lecontei* | 9 | 6^*^ | 2.778 | 13^*^ | 2.778 | 13^*^ | 1.000 |
| *Trichotichnus fulgens* | 11 | 4 | 2.273 | 7 | 2.273 | 7 | 1.000 |

**Table S7.** Boundary statistics calculated for environmental and ground beetle variables measured at the urban site at the large spatial scale. Beetle community matrices were analyzed using raw abundances and species abundances weighted by the inverse of the species' proportion of total abundance at the site. *N_S_*: total number of boundaries and singletons; *N_1_*: number of singletons; *L_mean_*: mean length of boundaries and singletons (number of candidate boundary elements); *L_max_*: maximum length; *D_mean_*: mean diameter of boundaries (minimum number of links between the farthest pair of candidate boundary elements); *D_max_*: maximum diameter; *D/L*: mean diameter-to-length ratio. **p* < 0.10.

| Variable | *N_s_* | *N_1_* | *L_mean_* | *L_max_* | *D_mean_* | *D_max_* | *D/L* |
| --- | --- | --- | --- | --- | --- | --- | --- |
| Temperature | 4 | 2 | 1.750 | 3 | 1.750 | 3 | 1.000 |
| Humidity | 2^*^ | 0^*^ | 3.500^*^ | 4 | 3.500^*^ | 4 | 1.000 |
| Microrelief | 2^*^ | 0 | 3.500^*^ | 4 | 3.500^*^ | 4 | 1.000 |
| Slope | 3 | 0^*^ | 2.333 | 3 | 2.333 | 3 | 1.000 |
| Leaf litter depth | 2^*^ | 0^*^ | 3.500^*^ | 4 | 3.500^*^ | 4 | 1.000 |
| Canopy cover | 4 | 2 | 1.750 | 3 | 1.750 | 3 | 1.000 |
| Forb cover | 3 | 0 | 2.333 | 3 | 2.333 | 3 | 1.000 |
| Grass cover | 4 | 2 | 1.750 | 3 | 1.750 | 3 | 1.000 |
| Creeping forb cover | 4 | 1 | 1.750 | 2 | 1.750 | 2 | 1.000 |
| Shrub cover | 5 | 3 | 1.400 | 2 | 1.400 | 2 | 1.000 |
| Vine cover | 4 | 2 | 1.750 | 3 | 1.750 | 3 | 1.000 |
| Bare ground cover | 5^*^ | 3 | 1.400^*^ | 2 | 1.400^*^ | 2 | 1.000 |
| Impervious surface cover | 4 | 2 | 1.750 | 3 | 1.750 | 3 | 1.000 |
| Coarse woody debris cover | 3 | 1 | 2.333 | 4 | 2.333 | 4 | 1.000 |
| Tree cover | 5 | 3 | 1.400 | 2 | 1.400 | 2 | 1.000 |
| Moss cover | 4 | 1 | 1.750 | 2 | 1.750 | 2 | 1.000 |
| All environmental variables | 5 | 3 | 1.400 | 2 | 1.400 | 2 | 1.000 |
| Total abundance | 4 | 1 | 1.750 | 2 | 1.750 | 2 | 1.000 |
| Total species richness | 4 | 2 | 1.750 | 3 | 1.750 | 3 | 1.000 |
| Total species evenness | 4 | 2 | 1.750 | 3 | 1.750 | 3 | 1.000 |
| Forest species abundance | 5 | 3 | 1.400 | 2 | 1.400 | 2 | 1.000 |
| Forest species richness | 5 | 3 | 1.400 | 2 | 1.400 | 2 | 1.000 |
| Open-habitat species abundance | 5 | 3 | 1.400 | 2 | 1.400 | 2 | 1.000 |
| Open-habitat species richness | 5 | 3 | 1.400 | 2 | 1.400 | 2 | 1.000 |
| Generalist species abundance | 4 | 1 | 1.750 | 2 | 1.750 | 2 | 1.000 |
| Generalist species richness | 2^*^ | 0^*^ | 3.500^*^ | 5^*^ | 3.500^*^ | 5^*^ | 1.000 |
| Generalist species evenness | 4 | 2 | 1.750 | 3 | 1.750 | 3 | 1.000 |
| All species | 5 | 3 | 1.400 | 2 | 1.400 | 2 | 1.000 |
| All species weighted | 4 | 2 | 1.750 | 3 | 1.750 | 3 | 1.000 |
| Forest species | 5 | 3 | 1.400 | 2 | 1.400 | 2 | 1.000 |
| Forest species weighted | 4 | 2 | 1.750 | 3 | 1.750 | 3 | 1.000 |
| Open-habitat species | 5 | 3 | 1.400 | 2 | 1.400 | 2 | 1.000 |
| Open-habitat species weighted | 3 | 2 | 2.333 | 5 | 2.333 | 5 | 1.000 |
| Generalist species | 5 | 3 | 1.400 | 2 | 1.400 | 2 | 1.000 |
| Generalist species weighted | 3 | 2 | 2.333 | 5 | 2.333 | 5 | 1.000 |
| *Agonum punctiforme* | 3 | 1 | 2.333 | 4 | 2.333 | 4 | 1.000 |
| *Amara aenea* | 4 | 1 | 1.750 | 2 | 1.750 | 2 | 1.000 |
| *Amara basillaris* | 2 | 0 | 3.500 | 5 | 3.500 | 5 | 1.000 |
| *Amara familiaris* | 5 | 3 | 1.400^*^ | 2 | 1.400^*^ | 2 | 1.000 |
| *Anisodactylus dulcicollis* | 4 | 1 | 1.750 | 2 | 1.750 | 2 | 1.000 |
| *Anisodactylus furvus* | 4 | 1 | 1.750 | 2 | 1.750 | 2 | 1.000 |
| *Anisodactylus rusticus* | 4 | 2 | 1.750 | 3 | 1.750 | 3 | 1.000 |
| *Calathus opaculus* | 3 | 1 | 2.333 | 3 | 2.333 | 3 | 1.000 |
| *Chlaenius tomentosus tomentosus* | 1^*^ | 0 | 7.000 | 7^*^ | 7.000^*^ | 7^*^ | 1.000 |
| *Chlaenius tricolor tricolor* | 5^*^ | 3 | 1.400^*^ | 2 | 1.400^*^ | 2 | 1.000 |
| *Cyclotrachelus sigillatus* | 4 | 3 | 1.750 | 4 | 1.750 | 4 | 1.000 |
| *Harpalus longicollis* | 2 | 0 | 3.500 | 5 | 3.500 | 5 | 1.000 |
| *Harpalus pensylvanicus* | 4 | 1 | 1.750 | 2^*^ | 1.750 | 2^*^ | 1.000 |
| *Harpalus protractus* | 3 | 0 | 2.333 | 3 | 2.333 | 3 | 1.000 |
| *Platynus decentis* | 5 | 3 | 1.400 | 2 | 1.400 | 2 | 1.000 |
| *Poecilus lucublandus lucublandus* | 4 | 1 | 1.750 | 2 | 1.750 | 2 | 1.000 |
| *Pterostichus sculptus* | 3 | 0 | 2.333 | 3 | 2.333 | 3 | 1.000 |
| *Scarites subterraneus* | 2 | 0 | 3.500 | 4 | 3.500 | 4 | 1.000 |
| *Sphaeroderus stenostomus lecontei* | 5^*^ | 4^*^ | 1.400^*^ | 3 | 1.400^*^ | 3 | 1.000 |
| *Trichotichnus autumnalis* | 3 | 1 | 2.333 | 4 | 2.333 | 4 | 1.000 |
| *Trichotichnus fulgens* | 2 | 0 | 3.500 | 5 | 3.500 | 5 | 1.000 |
