# Supplementary material for: Boundaries in ground beetle (Coleoptera: Carabidae) and environmental variables at the edges of forest patches with residential developments
Source: PeerJ. 2018 Jan 8;6:e4226. doi: 10.7717/peerj.4226 (PMC5764035; doi:10.7717/peerj.4226)
Supplement: Supplemental Information 3 — Overlap statistics calculated for pairs of environmental and ground beetle variables. Shown for each pair: Oe, the mean minimum Euclidean distance between each environmental candidate boundary element and the closest beetle candidate boundary element (m); Ob, the mean minimum Euclidean distance between each beetle candidate boundary element and the closest environmental candidate boundary element (m); Oeb, the overall mean minimum Euclidean distance between beetle and environmental candidate boundary elements (m); and Os, the number of beetle and environmental candidate boundary elements at the same locations. † Significantly low value (p < 0.10). ∗ Significantly high value (p < 0.10). [file peerj-06-4226-s003.docx]

**Table S8.** Overlap statistics calculated for pairs of environmental and ground beetle variables measured at the rural site at the small spatial scale. Shown for each pair: *O_e_*, the mean minimum Euclidean distance between each environmental candidate boundary element and the closest beetle candidate boundary element (m); *O_b_*, the mean minimum Euclidean distance between each beetle candidate boundary element and the closest environmental candidate boundary element (m); *O_eb_*, the overall mean minimum Euclidean distance between beetle and environmental candidate boundary elements (m); and *O_s_*, the number of beetle and environmental candidate boundary elements at the same locations. ^†^Significantly low value (*p* < 0.10). ^*^Significantly high value (*p* < 0.10).

| Beetle variable | Temperature | Leaf litter depth | Canopy cover | Forb cover | Grass cover | Vine cover | Rock cover | Coarse woody debris cover | All environmental variables |
| --- | --- | --- | --- | --- | --- | --- | --- | --- | --- |
| Total species evenness | 49,60,55,0^†^ | 31,27,29,3 | 45,61,53,2 | 45,51,48,2 | 35,39,37,3 | 23^†^,21^†^,22^†^,3 | 26,21,24,6 | 21^†^,22,21^†^,5 | 35,29^*^,32,4 |
| Open-habitat species abundance | 19,16^†^,17^†^,10^*^ | 37,17,27,6 | 18,13^†^,15^†^,11^*^ | 8^†^,9^†^,9^†^,13^*^ | 26,13^†^,19^†^,9 | 44^*^,28,36^*^,5 | 48^*^,30,39,6 | 77^*^,45^*^,61^*^,3 | 37,15^†^,26,9 |
| All species | 24,17^†^,20^†^,9 | 37^*^,18,28,5 | 22,11^†^,16^†^,13^*^ | 7^†^,7^†^,7^†^,16^*^ | 28,16^†^,22,8 | 38^*^,25,31^*^,8 | 46^*^,32,39^*^,3 | 74^*^,51^*^,62^*^,1^†^ | 43^*^,19,31^*^,6 |
| *Amara aenea* | 19,16^†^,17^†^,10^*^ | 37,17,27,6 | 18,13^†^,15^†^,11^*^ | 8^†^,9^†^,9^†^,13^*^ | 26,13^†^,19^†^,9 | 44, ^*^28,36^*^,5 | 48^*^,30,39^*^,6 | 77^*^,45^*^,61^*^,3 | 37,15^†^,26,9 |
| *Harpalus pensylvanicus* | 13^†^,16^†^,15^†^,9^*^ | 19^†^,12^†^,16^†^,10^*^ | 9^†^,11^†^,10^†^,14^*^ | 18,21^†^,20^†^,8 | 20,16^†^,18^†^,7 | 48^*^,31,40^*^,2 | 27,21,24,8 | 67^*^,47^*^,57^*^,3 | 31,14^†^,22,10 |
| *Sphaeroderus stenostomus lecontei* | 85^*^,81^*^,83^*^,1^†^ | 55^*^,28,42^*^,5 | 85^*^,90^*^,87^*^,0^†^ | 88^*^,87^*^,87^*^,0^†^ | 74^*^,65^*^,70^*^,2 | 44^*^,32^*^,38^*^,2 | 51^*^,37^*^,44^*^,5 | 29,11^†^,20^†^,13^*^ | 67^*^,22,45^*^,5 |
| *Trichotichnus fulgens* | 15^†^,31,24^†^,9^*^ | 29,24,27,3 | 19,31,25,8 | 16^†^,29,22^†^,8 | 18^†^,24,21^†^,9 | 21,23,22,7 | 28,32,30,4 | 28,35,32,3 | 24,20,22,6 |

**Table S9.** Overlap statistics calculated for pairs of environmental and ground beetle variables measured at the rural site at the large spatial scale. Shown for each pair: *O_e_*, the mean minimum Euclidean distance between each environmental candidate boundary element and the closest beetle candidate boundary element (m); *O_b_*, the mean minimum Euclidean distance between each beetle candidate boundary element and the closest environmental candidate boundary element (m); *O_eb_*, the overall mean minimum Euclidean distance between beetle and environmental candidate boundary elements (m); and *O_s_*, the number of beetle and environmental candidate boundary elements at the same locations. ^†^Significantly low value (*p* < 0.10). ^*^Significantly high value (*p* < 0.10).

| Beetle variable | Temperature | Humidity | Slope | Leaf litter depth | Forb cover | Bare ground cover |
| --- | --- | --- | --- | --- | --- | --- |
| Total species evenness | 70,90^*^,80,0 | 64^*^,56,60,0 | 33,37,35,2 | 43,41,42,0 | 73,90^*^,81^*^,0 | 42,29,35,1 |
| Generalist species abundance | 13^†^,17^†^,15^†^,4^*^ | 33,28,31,2 | 46,50,48,1 | 46,55^*^,51,1 | 13^†^,17^†^,15^†^,4^*^ | 53,33,43,2 |
| Generalist species richness | 22,37^†^,30^†^,2 | 26,32,29,2 | 23,40,31,2 | 37,39,38,1 | 26,40,33,1 | 29,28,28,2 |
| Generalist species evenness | 109^*^,100^*^,105^*^,0 | 89^*^,65^*^,77^*^,0 | 32,32,32,2 | 55,43,49,0 | 112^*^,100^*^,106^*^,0 | 76^*^,35,55^*^,1 |
| All species | 7^†^,8^†^,8^†^,5^*^ | 20^†^,18^†^,19^†^,3^*^ | 47,43,45,1 | 52,56^*^,54^*^,1 | 7^†^,8^†^,8^†^,5^*^ | 45,30,37,2 |
| All species weighted | 7^†^,12^†^,9^†^,5^*^ | 18^†^,18^†^,18^†^,4^*^ | 39,37,38,2 | 44,55^*^,50,1 | 7^†^,12^†^,9^†^,5^*^ | 51,33,42,2 |
| Generalist species | 7^†^,8^†^,8^†^,5^*^ | 20^†^,18^†^,19^†^,3^*^ | 47,43,45,1 | 52,56^*^,54^*^,1 | 7^†^,8^†^,8^†^,5^*^ | 45,30,37,2 |
| *Anisodactylus dulcicollis* | 25,28^†^,26^†^,2 | 28,30,29,2 | 19,21^†^,20,3 | 33,26,30,3 | 21,24^†^,23^†^,3 | 56,44,50,0 |
| *Anisodactylus opaculus* | 16^†^,32,24,3 | 30,39,34,2 | 47,55,51,1 | 48,48,48,1 | 13^†^,29,21^†^,4^*^ | 46,34,40,2 |
| *Anisodactylus rusticus* | 16^†^,24^†^,20^†^,3 | 20^†^,24^†^,22^†^,3 | 19,18^†^,19,3 | 33,25,29,3 | 19,24^†^,22^†^,3 | 46,37,42,1 |
| *Chlaenius prasinus* | 64^*^,69^*^,67^*^,0^†^ | 46^*^,38^†^,42^*^,1^†^ | 27^*^,27^†^,27^†^,2^†^ | 42^*^,32^†^,37^*^,1^†^ | 67^*^,69^*^,68^*^,0^†^ | 49^*^,32^†^,40^*^,1^†^ |
| *Galerita janus* | 31,52,42,2 | 37,49,43,2 | 18,22,20,2 | 43,27,35,2 | 33,52,43,2 | 67^*^,53^*^,60^*^,1 |
| *Lebia vittata* | 0,0^†^,0^†^,7 | 15^*^,7^†^,11^†^,5^†^ | 59^*^,36^†^,48^*^,1^†^ | 59^*^,56^*^,57^*^,0^†^ | 4^*^,4^†^,4^†^,6^†^ | 49^*^,22^†^,36^*^,3^†^ |

**Table S10.** Overlap statistics calculated for pairs of environmental and ground beetle variables measured at the suburban site at the small spatial scale. Shown for each pair: *O_e_*, the mean minimum Euclidean distance between each environmental candidate boundary element and the closest beetle candidate boundary element (m); *O_b_*, the mean minimum Euclidean distance between each beetle candidate boundary element and the closest environmental candidate boundary element (m); *O_eb_*, the overall mean minimum Euclidean distance between beetle and environmental candidate boundary elements (m); and *O_s_*, the number of beetle and environmental candidate boundary elements at the same locations. ^†^Significantly low value (*p* < 0.10). ^*^Significantly high value (*p* < 0.10).

| Beetle variable | Slope | Leaf litter depth | Forb cover | Grass cover | Creeping forb cover | Shrub cover | Moss cover | All environmental variables |
| --- | --- | --- | --- | --- | --- | --- | --- | --- |
| Total species evenness | 28,27,27,5 | 24^†^,26,25,2 | 23,28,25,5 | 24^†^,27,25,4 | 25,33,29,3 | 19^†^,19,19^†^,7^*^ | 24^†^,29,26,9^*^ | 29,31^*^,30,6 |
| Generalist species evenness | 17^†^,21,19^†^,7^*^ | 24^†^,24,24,3 | 15^†^,22,19^†^,6 | 18^†^,20,19^†^,7^*^ | 21^†^,29,25,4 | 20^†^,24,22^†^,7^*^ | 19^†^,24,21^†^,8^*^ | 18^†^,22,20^†^,7^*^ |
| Open-habitat species | 36,20,28,8 | 54^*^,49^*^,51^*^,0^†^ | 54^*^,30,42^*^,2 | 57^*^,40^*^,48^*^,6 | 70^*^,37,53^*^,3 | 57^*^,56^*^,56^*^,3 | 27,21,24,8 | 47^*^,28,37,7 |
| Generalist species weighted | 21,23,22,5 | 31,31^*^,31^*^,3^†^ | 16,24,20,8 | 22,26,24,8 | 20,31,25,5 | 31,37^*^,34^*^,4 | 24,30,27,3 | 20,20,20,8 |
| *Amara aenea* | 36,25,30,6 | 50^*^,46^*^,48^*^,0^†^ | 52^*^,29,41^*^,2 | 56^*^,39^*^,48^*^,6 | 69^*^,38,53^*^,3 | 55^*^,51^*^,53^*^,3 | 27,24,25,8 | 47^*^,32,40^*^,5 |
| *Anisodactylus dulcicollis* | 30,22,26,7 | 41^*^,34^*^,38^*^,4 | 37^*^,22,29,8 | 46^*^,30,38^*^,5 | 57^*^,24,40^*^,4 | 50^*^,45^*^,47^*^,2^†^ | 32,24,28,7 | 39^*^,24,31,9 |
| *Dicaelus dilatatus dilatatus* | 44^*^,36,40^*^,3 | 21,14^†^,17^†^,7 | 24,16^†^,20,8 | 20,12^†^,16^†^,10 | 23,28,25,4 | 21,13^†^,17^†^,9 | 64^*^,44^*^,54^*^,1^†^ | 43,33,38,3 |
| *Poecilus lucublandus lucublandus* | 62^*^,29,46^*^,4 | 40^*^,20,30,4 | 39,12^†^,26,10^*^ | 32,14^†^,23,9 | 27,7^†^,17^†^,17^*^ | 26,13^†^,20,9 | 77^*^,50^*^,64^*^,0^†^ | 54^*^,21,38,8 |
| *Pterostichus sculptus* | 32,28,30,1^†^ | 33,32^*^,32^*^,4 | 22,24,23,8 | 20,28,24,7 | 21,28,25,10^*^ | 16^†^,25,21,7 | 27,36,32,5 | 28,25,26,7 |
| *Scarites subterraneus* | 30,17^†^,23,7 | 44^*^,33^*^,39^*^,4 | 30,28,29,4 | 40,46^*^,43^*^,0^†^ | 30,36,33,3 | 46^*^,54^*^,50^*^,0^†^ | 21,16^†^,19^†^,11^*^ | 26,22,24,6 |

**Table S11.** Overlap statistics calculated for pairs of environmental and ground beetle variables measured at the suburban site at the large spatial scale. Shown for each pair: *O_e_*, the mean minimum Euclidean distance between each environmental candidate boundary element and the closest beetle candidate boundary element (m); *O_b_*, the mean minimum Euclidean distance between each beetle candidate boundary element and the closest environmental candidate boundary element (m); *O_eb_*, the overall mean minimum Euclidean distance between beetle and environmental candidate boundary elements (m); and *O_s_*, the number of beetle and environmental candidate boundary elements at the same locations. ^†^Significantly low value (*p* < 0.10). ^*^Significantly high value (*p* < 0.10).

| Beetle variable | Microrelief | Leaf litter depth | Canopy cover | Vine cover |
| --- | --- | --- | --- | --- |
| Total abundance | 16^†^,14^†^,15^†^,4^*^ | 43,29,36,2 | 37,29,33,2 | 71^*^,51,61^*^,1 |
| Forest species richness | 61,63,62,0 | 37,44,40,1 | 47,58,53,2 | 29,32,30,2 |
| Generalist species abundance | 16^†^,15^†^,15^†^,4 | 43,34,38,2 | 44,29,36,2 | 71^*^,48,59^*^,1 |
| All species | 16^†^,16^†^,16^†^,4^*^ | 43,37,40,2 | 45,27,36,2 | 71^*^,42,57^*^,1 |
| All species weighted | 37,42,39,0 | 44,43,43,0 | 49,51,50,1 | 52,40,46,1 |
| Open-habitat species | 29,24,26,2 | 32,24,28,2 | 44,48,46,1 | 35,24,29,2 |
| Open-habitat species weighted | 55,47,51,0 | 64^*^,52,58^*^,0 | 39,45,42,2 | 65,34,50,1 |
| Generalist species | 16^†^,16^†^,16^†^,4^*^ | 43,37,40,2 | 45,27,36,2 | 71^*^,42,57,1 |
| *Cyclotrachelus sigillatus* | 42,31^†^,37^†^,2 | 35,31,33,2 | 74^*^,67^*^,70^*^,0 | 46,42^*^,44^*^,0^†^ |
| *Olisthopus parmatus* | 42,31^†^,37^†^,2 | 35,31,33,2 | 74^*^,67^*^,70^*^,0 | 46,42^*^,44^*^,0^†^ |
| *Poecilus lucublandus lucublandus* | 92^*^,66,79^*^,0 | 62^*^,55^*^,58^*^,0 | 103^*^,79^*^,91^*^,0 | 42,32,37,1 |
| *Scarites subterraneus* | 16^†^,11^†^,13^†^,4^*^ | 43,28,35,2 | 50,33,41,1 | 71^*^,42,57,1 |
| *Scarites quadriceps* | 16,11^†^,13^†^,4 | 43,28,35,2 | 50,33,41,1 | 71,42,57,1 |
| *Stenolophus rotundatus* | 38,36,37,1 | 72^*^,46,59^*^,0 | 29^†^,14^†^,21^†^, 4^*^ | 97^*^,74^*^,85^*^,0 |

**Table S12.** Overlap statistics calculated for pairs of environmental and ground beetle variables measured at the urban site at the small spatial scale. Shown for each pair: *O_e_*, the mean minimum Euclidean distance between each environmental candidate boundary element and the closest beetle candidate boundary element (m); *O_b_*, the mean minimum Euclidean distance between each beetle candidate boundary element and the closest environmental candidate boundary element (m); *O_eb_*, the overall mean minimum Euclidean distance between beetle and environmental candidate boundary elements (m); and *O_s_*, the number of beetle and environmental candidate boundary elements at the same locations. ^†^Significantly low value (*p* < 0.10). ^*^Significantly high value (*p* < 0.10).

| Beetle variable | Temperature | Humidity | Slope | Leaf litter depth | Canopy cover | Forb cover | Grass cover | Shrub cover | Vine cover | Bare ground cover | Impervious surface cover | All environmental variables |
| --- | --- | --- | --- | --- | --- | --- | --- | --- | --- | --- | --- | --- |
| Total abundance | 19,36,28,8 | 17,28,23,10^*^ | 33^*^,47,41,4 | 31,33,32,5 | 17,35,26,8 | 19,27,23,9 | 16,23,20^†^,9 | 27,39,33,8 | 40^*^,34,37^*^,6 | 17,21,19,6 | 13,25^†^,19^†^,10^*^ | 16,19,17,10 |
| Total evenness | 25,45,35,4 | 29,33,31,4 | 24^†^,45,34,3 | 18^†^,23,20^†^,6^*^ | 24,45,34,6 | 21^†^,41,30,6 | 21^†^,27,24,7 | 26,40,33,2 | 18^†^,17^†^,17^†^,7^*^ | 18^†^,20,19^†^,8^*^ | 22,39,30,7 | 26,24,25,7 |
| Forest species abundance | 65^*^,79^*^,72^*^,0^†^ | 65^*^,63^*^,64^*^,0^†^ | 44^*^,35,39,1 | 23,22,22,9 | 67^*^,84^*^,76^*^,0^†^ | 61^*^,61^*^,61^*^,1 | 54^*^,63^*^,59^*^,0^†^ | 34,15^†^,24,10^*^ | 29,20,24,6 | 40^*^,35,37,4 | 50^*^,64^*^,57^*^,1 | 60^*^,43^*^,51^*^,0^†^ |
| Forest species richness | 65^*^,79^*^,72^*^,0^†^ | 65^*^,63^*^,64^*^,0^†^ | 44^*^,35,39,1 | 23,22,22,9 | 67^*^,84^*^,76^*^,0^†^ | 60^*^,61^*^,61^*^,1 | 54^*^,63^*^,59^*^,0^†^ | 34,15^†^,24,10^*^ | 29,20,24,6 | 40^*^,35,37,4 | 50^*^,64^*^,57^*^,1 | 60^*^,43^*^,51^*^,0^†^ |
| Generalist species abundance | 25,50,38,5 | 22,37,30,8 | 30,50,40,4 | 32,31,31,6 | 22,51,37,5 | 22,40,31,6 | 19,34,27,7 | 23,42,33,7 | 38^*^,29,33,6 | 20,26,23,5 | 15,38,27,9 | 18,21,20,9 |
| Generalist species evenness | 19^†^,39,29,7^*^ | 19^†^,25,22^†^,8^*^ | 28,49,38,4 | 30,32,31,4 | 18^†^,41,29,7 | 16^†^,39,27,8^*^ | 15^†^,23,19^†^,10^*^ | 20^†^,34,27,7 | 27^†^,32,29,5 | 22,27,24,4 | 19,39,29,5 | 16^†^,21,19^†^,10^*^ |
| Forest species | 65^*^,82^*^,74^*^,0^†^ | 65^*^,64^*^,64^*^,0^†^ | 44^*^,36,40,1 | 23,22,22,9 | 67^*^,87^*^,77^*^,0^†^ | 61^*^,64^*^,62^*^,1 | 54^*^,63^*^,59^*^,0^†^ | 34,15^†^,24,10^*^ | 29,19,24,6 | 40^*^,33,36,4 | 50^*^,65^*^,58^*^,1 | 60^*^,43^*^,51^*^,0^†^ |
| Open-habitat species | 27,34,31,5 | 29,23^†^,26,6 | 25,31,28,8 | 24,26,25,8 | 14^†^,18^†^,16^†^,9 | 30,38,34,6 | 23,20^†^,22^†^,8 | 28,31,30,4 | 29,22,25,8 | 16^†^,14^†^,15^†^,10^*^ | 11^†^,14^†^,13^†^,10^*^ | 22,20,21,7 |
| *Agonum punctiforme* | 15^†^,24^†^,20^†^,8 | 18,26,22,7 | 30,38,34,5 | 33,32,32,6 | 13^†^,17^†^,15^†^,11^*^ | 21,16^†^,19^†^,11^*^ | 14^†^,16^†^,15^†^,10^*^ | 41^*^,35,38,6 | 47^*^,36^*^,42^*^,4 | 19,13^†^,16^†^,10^*^ | 13^†^,16^†^,14^†^, 10^*^ | 20,22,21,6 |
| *Anisodactylus dulcicollis* | 16^†^,16^†^,16^†^,11^*^ | 18,19^†^,19^†^,10^*^ | 43^*^,42,42,5 | 46^*^,35,40^*^,3 | 12^†^,14^†^,13^†^,12^*^ | 22,10^†^,16^†^,13^*^ | 16^†^,11^†^,14^†^,11^*^ | 49^*^,41,44^*^,7 | 62^*^,38^*^,49^*^,2 | 23,15^†^,19^†^,7 | 6^†^,8^†^,7^†^,15^*^ | 19,13^†^,16^†^,13^*^ |
| *Anisodactylus furvus* | 24,33^†^,29^†^,7 | 27,21^†^,24^†^,8 | 20,27^†^,23^†^,12 | 22,19^†^,21^†^,9 | 26,35^†^,31^†^,6 | 32,44,38,4 | 21,19^†^,20^†^,9 | 30,34,32,3 | 27,15^†^,21^†^,9 | 19,16^†^,17^†^,8 | 20,33^†^,26^†^,8 | 29,19^†^,24^†^,8 |
| *Chlaenius tricolor tricolor* | 18^†^,24^†^,21^†^,6 | 17^†^,17^†^,17^†^,7 | 32,61^*^,47,4 | 37,31,34,5 | 18,24^†^,21^†^,6 | 28,30,29,7 | 11^†^,12^†^,11^†^,12^*^ | 46^*^,55^*^,51^*^,2 | 47^*^,32,39,5 | 18^†^,19^†^,19^†^,8 | 11^†^,24^†^,18^†^,9 | 16^†^,15^†^,16^†^,9^*^ |
| *Harpalus longicollis* | 15^†^,13^†^,14^†^,11^*^ | 17^†^,14^†^,15^†^,10^*^ | 41^*^,53,47^*^,4 | 51^*^,50^*^,50^*^,1^†^ | 14^†^,12^†^,13^†^,11^*^ | 21,13^†^,17^†^,13^*^ | 16^†^,13^†^,15^†^,11^*^ | 53^*^,46,49^*^,8 | 66^*^,48^*^,57^*^,2 | 28,20,24,5 | 10^†^,13^†^,11^†^,12^*^ | 19,12^†^,15^†^,12^*^ |
| *Scarites subterraneus* | 16^†^,19^†^,18^†^,7 | 17,23^†^,20^†^,7 | 40^*^,40,40,3 | 40^*^,37,38^*^,4 | 12^†^,16^†^,14^†^,10^*^ | 22,11^†^,16^†^,11^*^ | 16^†^,15^†^,16^†^,8 | 47^*^,43,45^*^,3 | 56^*^,32,43^*^,2 | 20,12^†^,16^†^,10^*^ | 9^†^,13^†^,11^†^,11^*^ | 20,18^†^,19,8 |
| *Sphaeroderus stenostomus lecontei* | 80^*^,77^*^,78^*^,0^†^ | 80^*^,66^*^,73^*^,0^†^ | 42,21^†^,31,5 | 32,17^†^,24,8 | 87^*^,86^*^,86^*^,0^†^ | 80^*^,57^*^,68^*^,1^†^ | 74^*^,73^*^,73^*^,0^†^ | 44,26,34,2 | 39,22,30,5 | 63^*^,50^*^,56^*^,0^†^ | 72^*^,72^*^,72^*^,0^†^ | 73^*^,41^*^,57^*^,1^†^ |

**Table S13.** Overlap statistics calculated for pairs of environmental and ground beetle variables measured at the urban site at the large spatial scale. Shown for each pair: *O_e_*, the mean minimum Euclidean distance between each environmental candidate boundary element and the closest beetle candidate boundary element (m); *O_b_*, the mean minimum Euclidean distance between each beetle candidate boundary element and the closest environmental candidate boundary element (m); *O_eb_*, the overall mean minimum Euclidean distance between beetle and environmental candidate boundary elements (m); and *O_s_*, the number of beetle and environmental candidate boundary elements at the same locations. ^†^Significantly low value (*p* < 0.10). ^*^Significantly high value (*p* < 0.10).

| Beetle variable | Humidity | Microrelief | Slope | Leaf litter depth | Bare ground cover |
| --- | --- | --- | --- | --- | --- |
| Generalist species richness | 27,21^†^,24,3 | 35,26,31,2 | 61,44,52,0 | 28,16^†^,22^†^,4^*^ | 52,46,49,2 |
| *Amara familiaris* | 42,36,39,1 | 75^*^,68^*^,71^*^,0 | 56,36,46,2 | 41,28,34,2 | 28,20,24,3 |
| *Chlaenius tomentosus tomentosus* | 53^*^,32^†^,43^*^,2^†^ | 92^*^,66^*^,79^*^,0^†^ | 89^*^,71^*^,80^*^,0^†^ | 43^*^,32^†^,38^*^,2^†^ | 28^*^,17^†^,22^†^,3^†^ |
| *Chlaenius tricolor tricolor* | 35,32,33,2 | 61,59,60,1 | 54,58^*^,56,1 | 24^†^,27,26^†^,2 | 15^†^,18^†^,17^†^,4^*^ |
| *Harpalus pensylvanicus* | 34,29,32,2 | 47,45,46,1 | 37,30,34,2 | 29,18^†^,24^†^,3 | 42,33,37,2 |
| *Sphaeroderus stenostomus lecontei* | 55,61^*^,58,0 | 33,41,37,1 | 41,26,33,2 | 65,63^*^,64^*^,0 | 96^*^,95^*^,95^*^,0 |
