# Supplementary material for: Boundaries in ground beetle (Coleoptera: Carabidae) and environmental variables at the edges of forest patches with residential developments
Source: PeerJ. 2018 Jan 8;6:e4226. doi: 10.7717/peerj.4226 (PMC5764035; doi:10.7717/peerj.4226)

Figure S1. The Delaunay triangles (black lines) and their centroids (white diamonds) at each site at two spatial scales. Black dots are trap locations at the small scale and the centroids of trios of adjacent trap locations at the large scale. Edges are indicated by dashed black lines and correspond to the property lines between County-owned forest and private development.

A) Rural site

Small scale

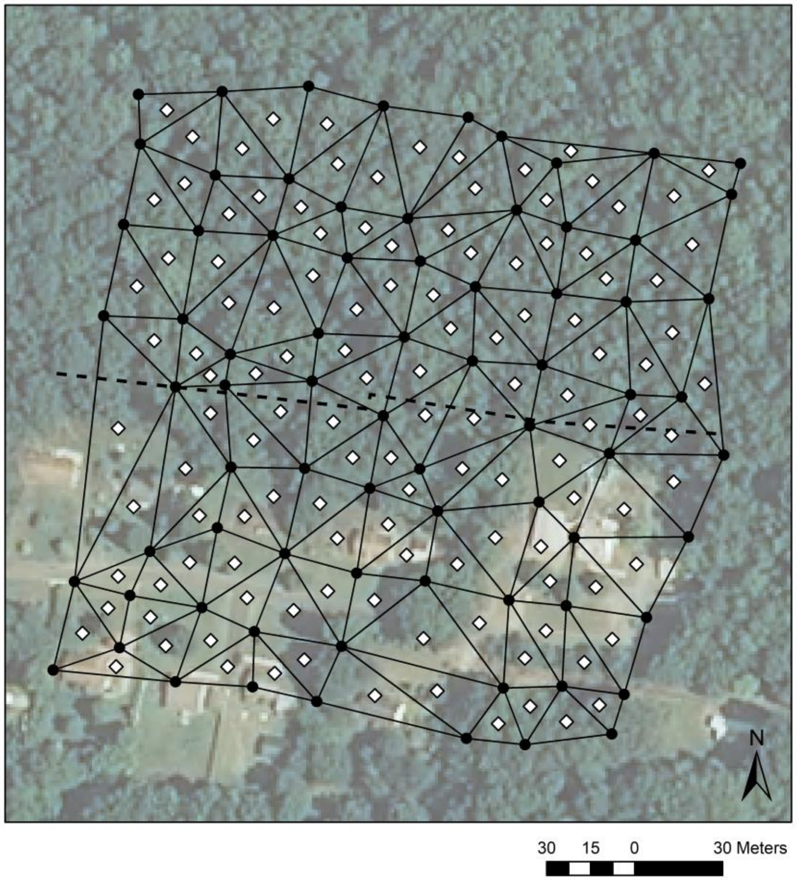

Large scale

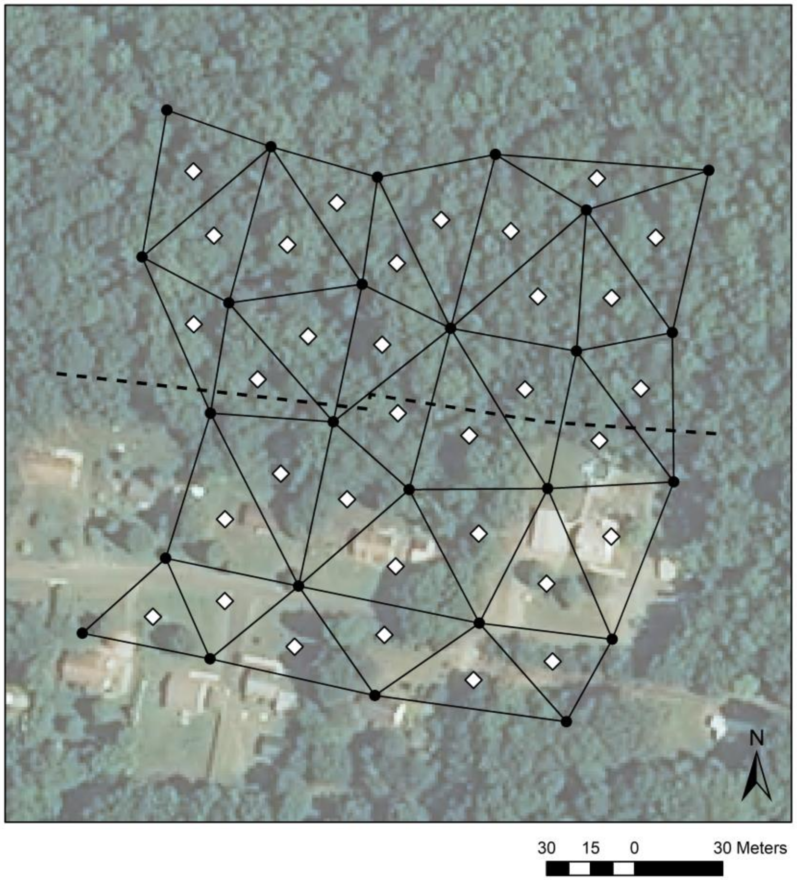

B) Suburban site

Small scale

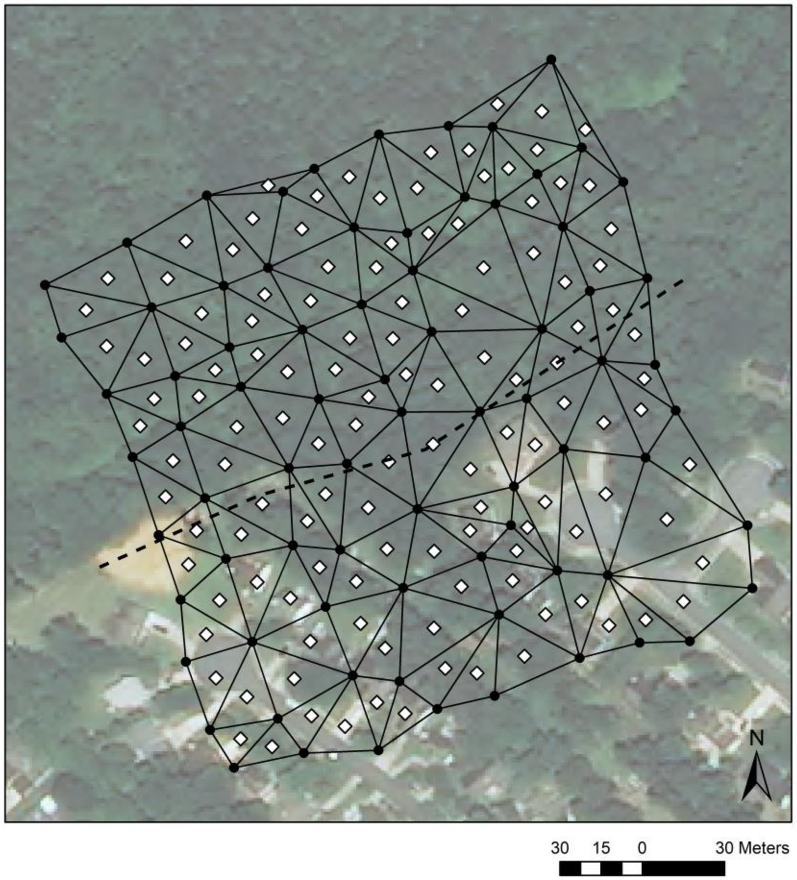

Large scale

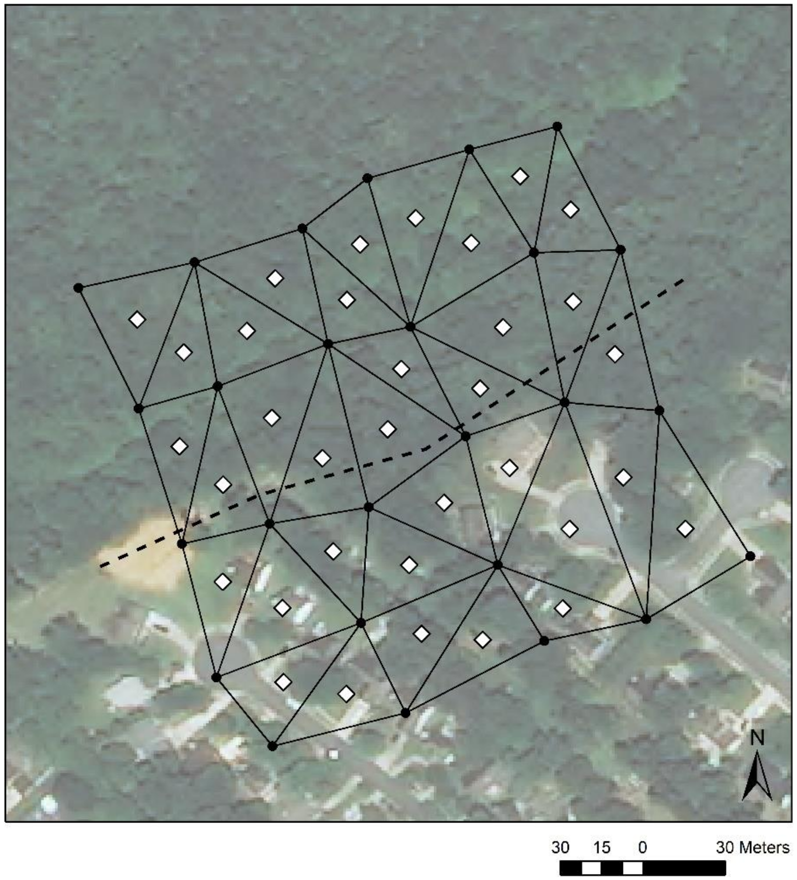

C) Urban site

Small scale

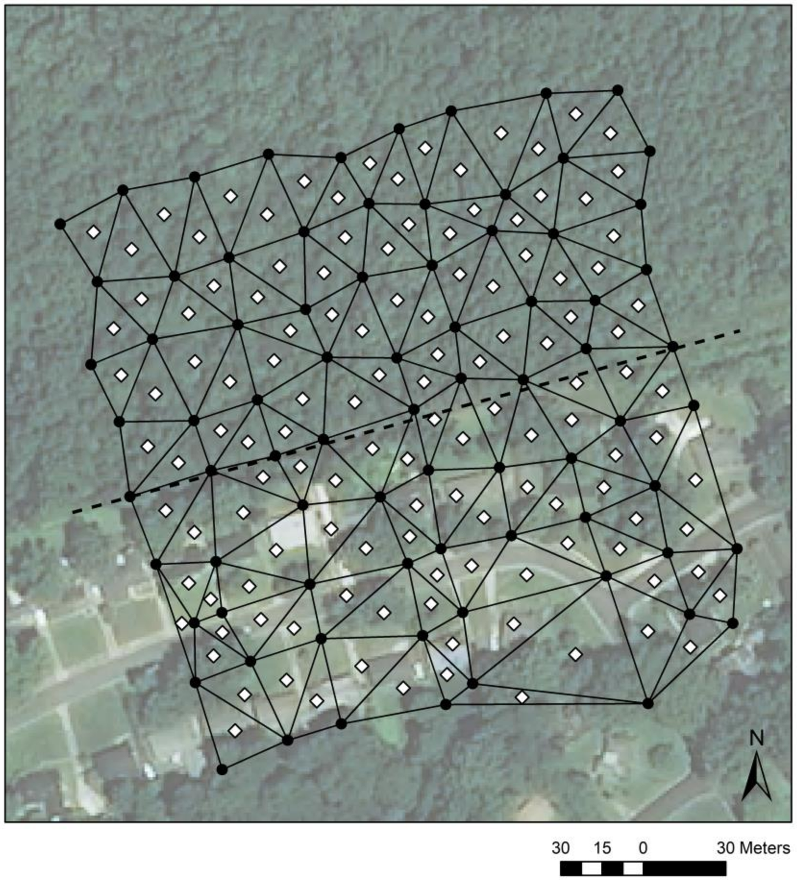

Large scale

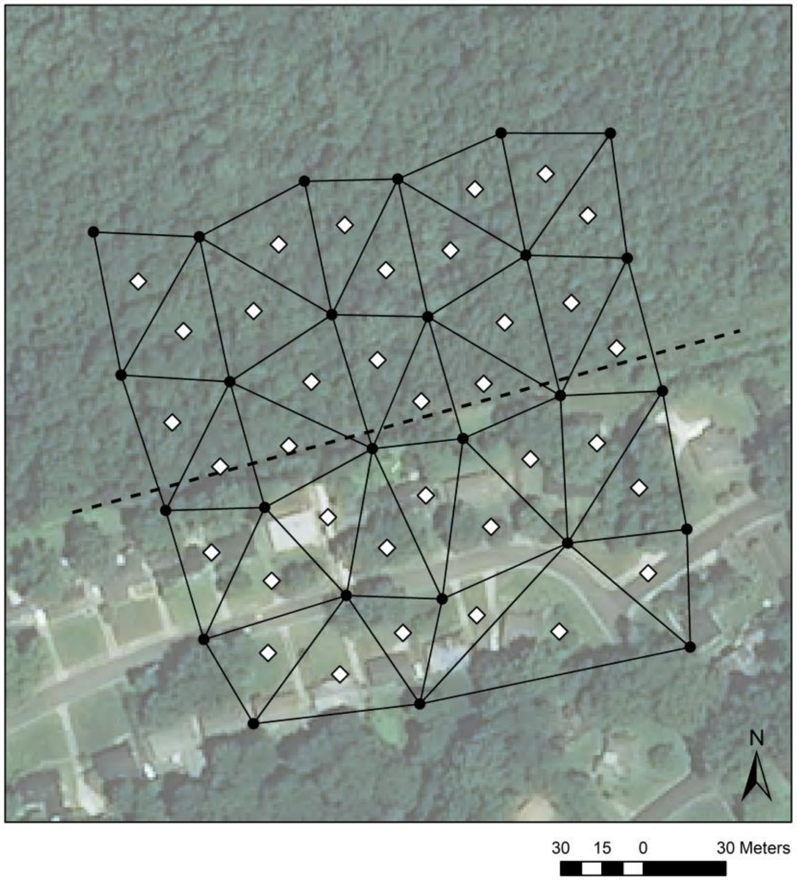

Supplement: Supplemental Information 4 — The Delaunay triangles (black lines) and their centroids (white diamonds) at each site at two spatial scales. Black dots are trap locations at the small scale and the centroids of trios of adjacent trap locations at the large scale. Edges are indicated by dashed black lines and correspond to the property lines between County-owned forest and private development. [file peerj-06-4226-s004.pdf]
