# Supplementary material for: Boundaries in ground beetle (Coleoptera: Carabidae) and environmental variables at the edges of forest patches with residential developments
Source: PeerJ. 2018 Jan 8;6:e4226. doi: 10.7717/peerj.4226 (PMC5764035; doi:10.7717/peerj.4226)

Figures S2-S92. Boundaries (yellow lines) and singletons (yellow stars) in environmental and ground beetle variables with one or more significant boundary statistics. Figures are named by site (rural, suburban, or urban), spatial scale (small or large), and variable. Black dots are trap locations at the small scale and the centroids of trios of adjacent trap locations at the large scale and are labeled with variable values (temperature in degrees Celsius; humidity as a percentage; microrelief and slope as ordinal indices; leaf litter depth in cm; covers as percentages; all environmental variables as a standardized average of individual variables; abundances and richnesses as numbers of individuals and species, respectively, per trapping period; evennesses as Berger-Parker indices; missing values indicated by -9999). Edges are indicated by black lines and correspond to the property lines between County-owned forest and private development. Beetle community matrices were analyzed using raw abundances and species abundances weighted by the inverse of the species' proportion of total abundance at the site.

Figure S2. Rural_small_Temperature

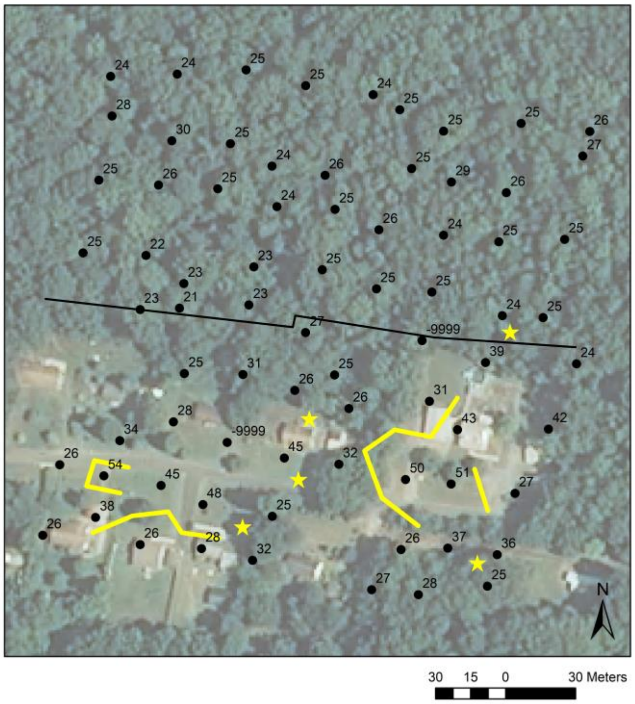

Figure S3. Rural_small_Leaf litter depth

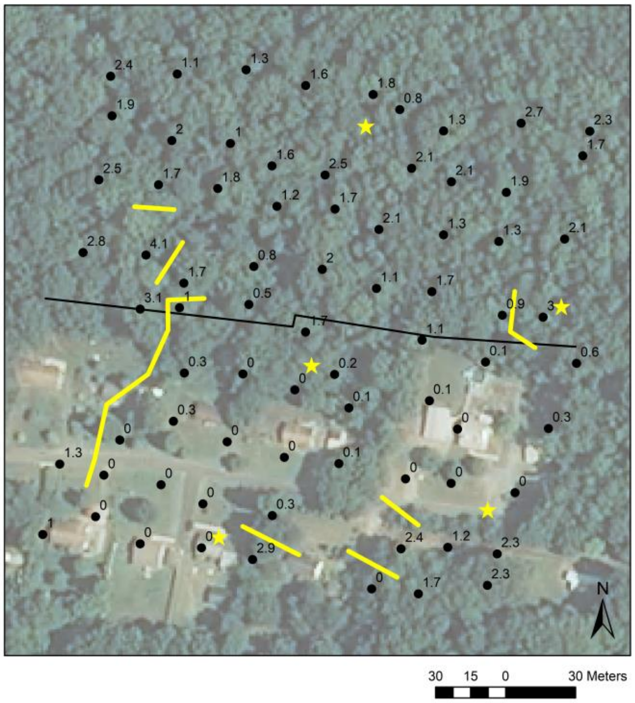

Figure S4. Rural_small_Forb cover

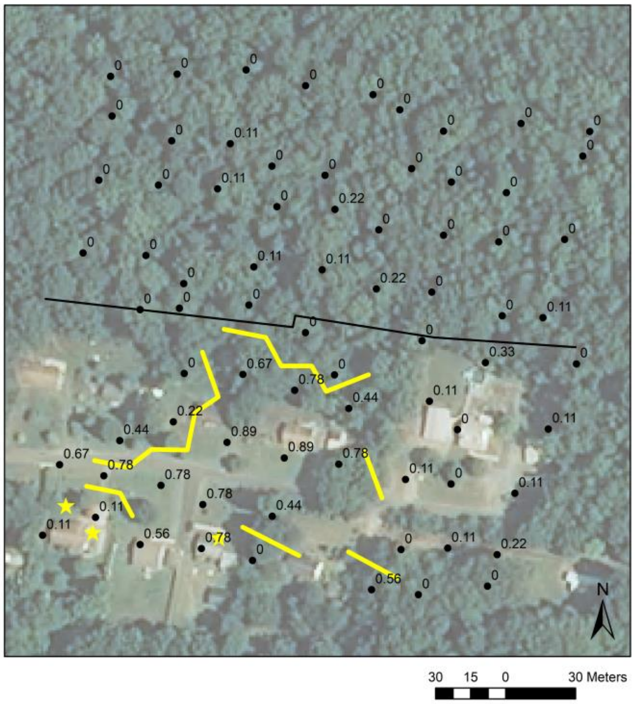

Figure S5. Rural_small_Rock cover

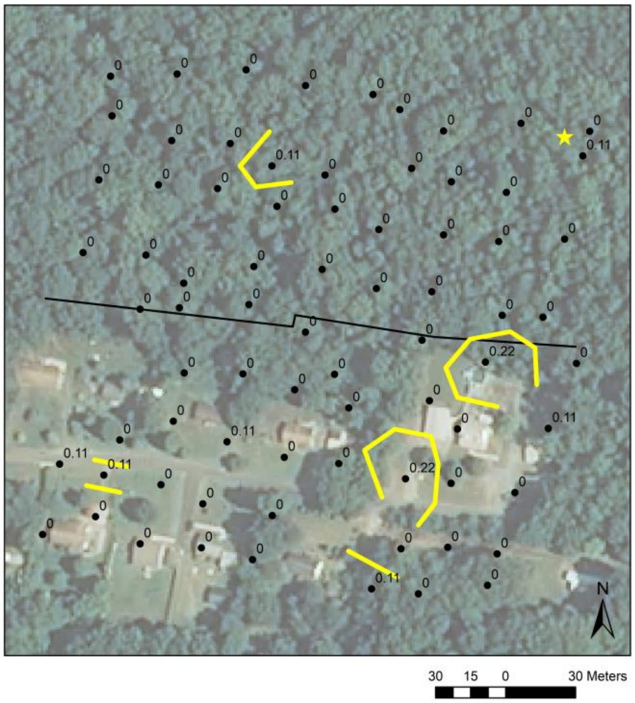

Figure S6. Rural_small_Vine cover

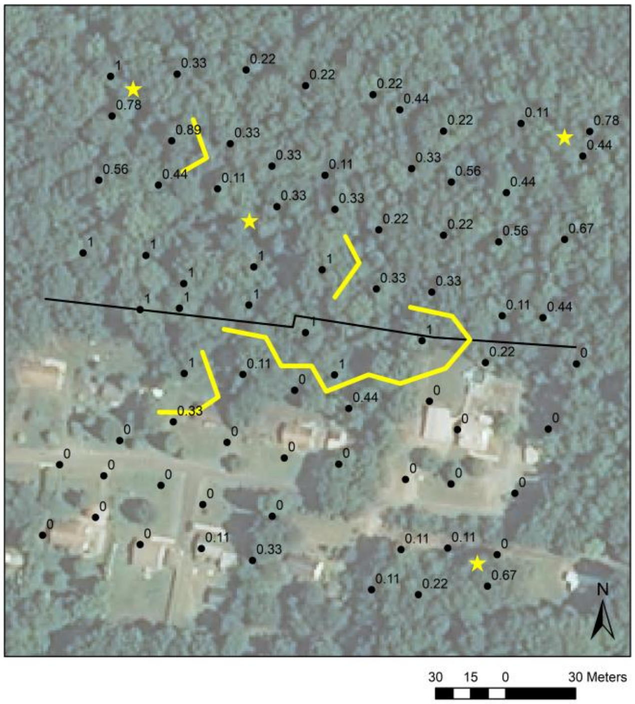

Figure S7. Rural_small_Coarse woody debris cover

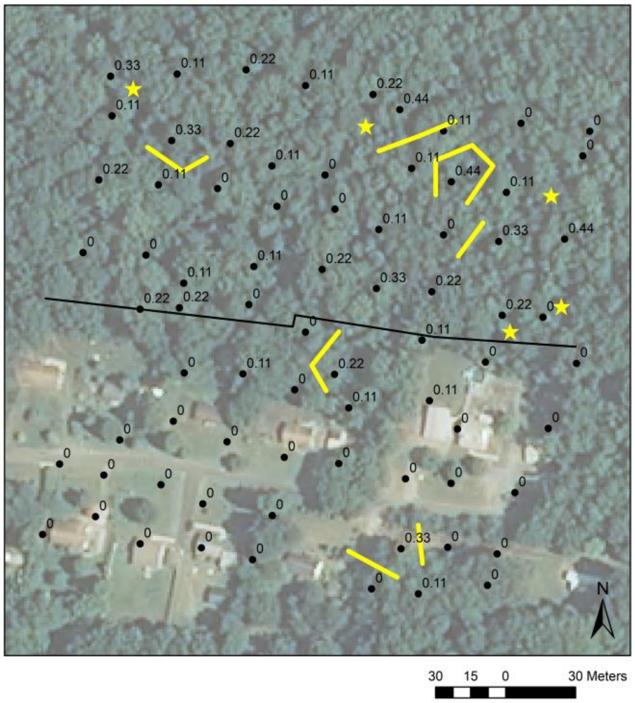

Figure S8. Rural_small_All environmental variables

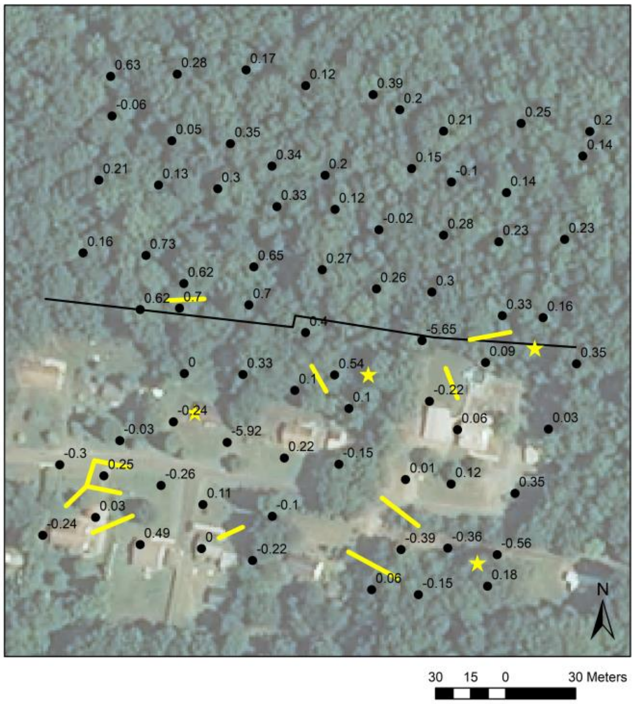

Figure S9. Rural_small_All species

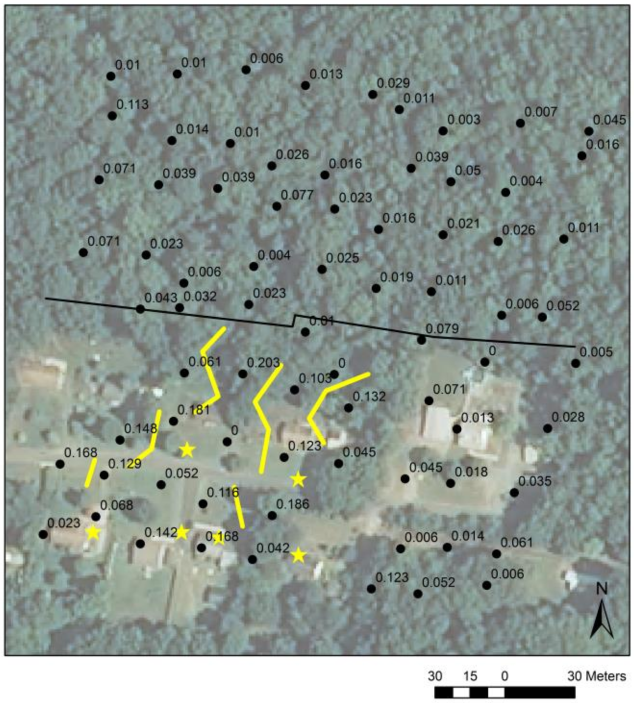

Figure S10. Rural_small_Total evenness

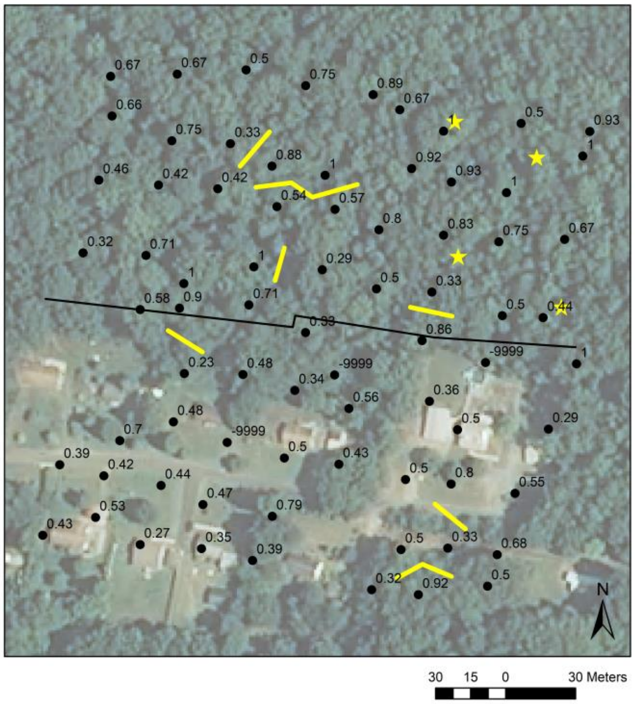

Figure S11. Rural_small_Amara aenea

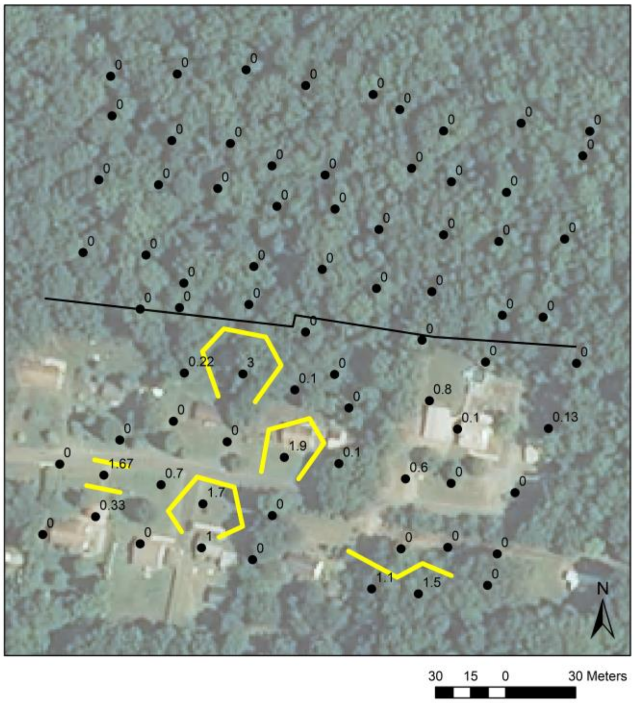

Figure S12. Rural_small_Harpalus pensylvanicus

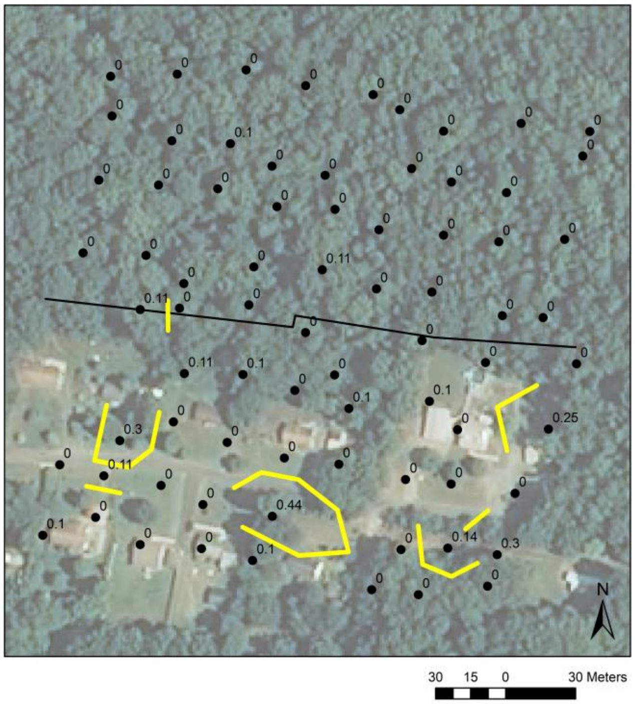

Figure S13. Rural_small_Sphaeroderus stenostomus lecontei

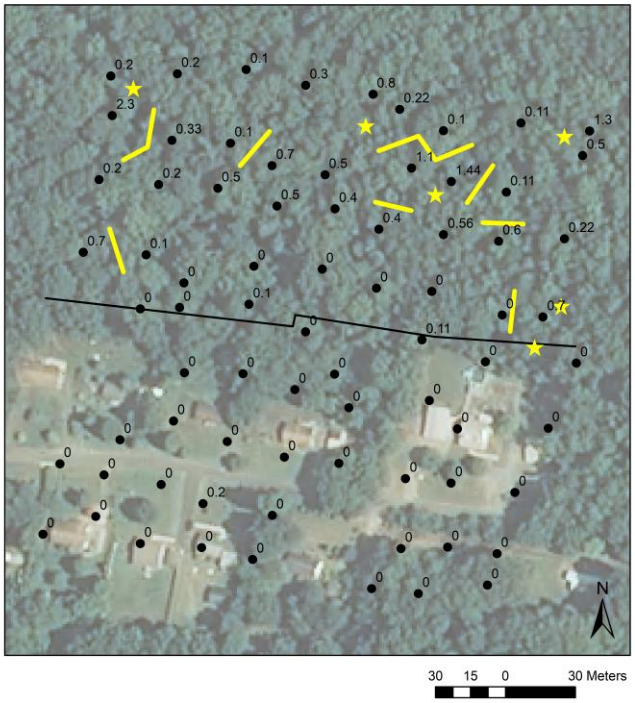

Figure S14. Rural_small_Trichotichnus fulgens

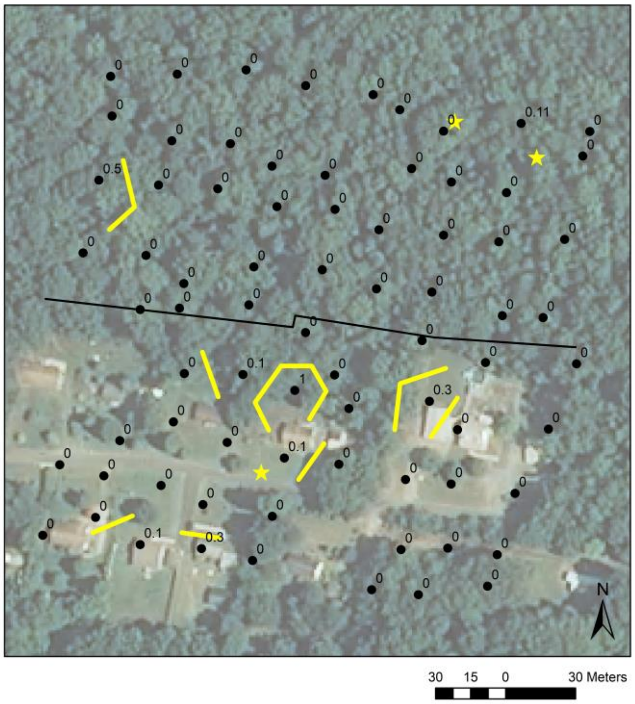

Figure S15. Rural_large_Slope

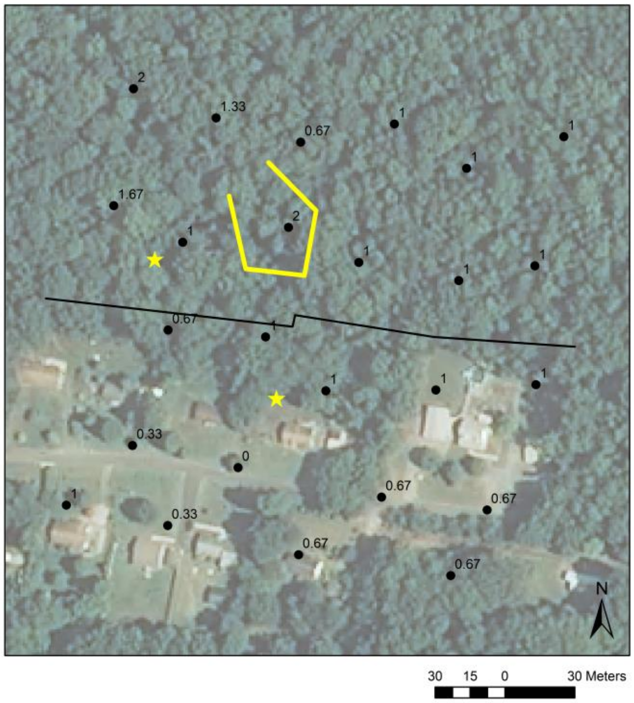

Figure S16. Rural_large_Temperature

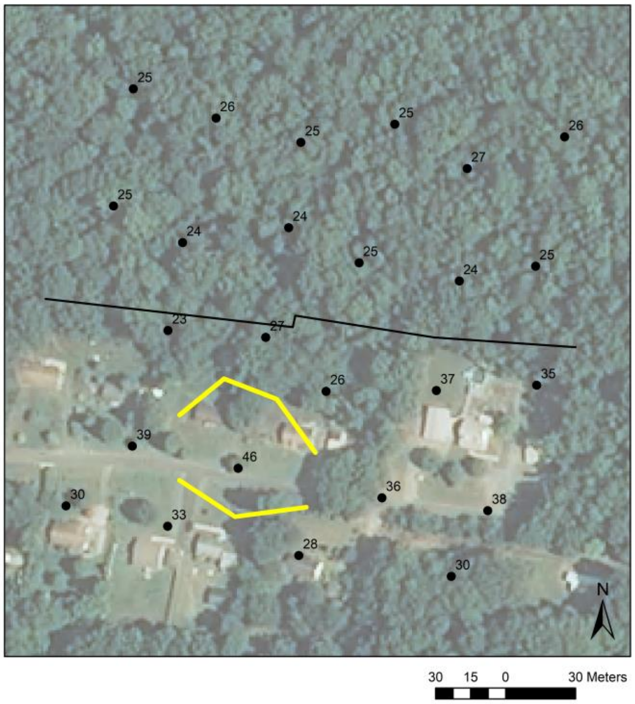

Figure S17. Rural_large_Bare ground cover

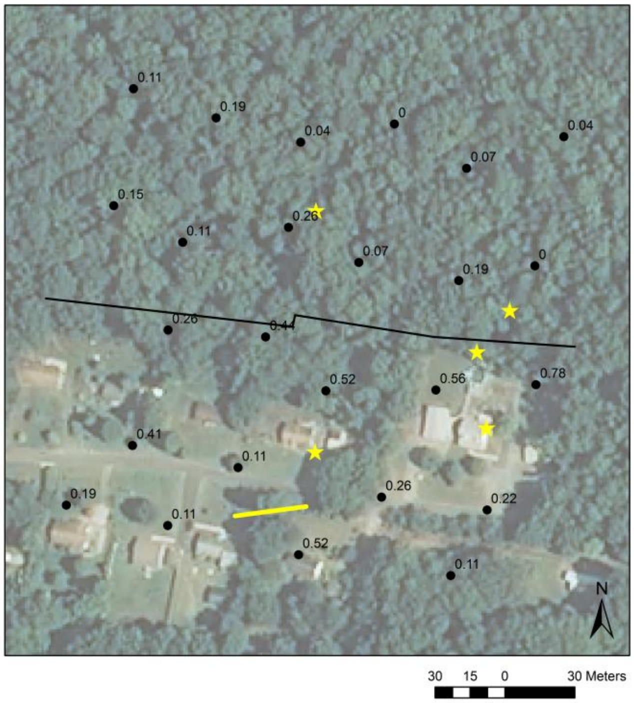

Figure S18. Rural_large_Forb cover

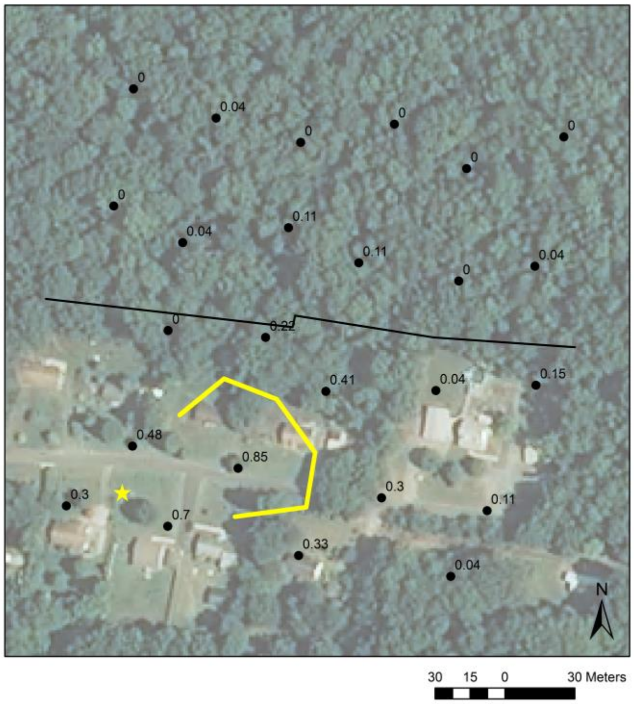

Figure S19. Rural_large_Leaf litter depth

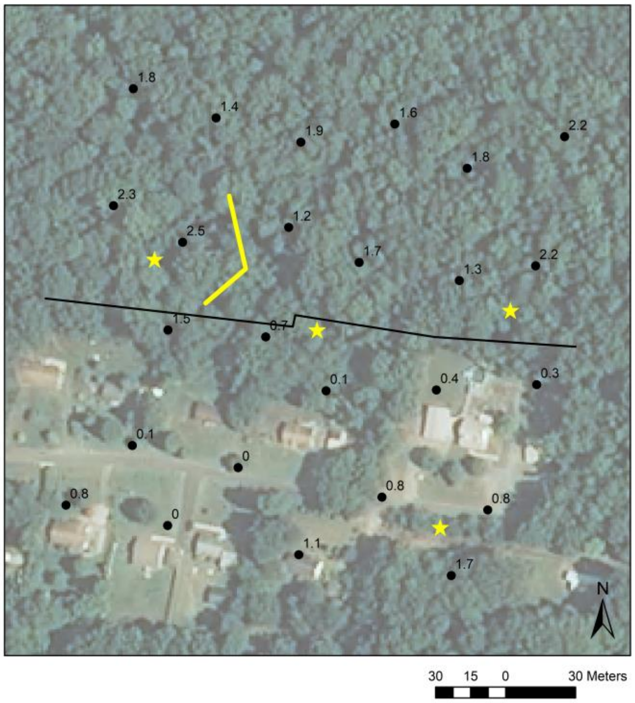

Figure S20. Rural_large_Generalist richness

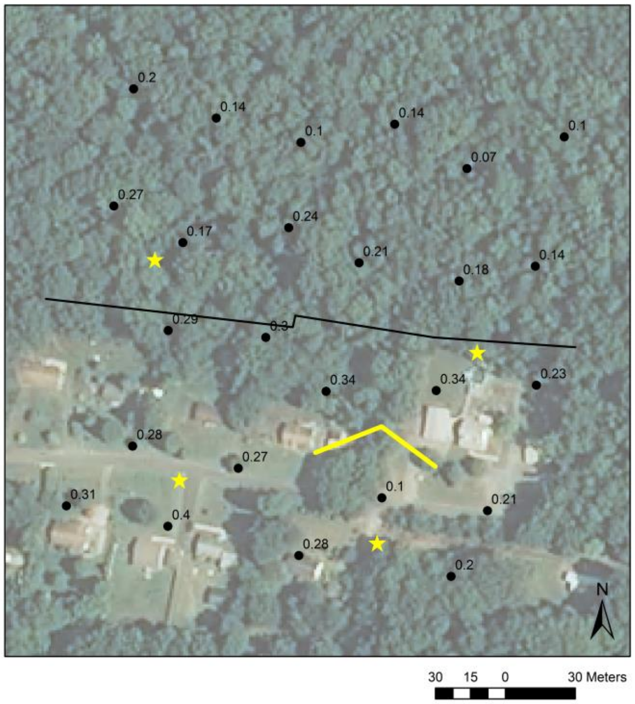

Figure S21. Rural_large_Total evenness

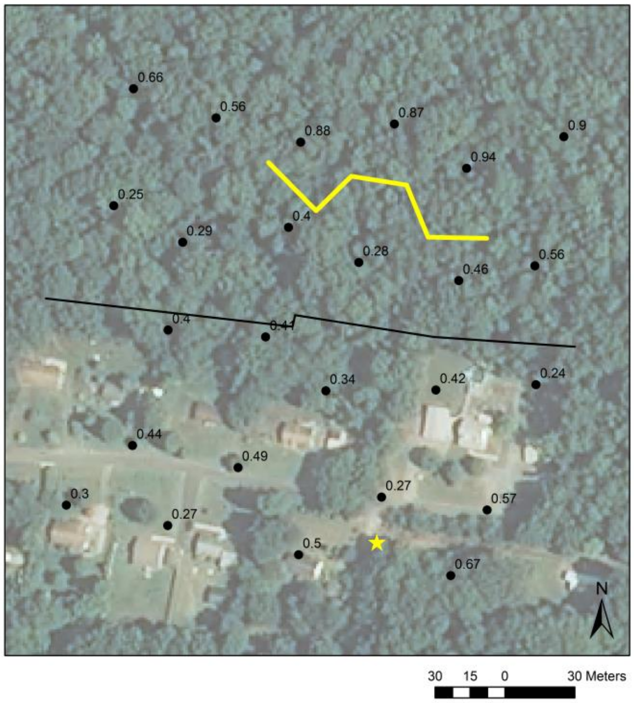

Figure S22. Rural_large_All species

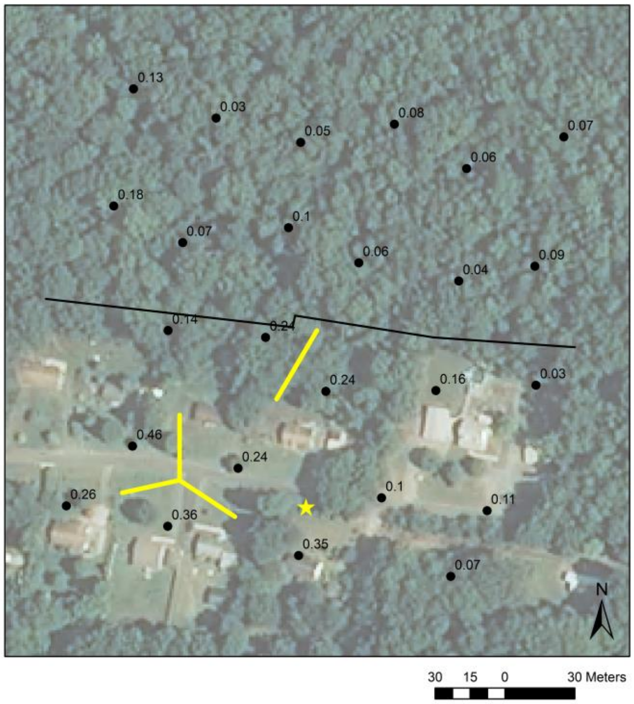

Figure S23. Rural_large_All species weighted

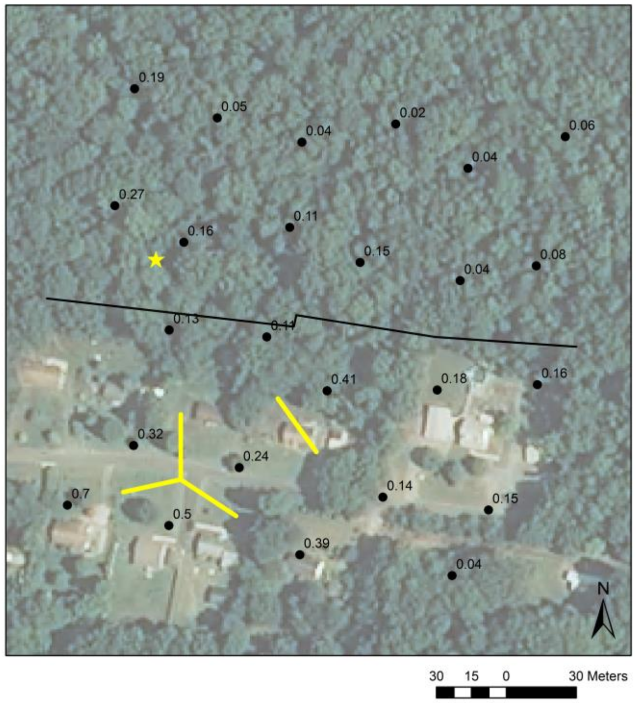

Figure S24. Rural_large_Generalist species

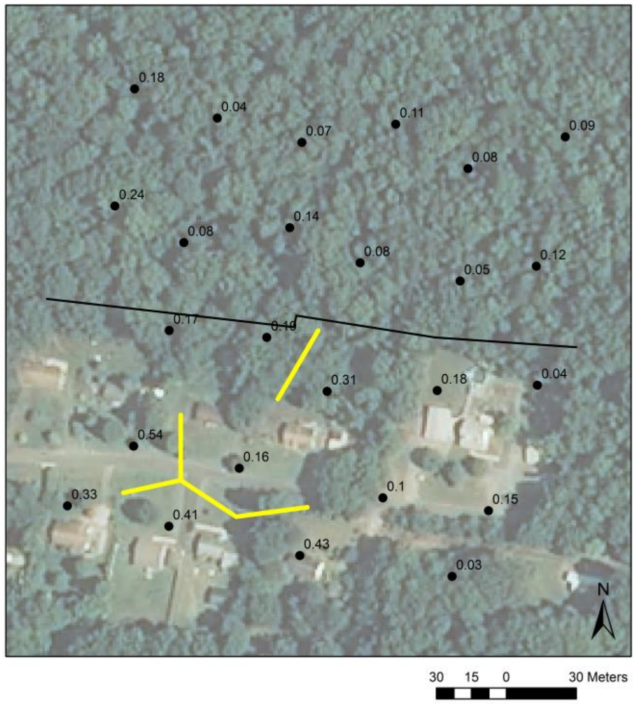

Figure S25. Rural_large_Anisodactylus dulcicollis

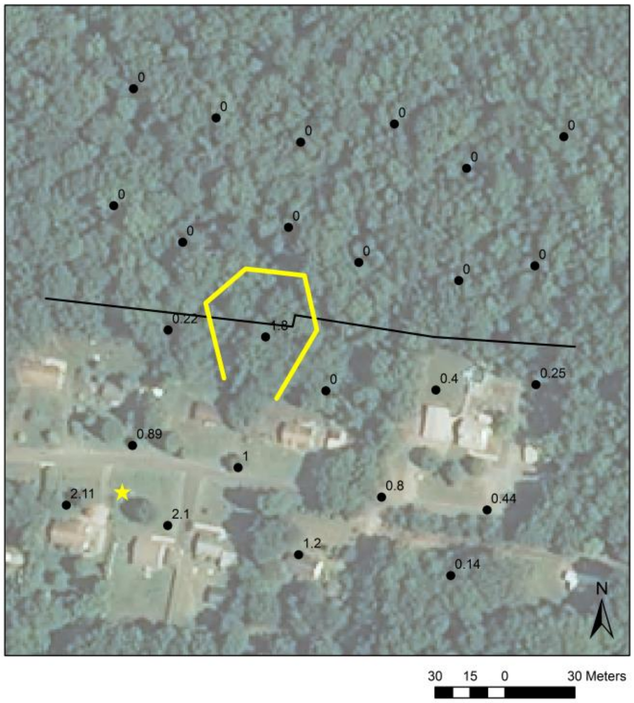

Figure S26. Rural_large_Anisodactylus opaculus

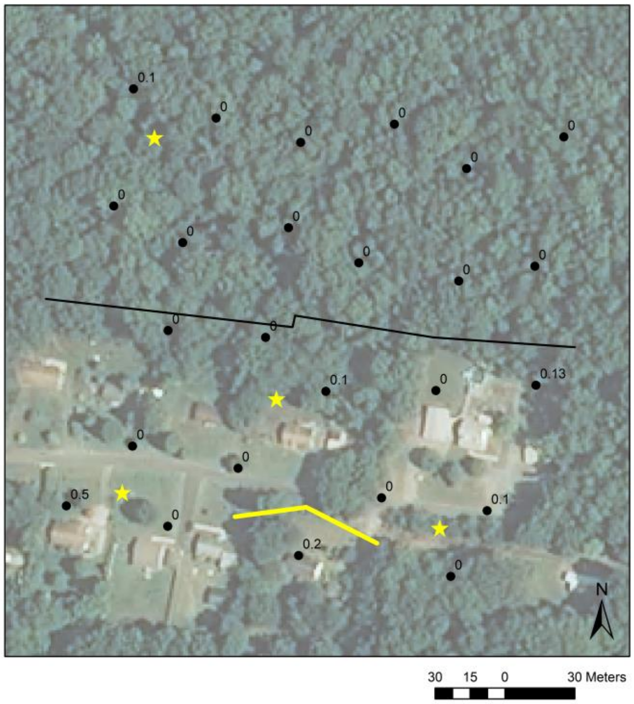

Figure S27. Rural_large_Anisodactylus rusticus

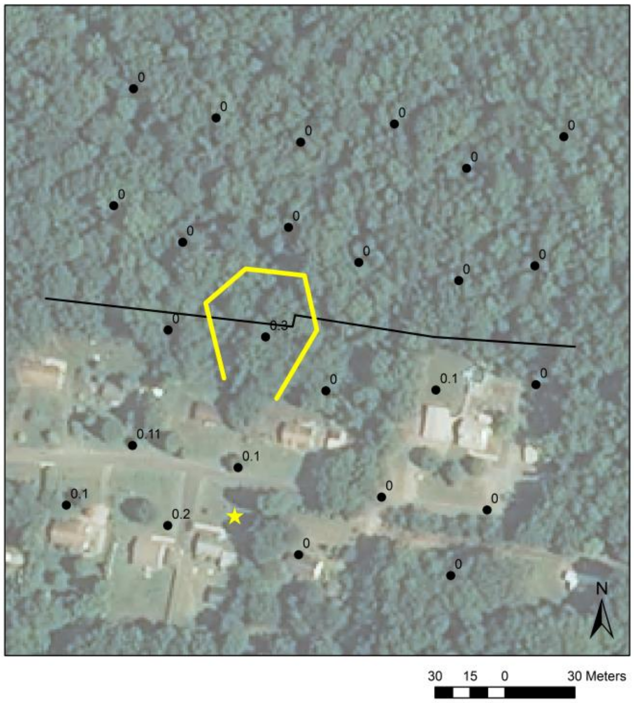

Figure S28. Rural_large_Chlaenius prasinus

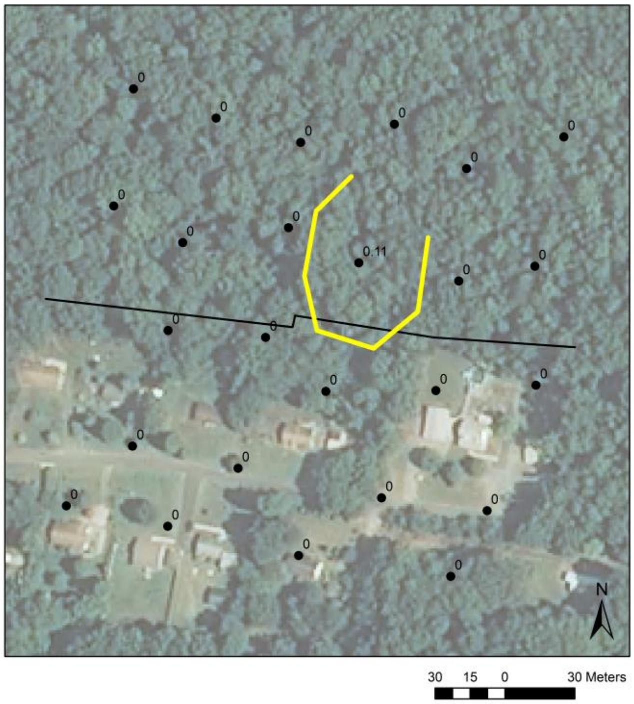

Figure S29. Rural_large_Galerita janus

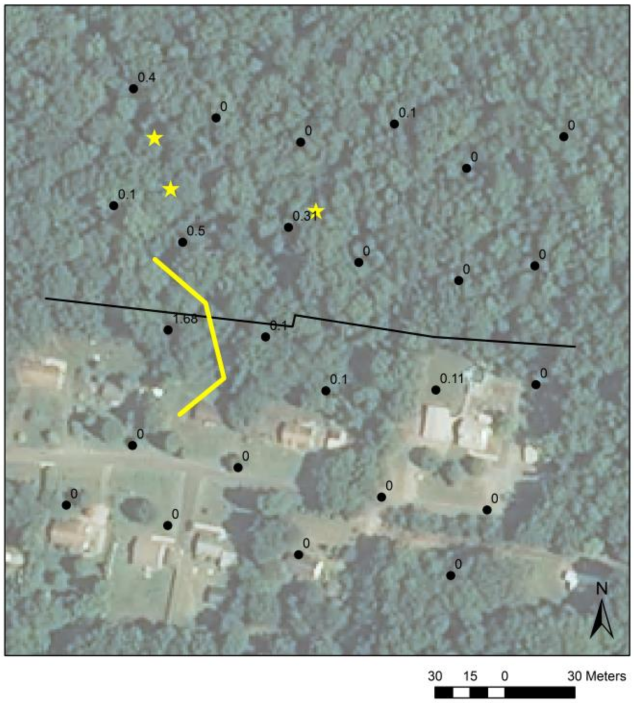

Figure S30. Rural_large_Lebia vittata

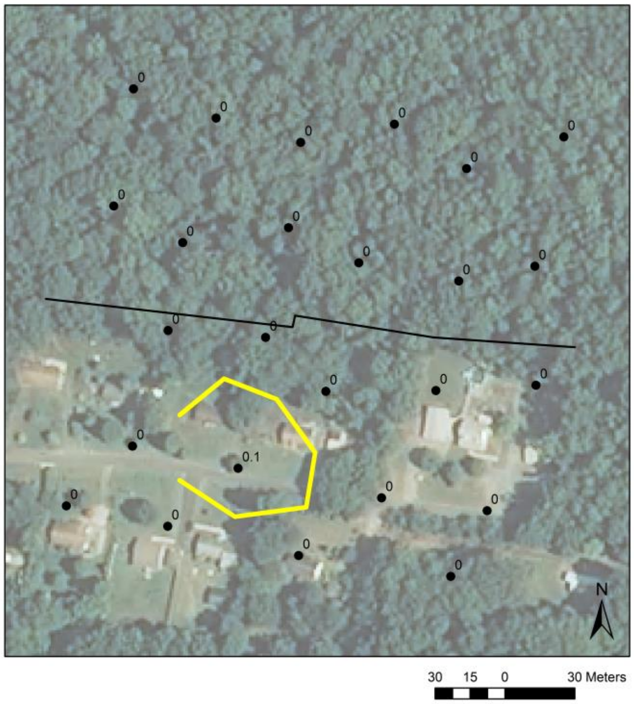

Figure S31. Suburban_small_Slope

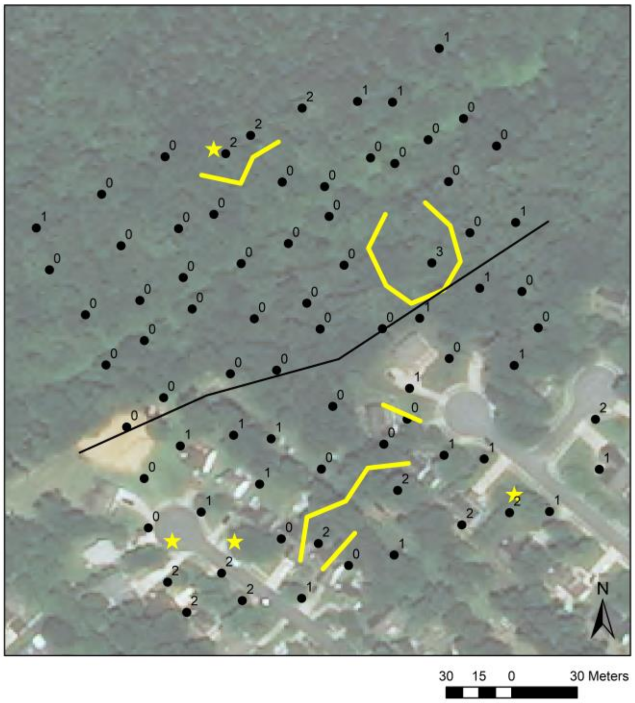

Figure S32. Suburban_small_Leaf litter depth

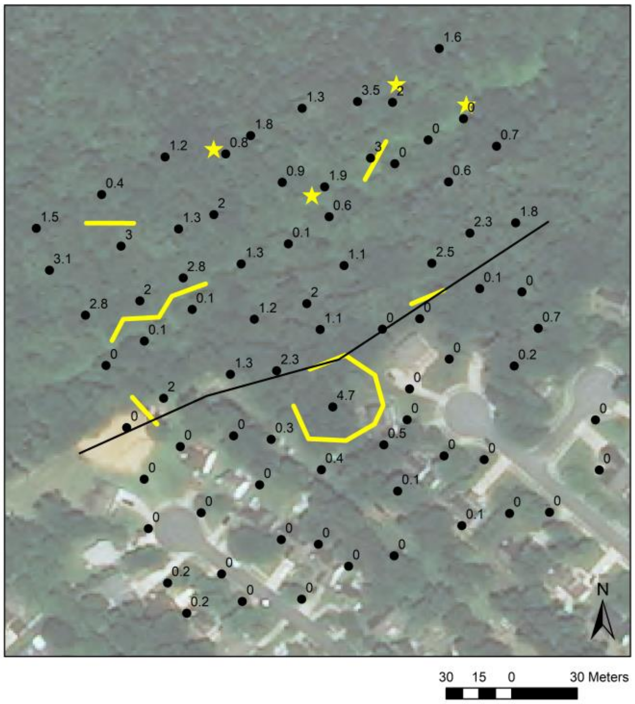

Figure S33. Suburban_small_Grass cover

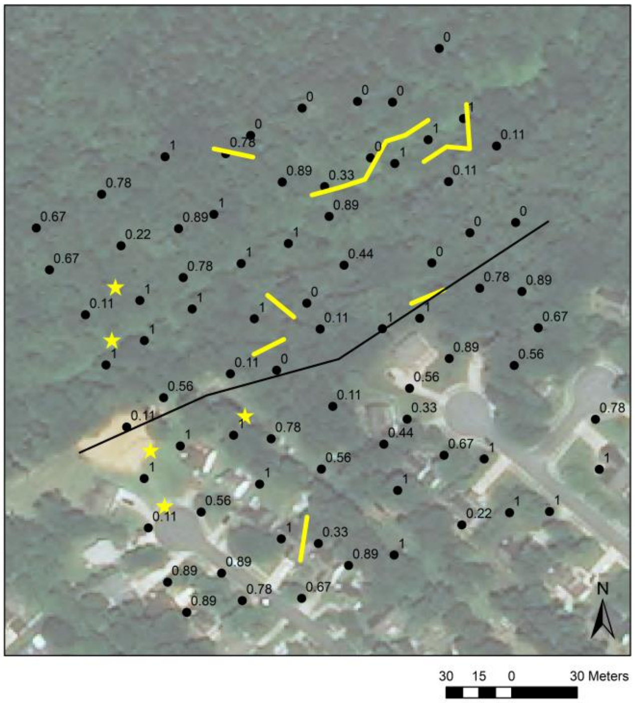

Figure S34. Suburban_small_Creeping forb cover

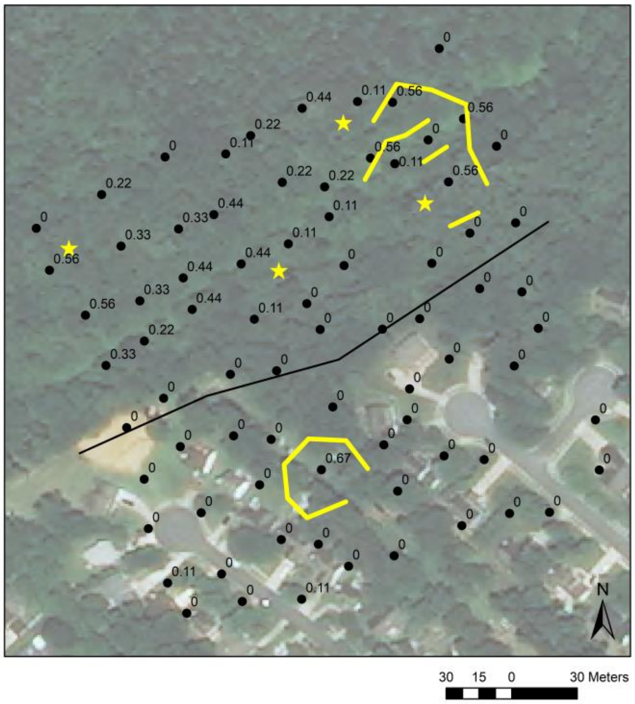

Figure S35. Suburban_small_Shrub cover

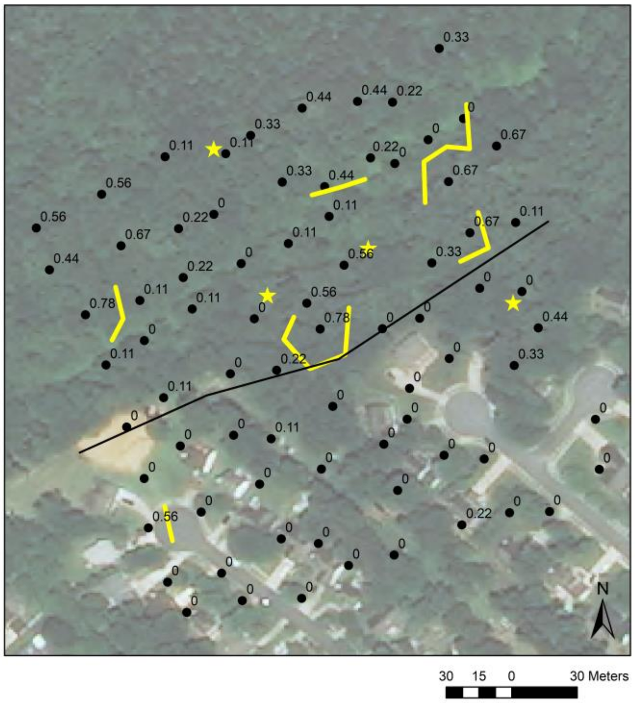

Figure S36. Suburban_small_Moss cover

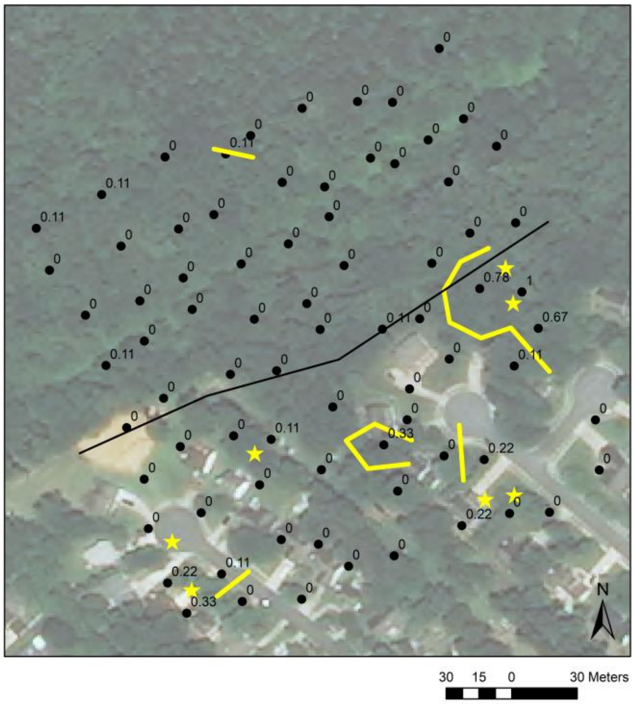

Figure S37. Suburban_small_Total evenness

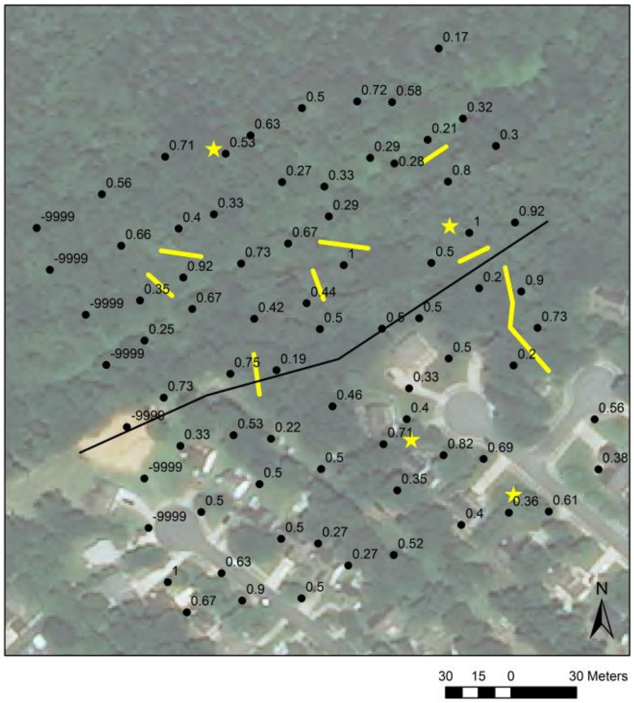

Figure S38. Suburban_small_Generalist evenness

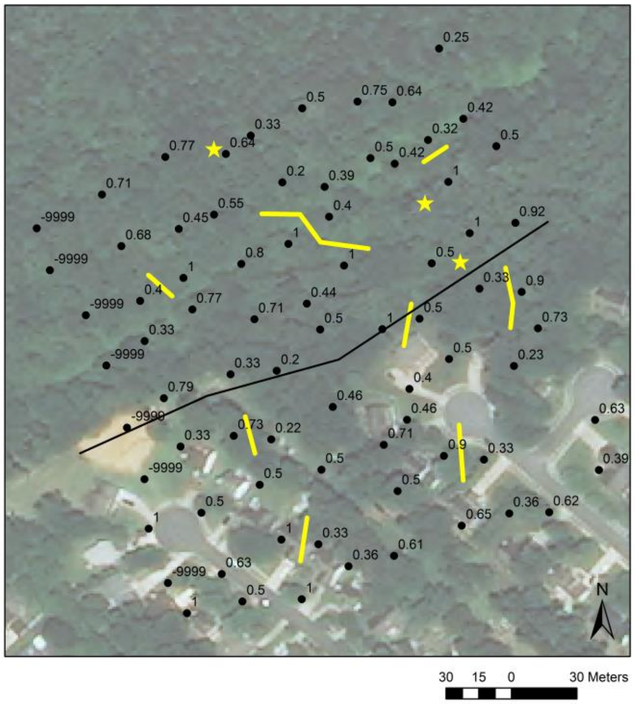

Figure S39. Suburban_small_Open species

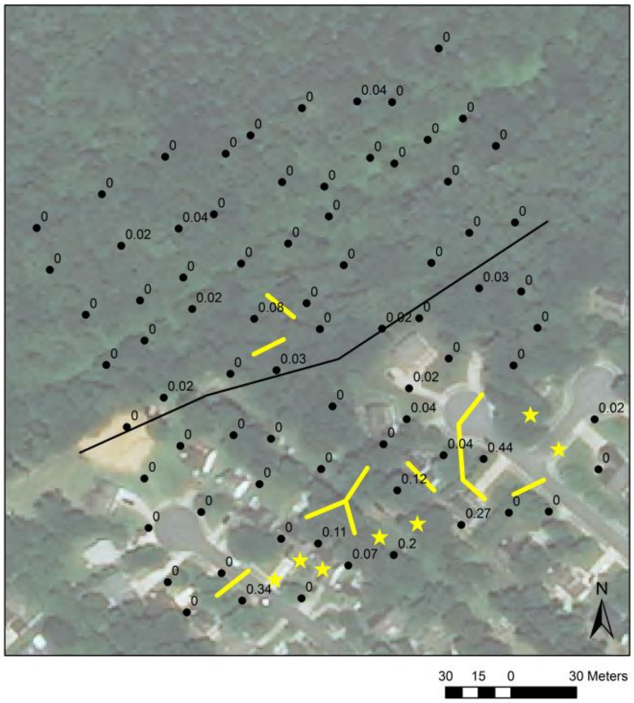

Figure S40. Suburban_small_Generalist species weighted

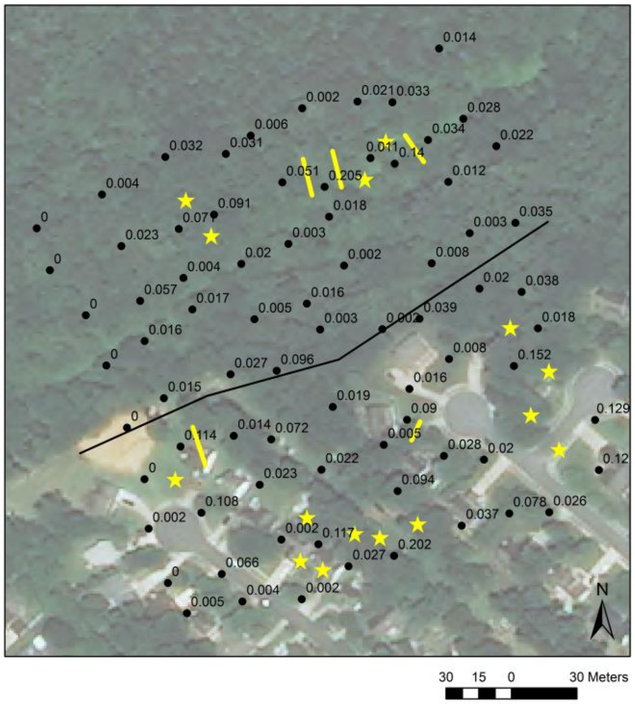

Figure S41. Suburban_small_Amara aenea

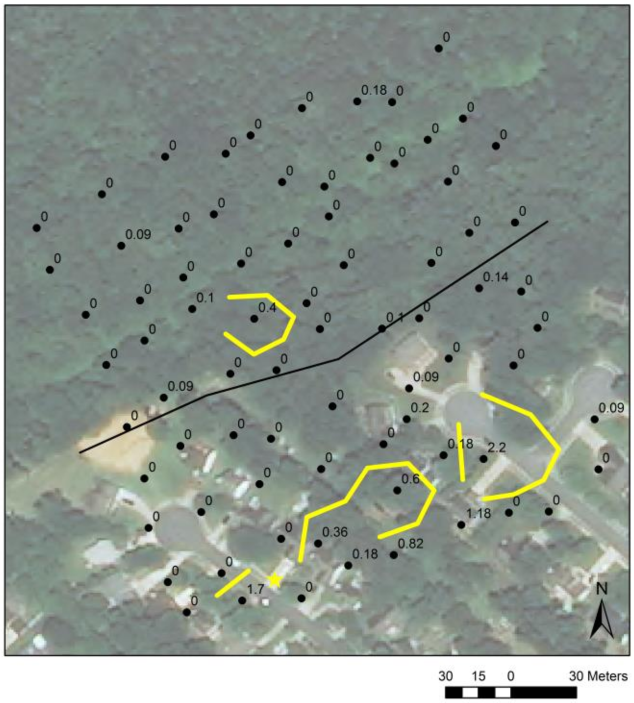

Figure S42. Suburban_small_Anisodactylus dulcicollis

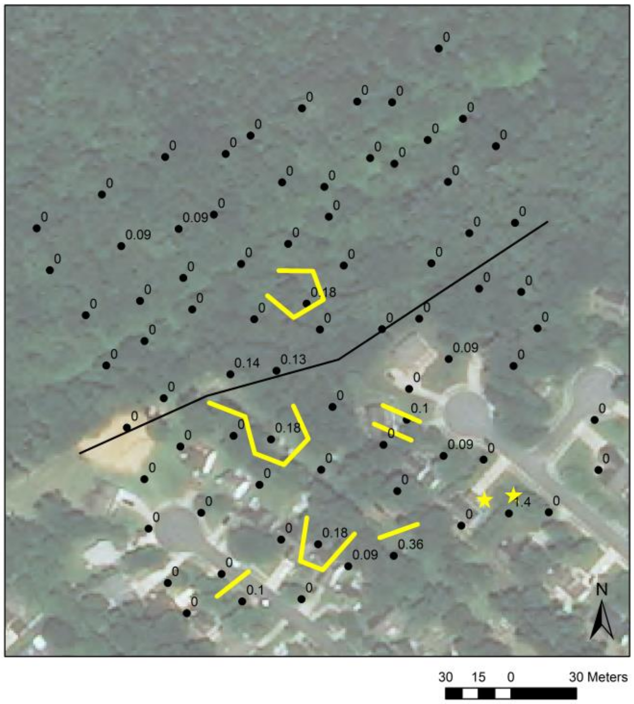

Figure S43. Suburban_small_Dicaelus dilatatus

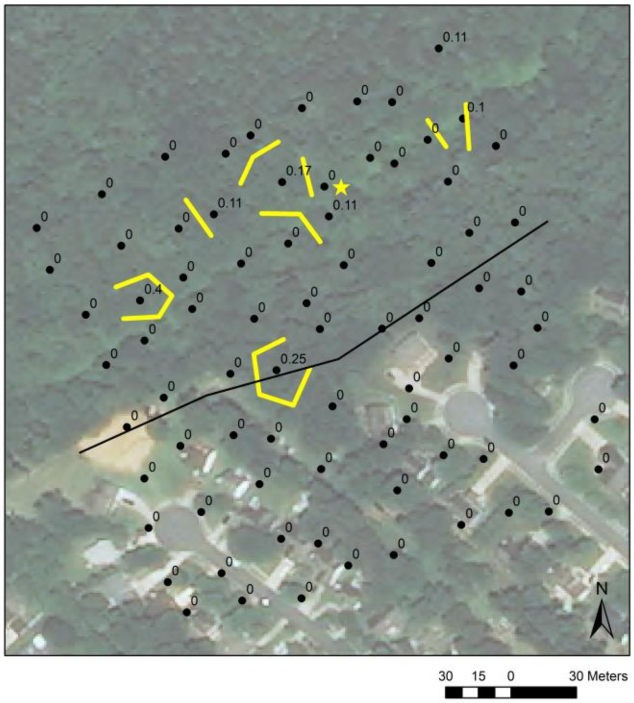

Figure S44. Suburban_small_Pterostichus sculptus

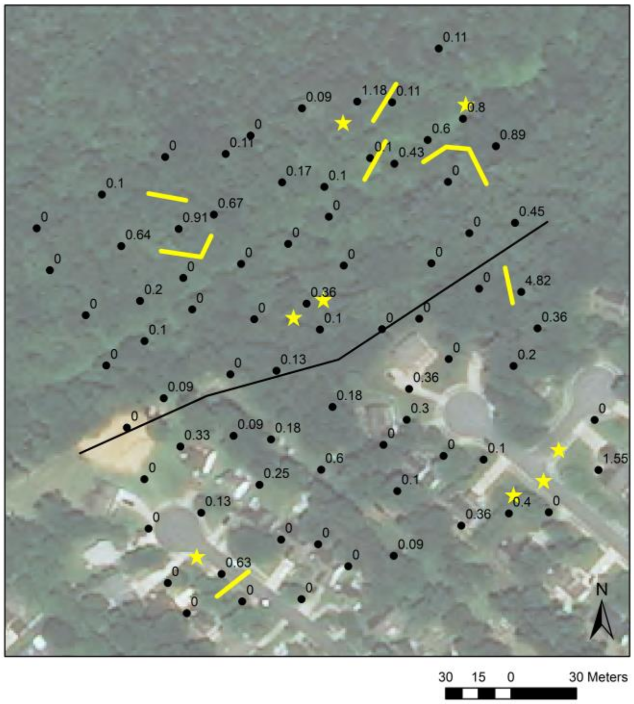

Figure S45. Suburban_small_Scarites subterraneus

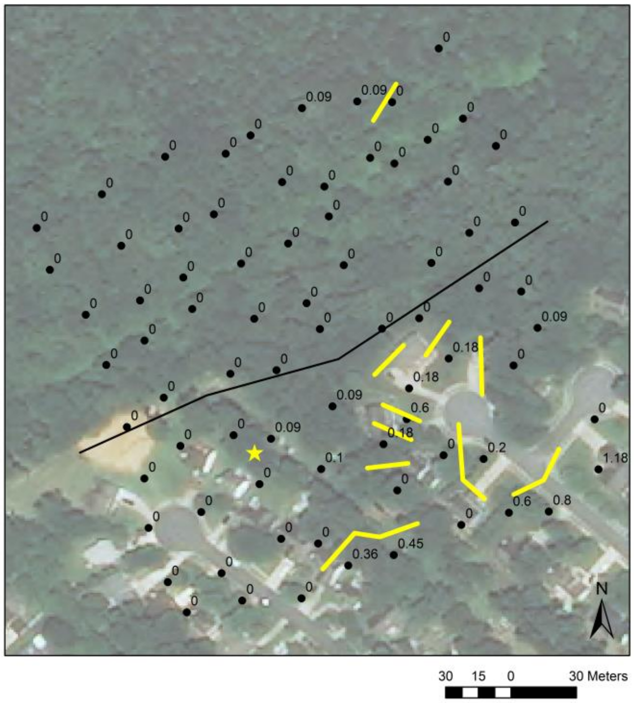

Figure S46. Suburban_large_Microrelief

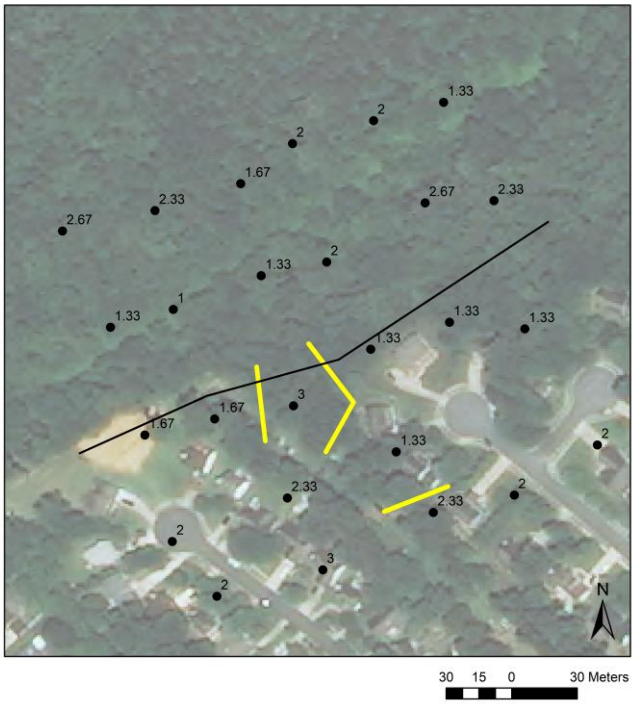

Figure S47. Suburban_large_Canopy cover

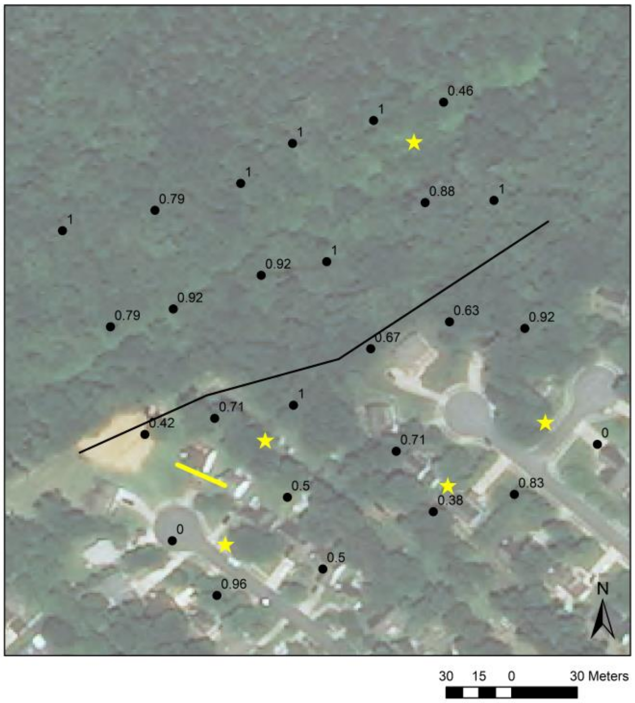

Figure S48. Suburban_large_Vine cover

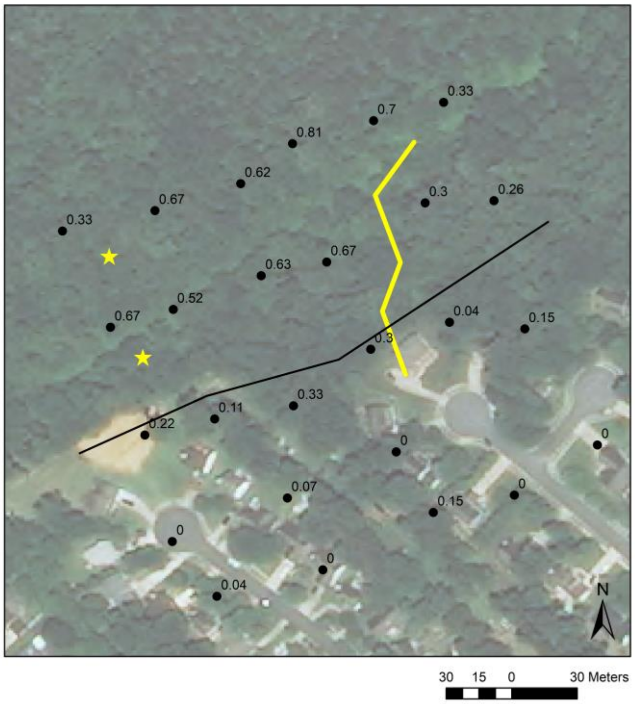

Figure S49. Suburban_large_Forest richness

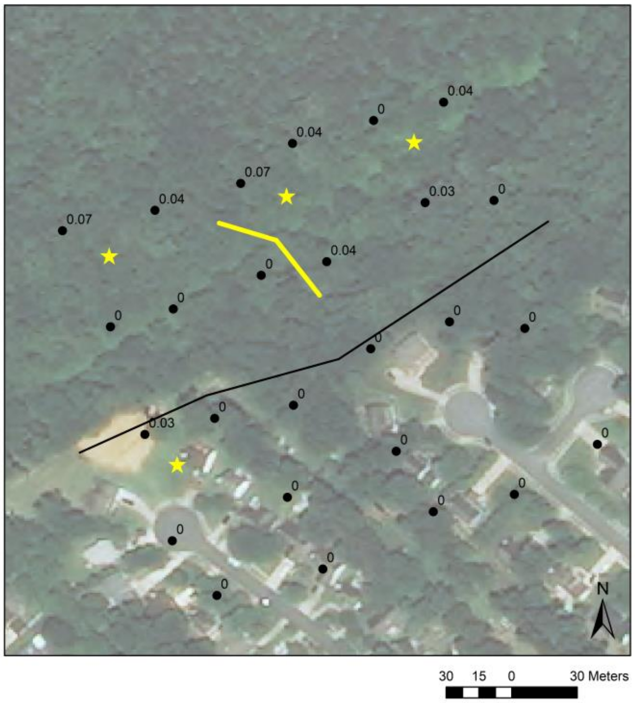

Figure S50. Suburban_large_Generalist abundance

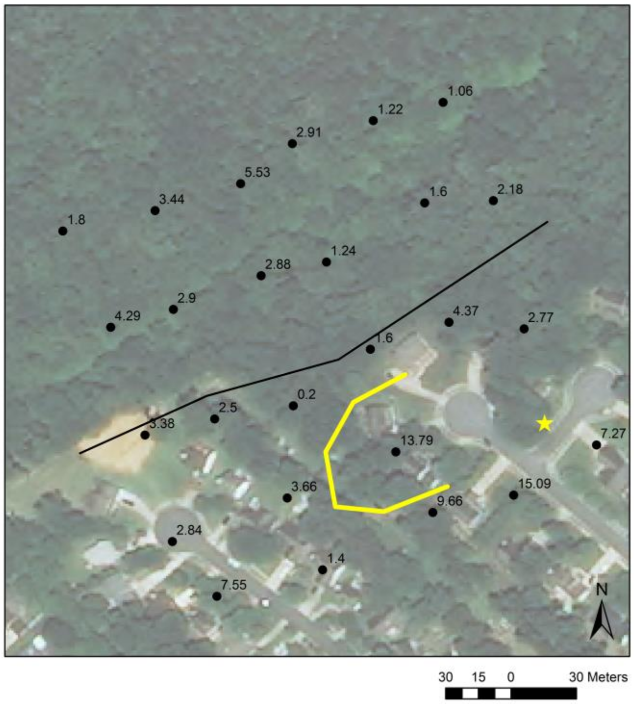

Figure S51. Suburban_large_All species

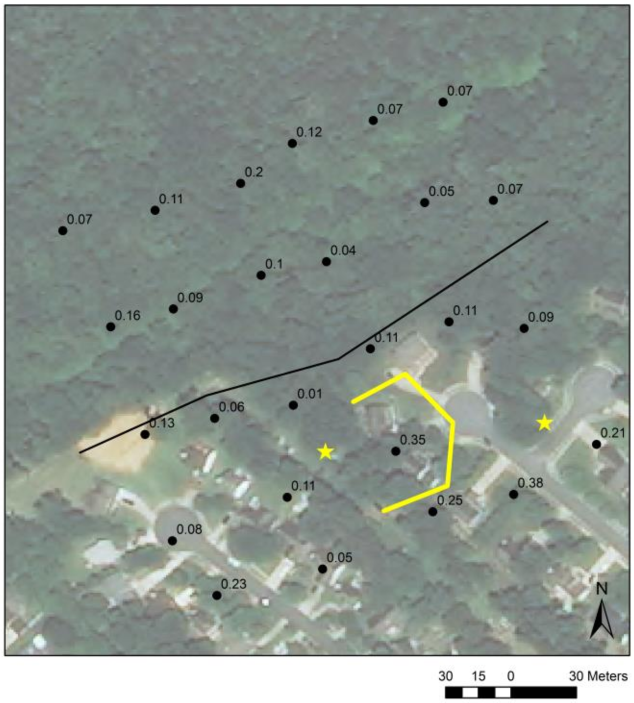

Figure S52. Suburban_large_All species weighted

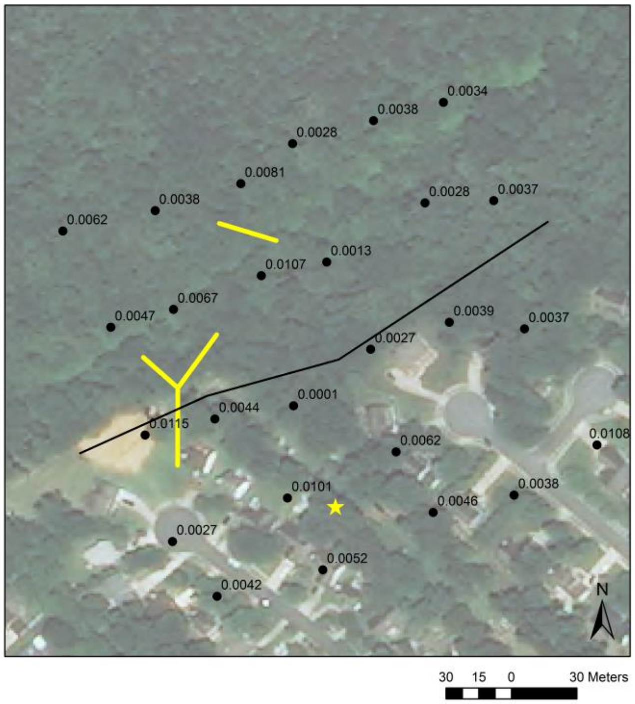

Figure S53. Suburban_large_Open species

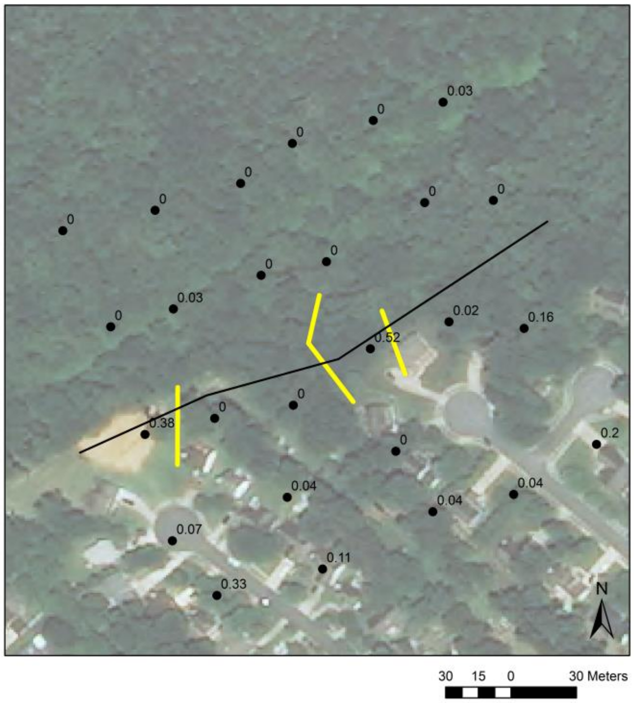

Figure S54. Suburban_large_Open species weighted

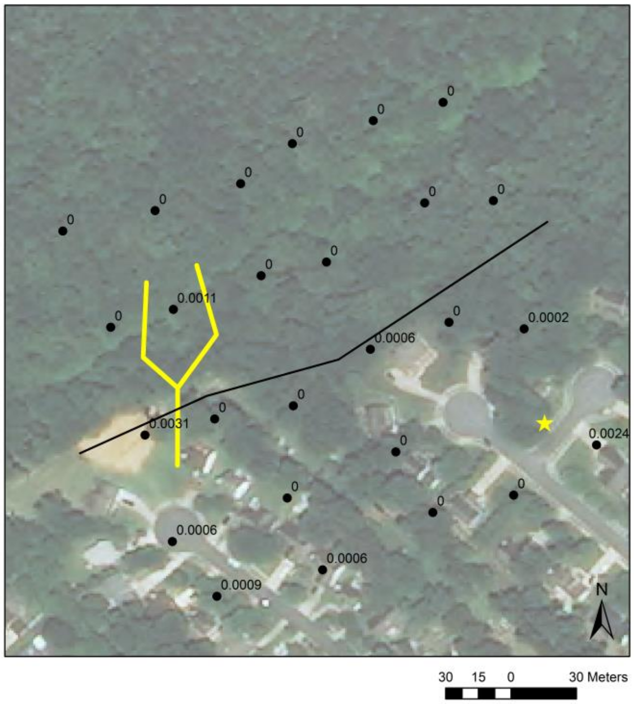

Figure S55. Suburban_large_Generalist species

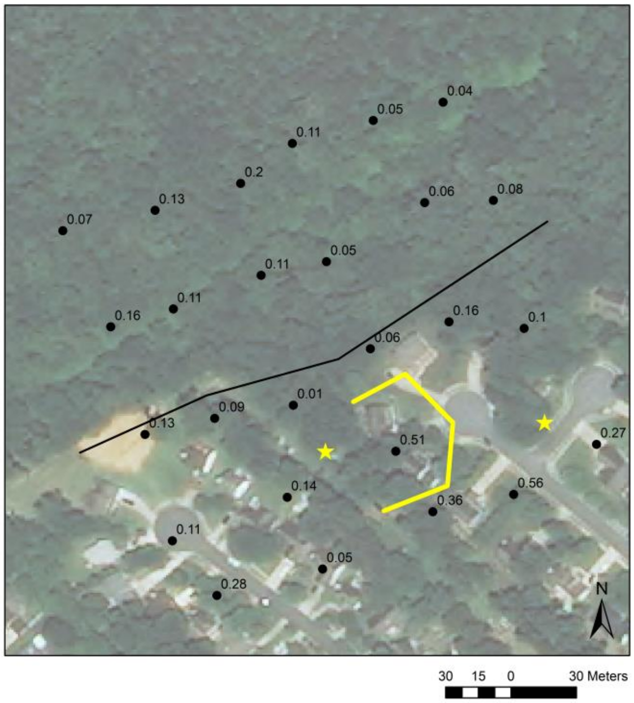

Figure S56. Suburban_large_Cyclotrachelus sigillatus

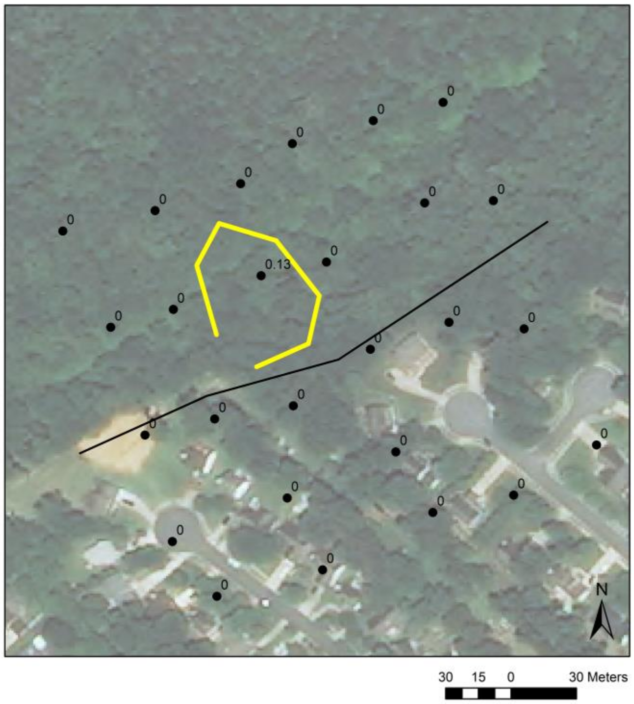

Figure S57. Suburban_large_Olisthopus parmatus

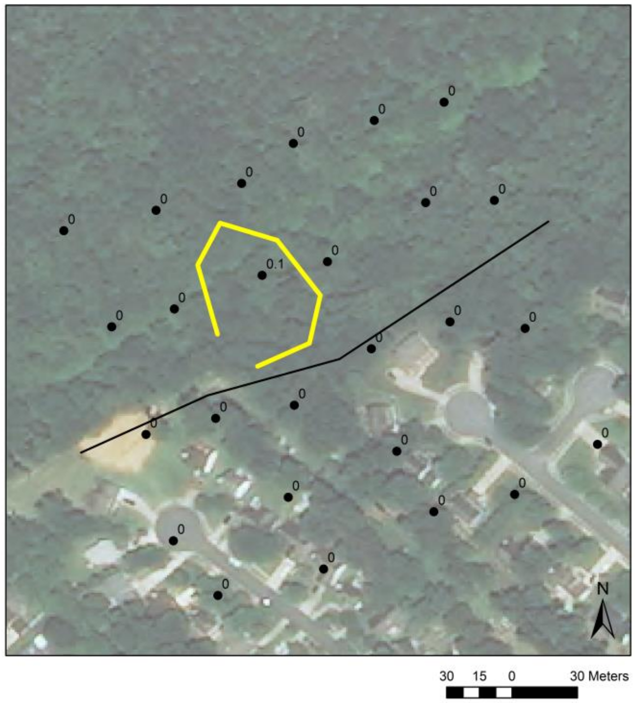

Figure S58. Suburban_large_Scarites quadriceps

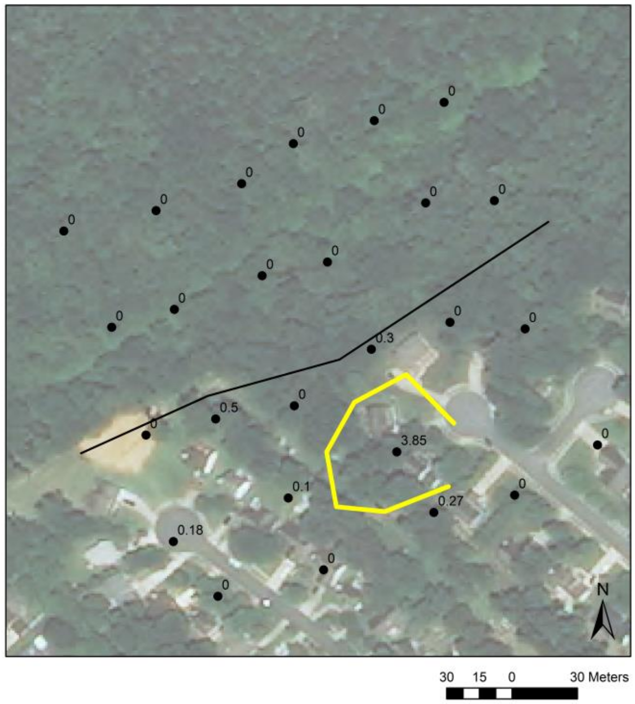

Figure S59. Suburban_large_Scarites subterraneus

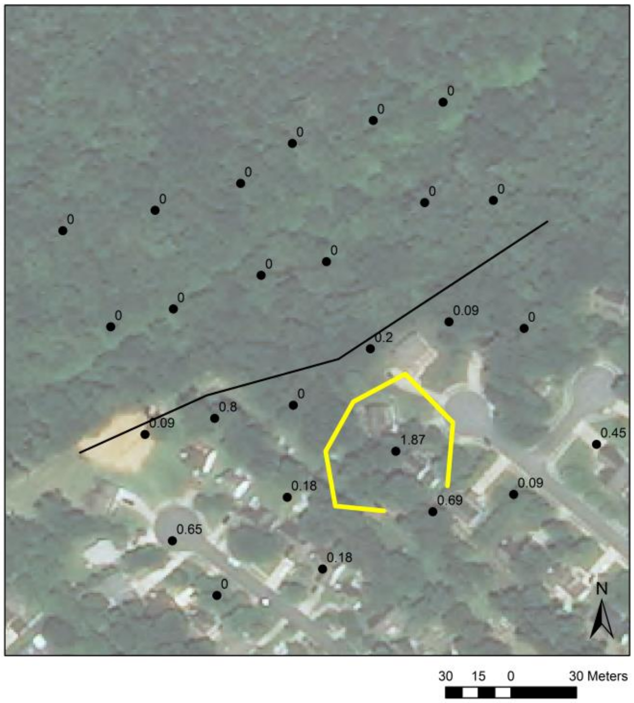

Figure S60. Suburban_large_Stenolophus rotundatus

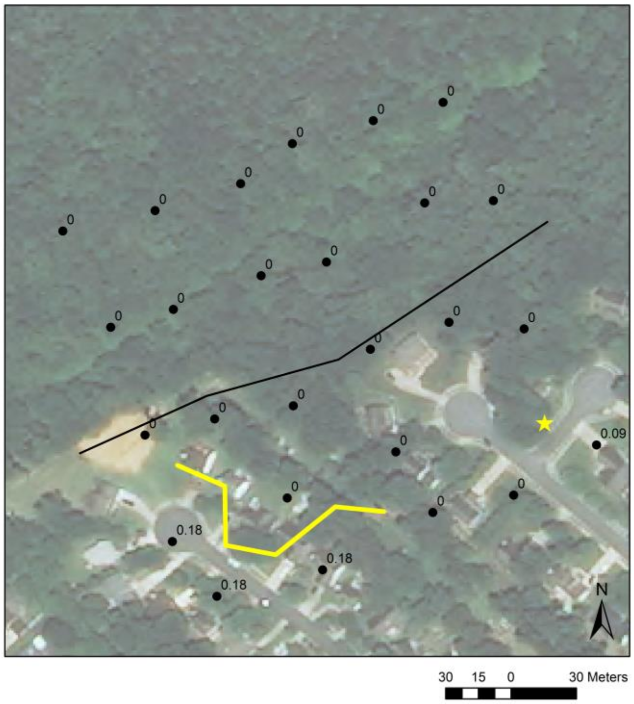

Figure S61. Urban_small_Temperature

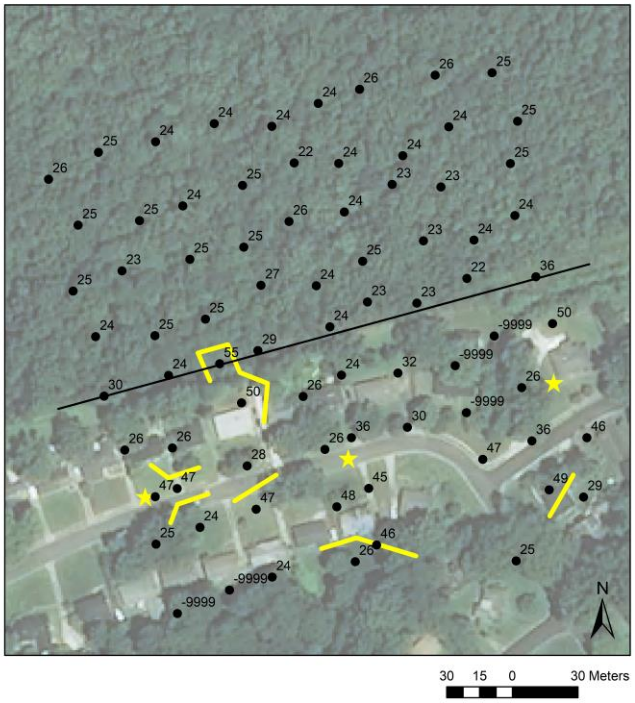

Figure S62. Urban_small_Humidity

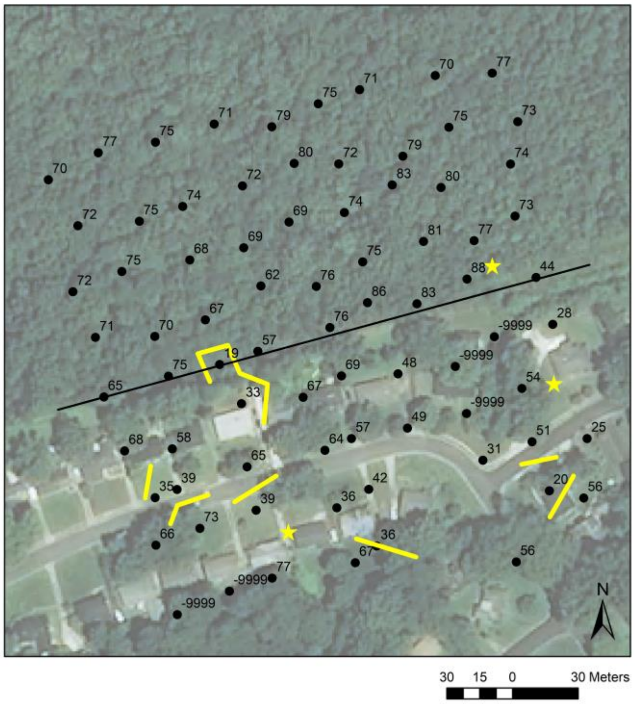

Figure S63. Urban_small_Canopy cover

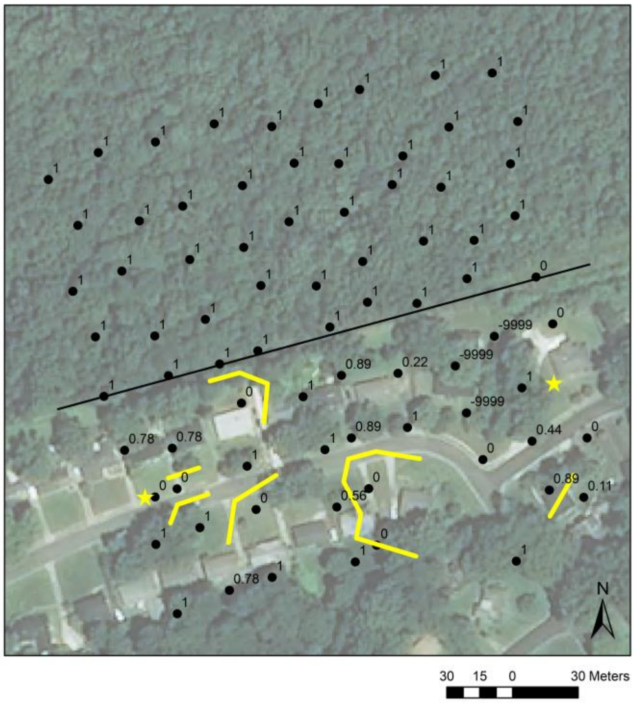

Figure S64. Urban_small_Forb cover

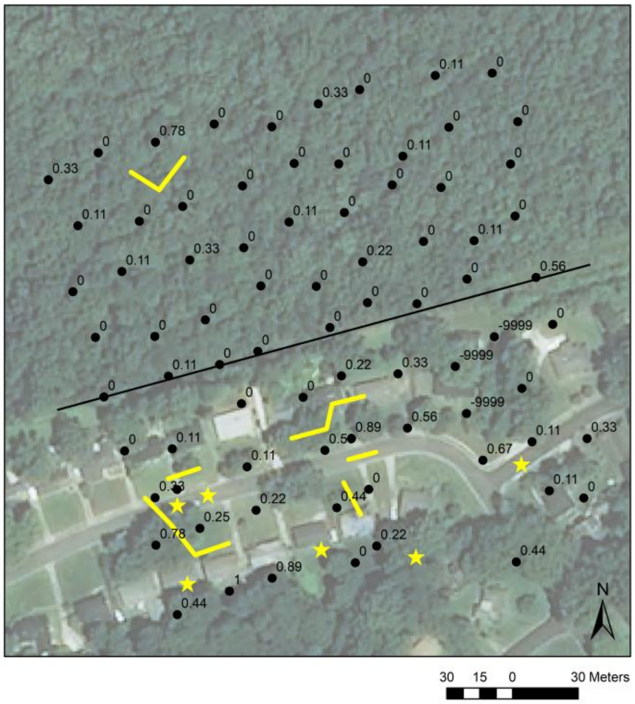

Figure S65. Urban_small_Grass cover

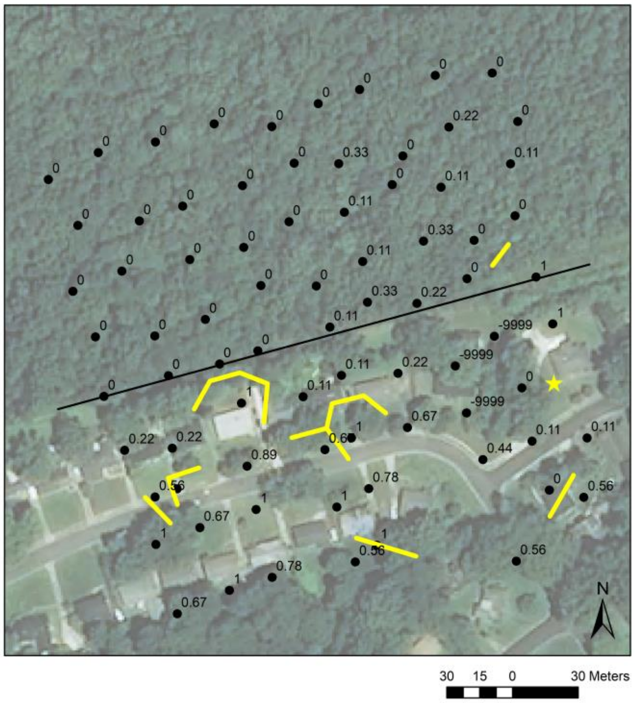

Figure S66. Urban_small_Shrub cover

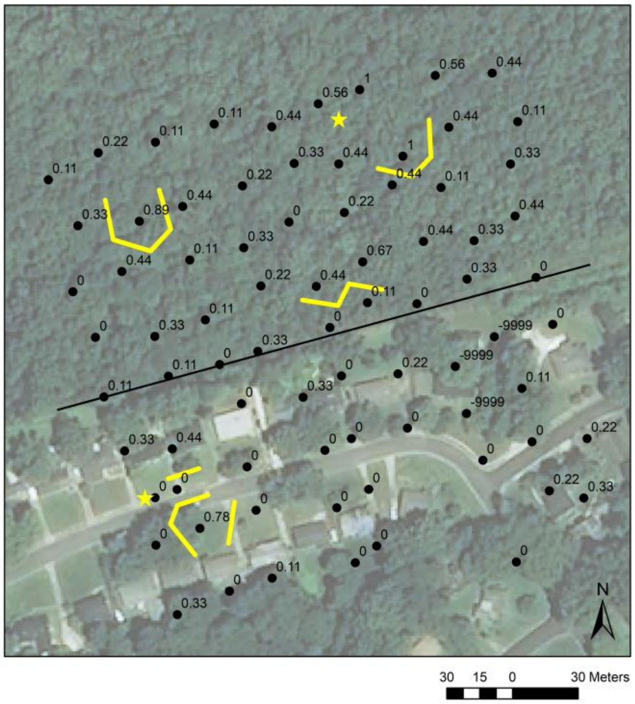

Figure S67. Urban_small_Vine cover

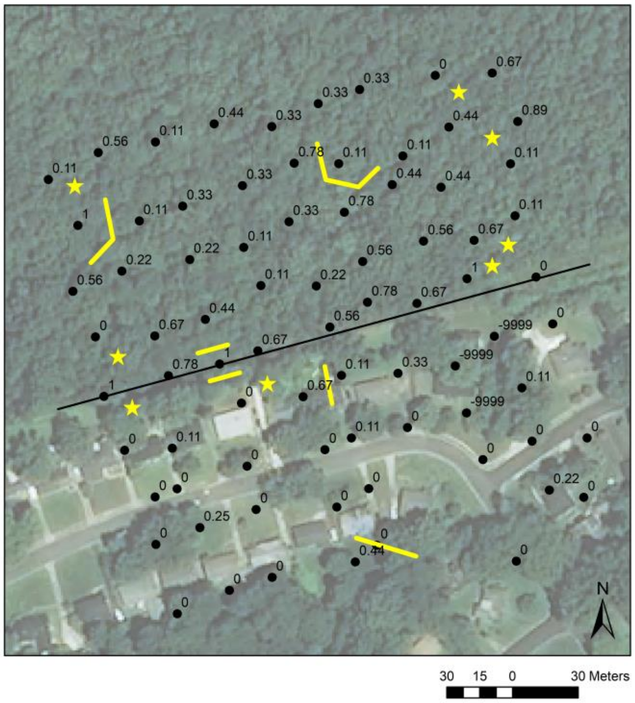

Figure S68. Urban_small_Bare ground cover

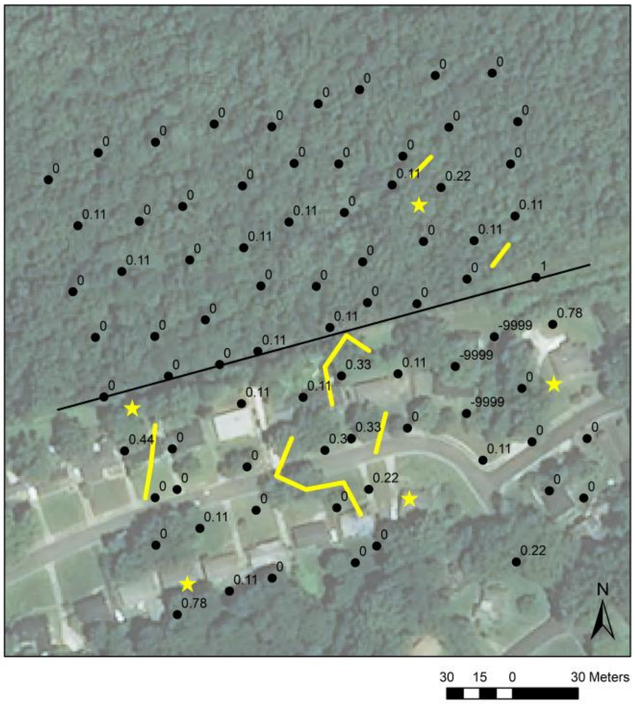

Figure S69. Urban_small_Impervious surface cover

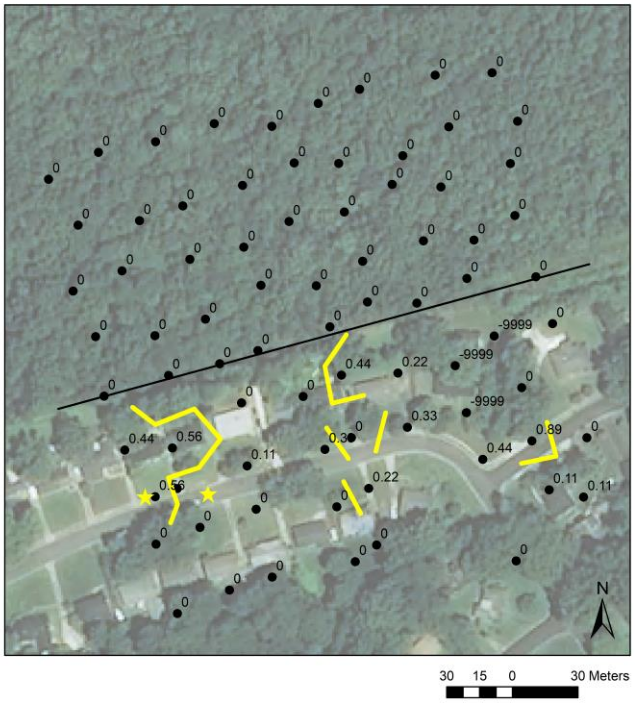

Figure S70. Urban_small_All environmental variables

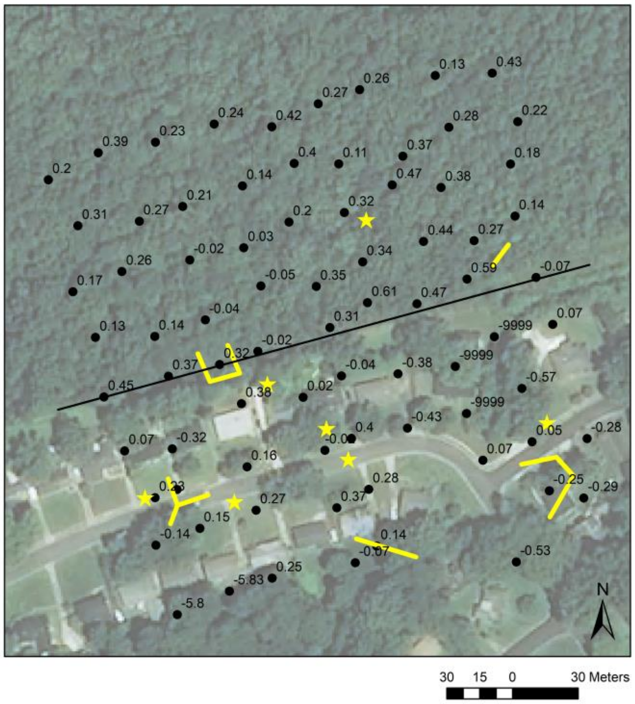

Figure S71. Urban_small_Total abundance

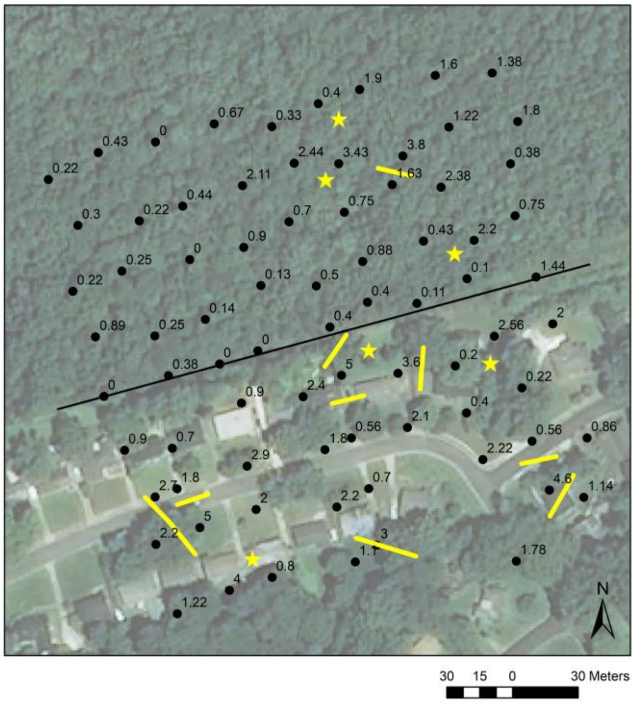

Figure S72. Urban_small_Total evenness

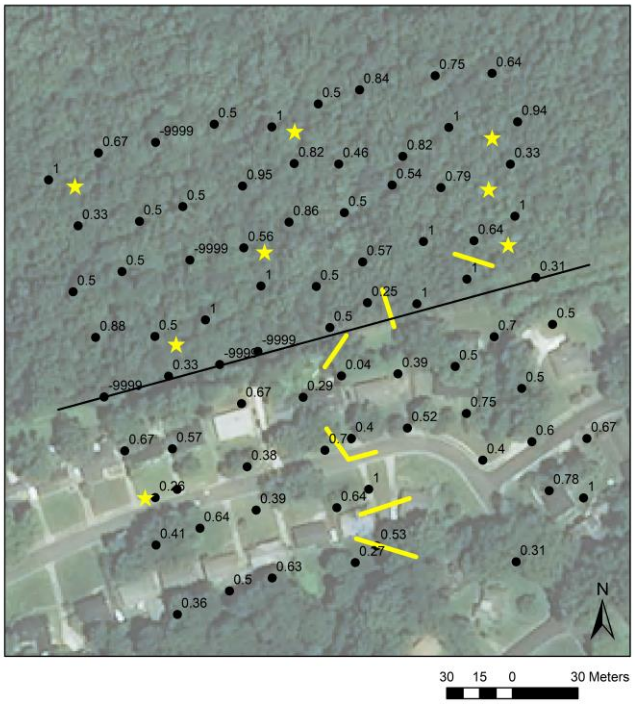

Figure S73. Urban_small_Forest abundance

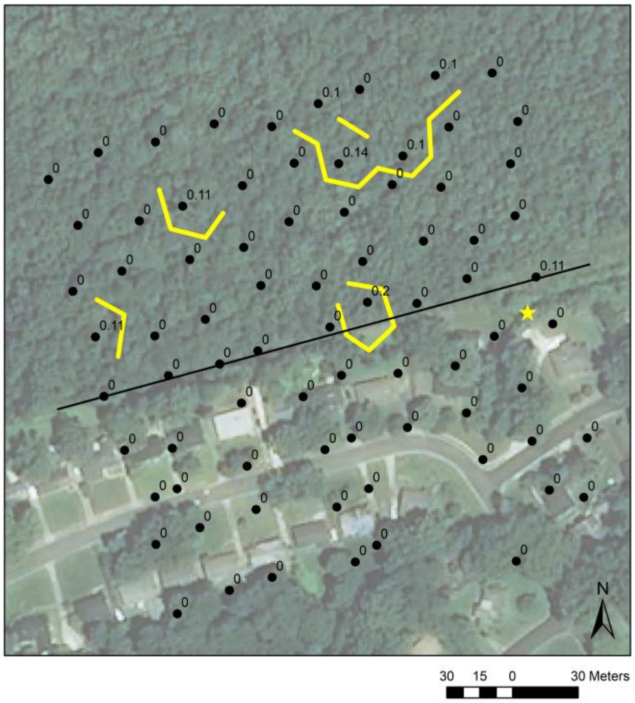

Figure S74. Urban_small_Forest richness

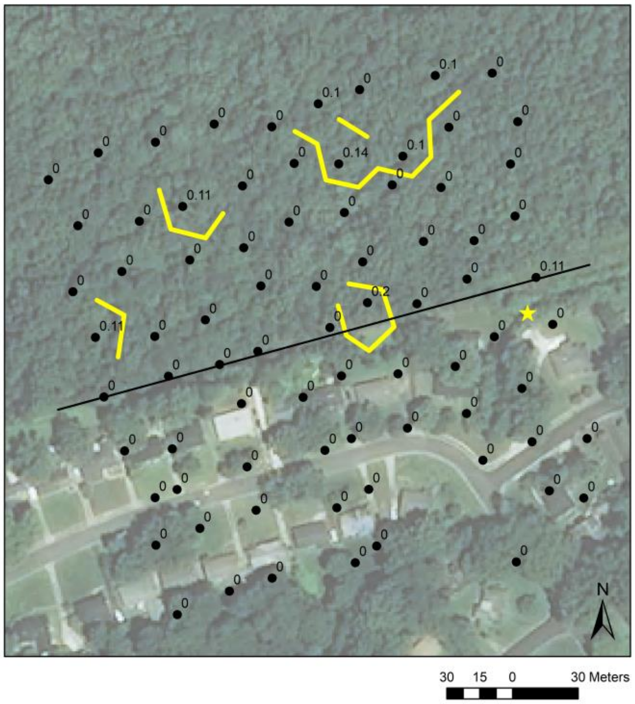

Figure S75. Urban_small_Generalist abundance

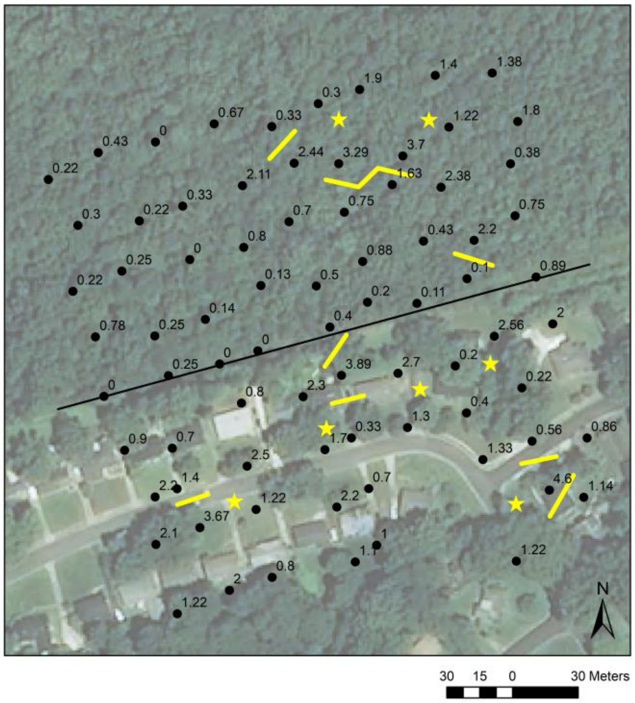

Figure S76. Urban_small_Generalist evenness

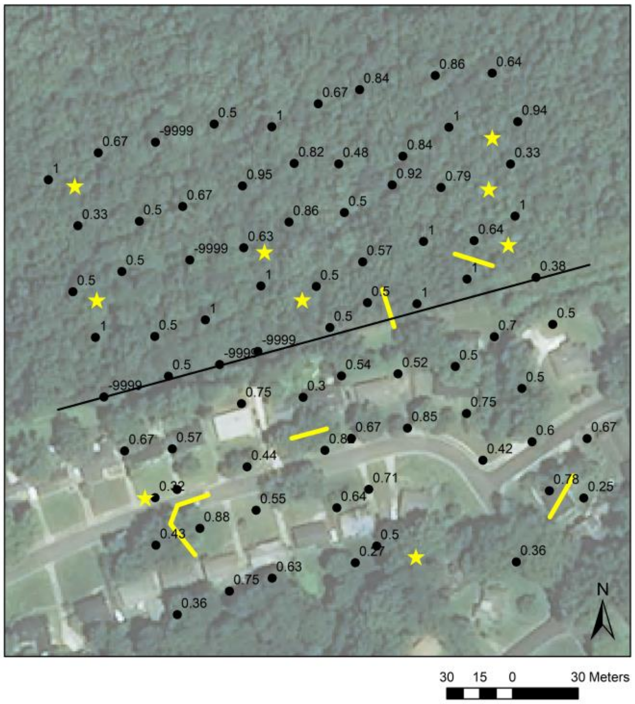

Figure S77. Urban_small_Forest species

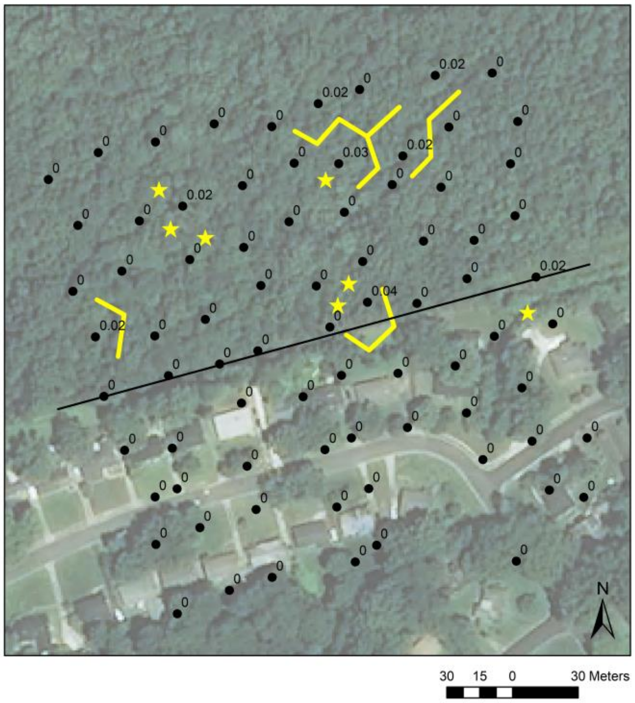

Figure S78. Urban_small_Open species

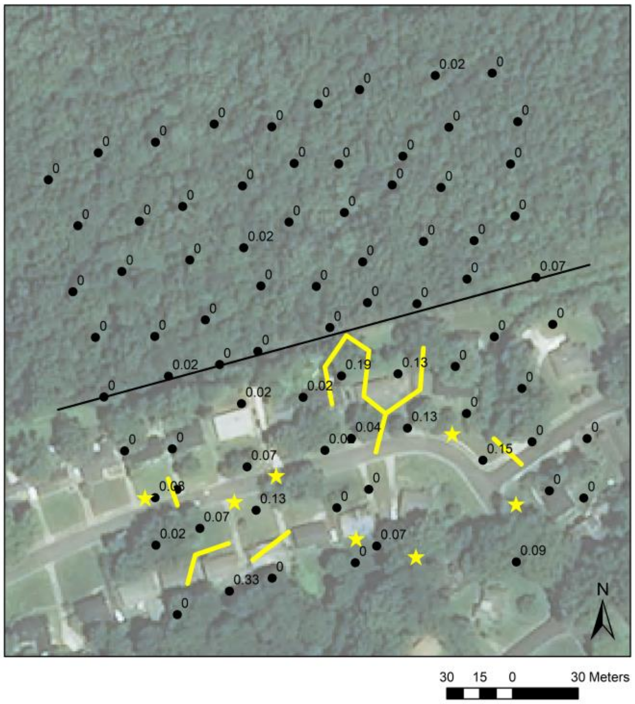

Figure S79. Urban_small_Anisodactylus dulcicollis

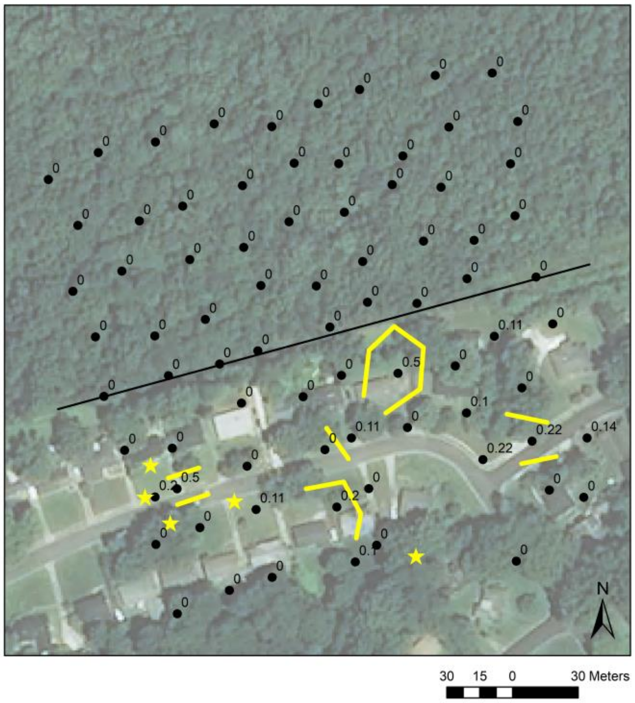

Figure S80. Urban_small_Anisodactylus furvus

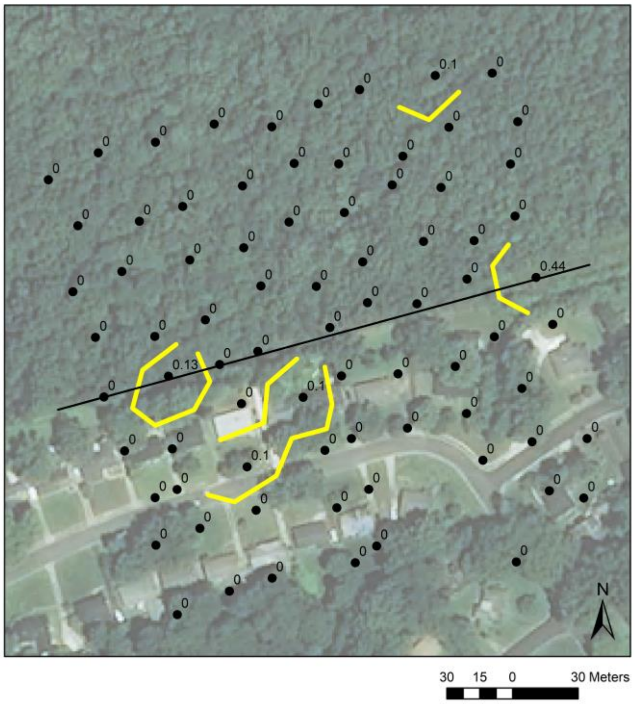

Figure S81. Urban_small_Chlaenius tricolor tricolor

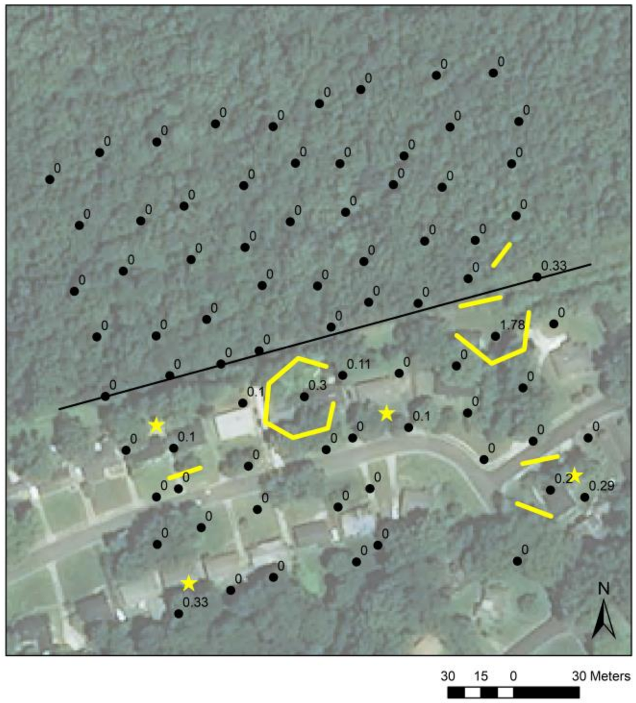

Figure S82. Urban_small_Harpalus longicollis

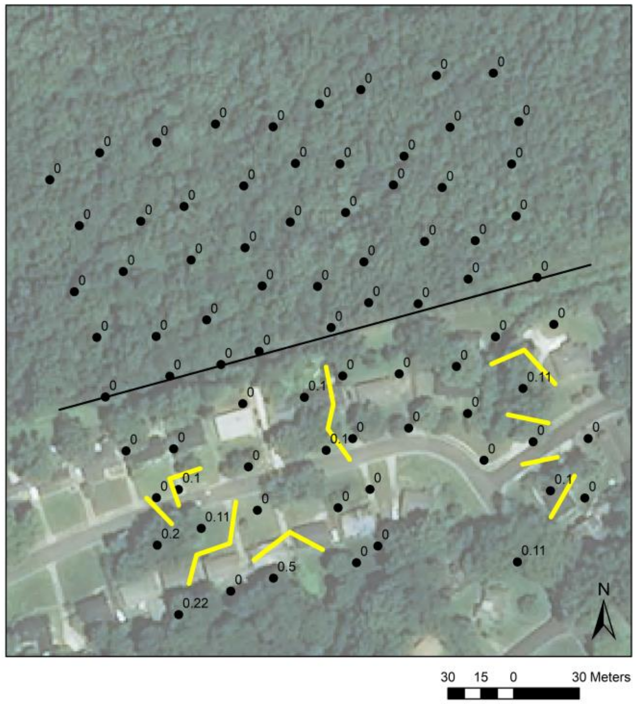

Figure S83. Urban_small_Scarites subterraneus

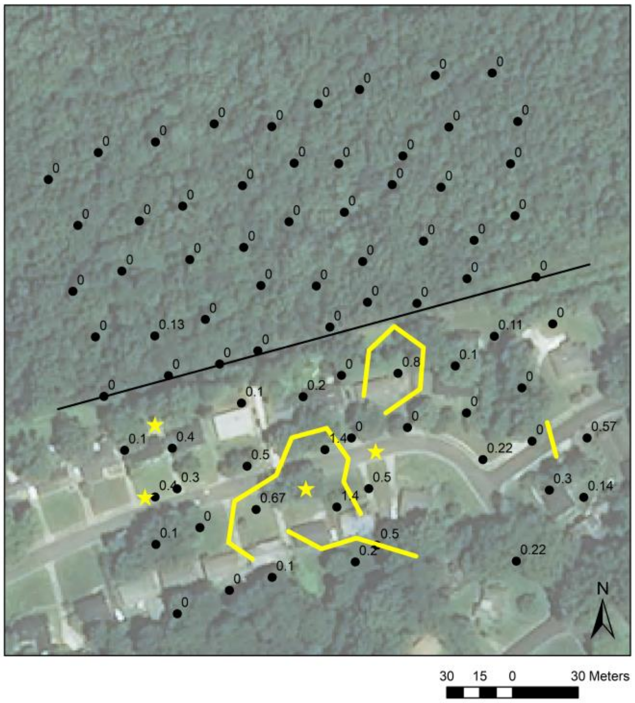

Figure S84. Urban_small_Sphaeroderus stenostomus lecontei

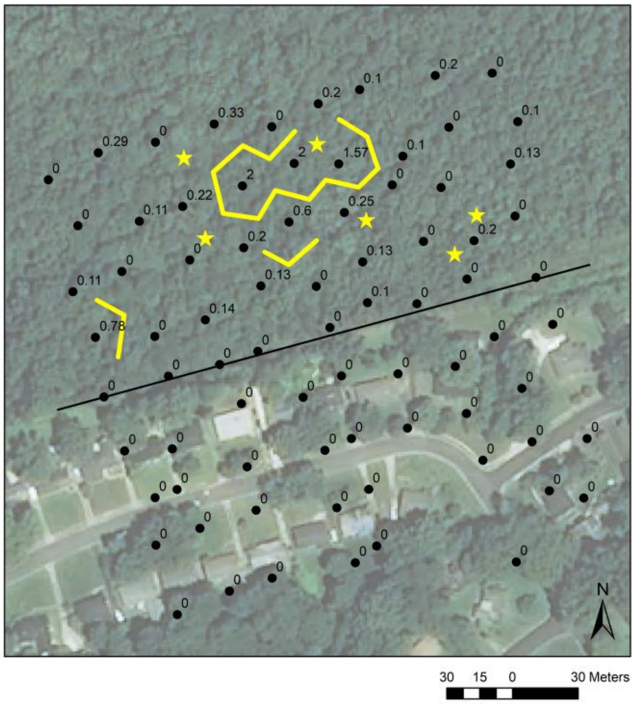

Figure S85. Urban_large_Slope

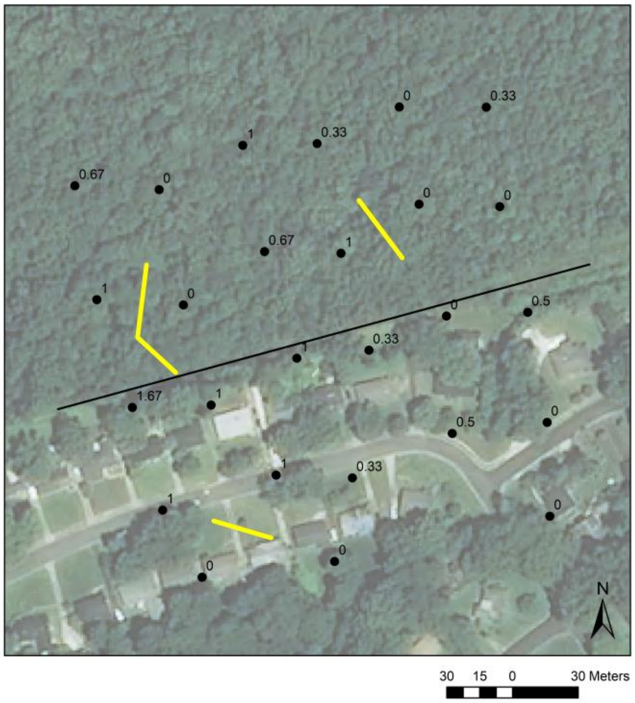

Figure S86. Urban_large_Humidity

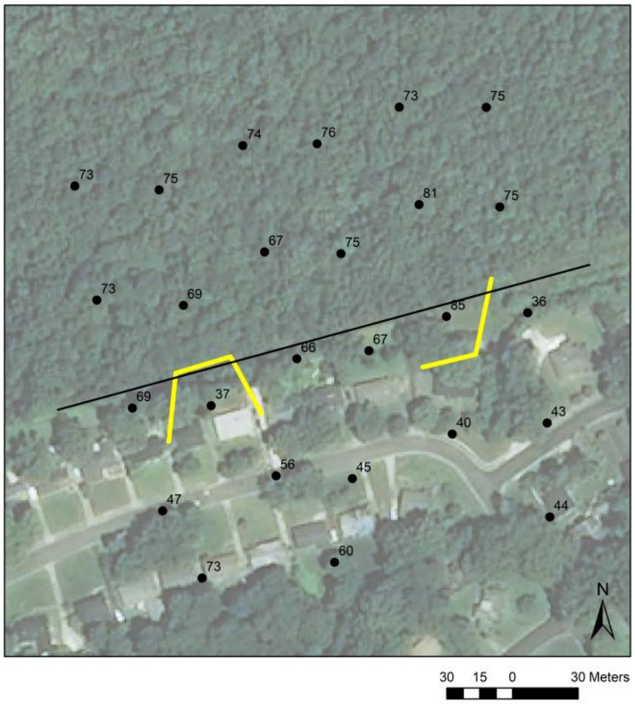

Figure S87. Urban_large_Bare ground cover

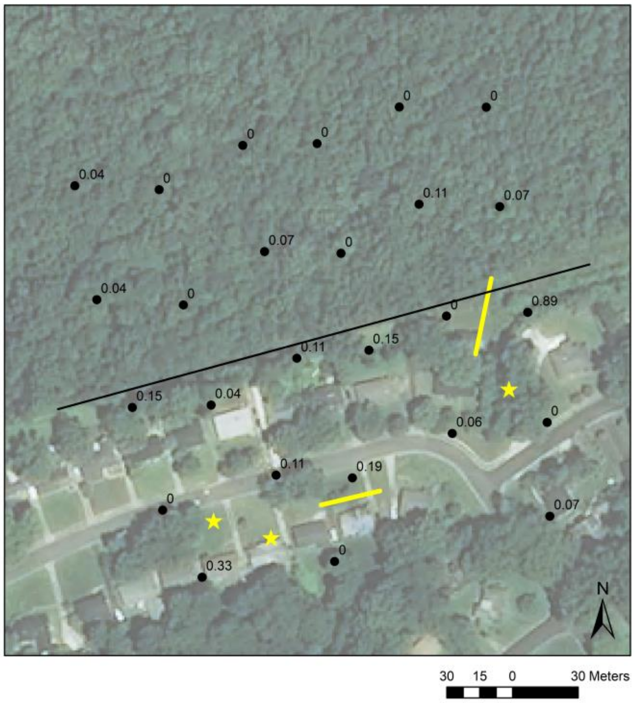

Figure S88. Urban_large_Amara familiaris

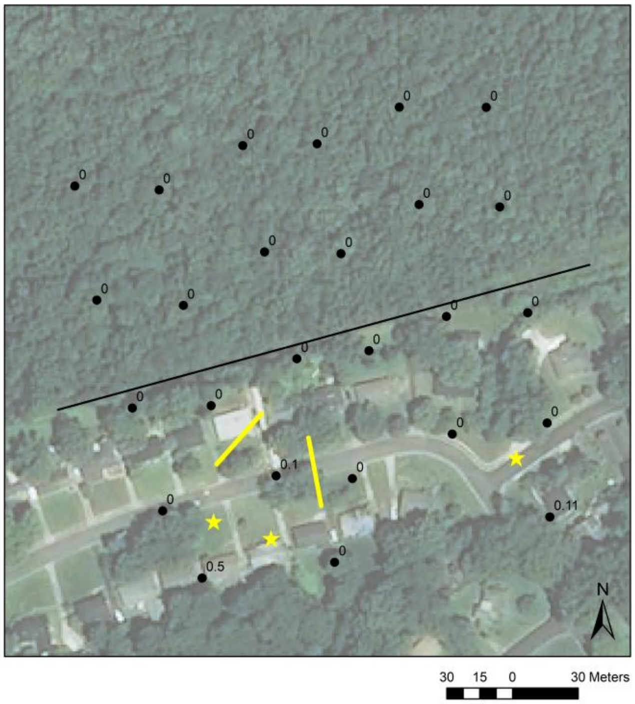

Figure S89. Urban_large_Chlaenius tomentosus

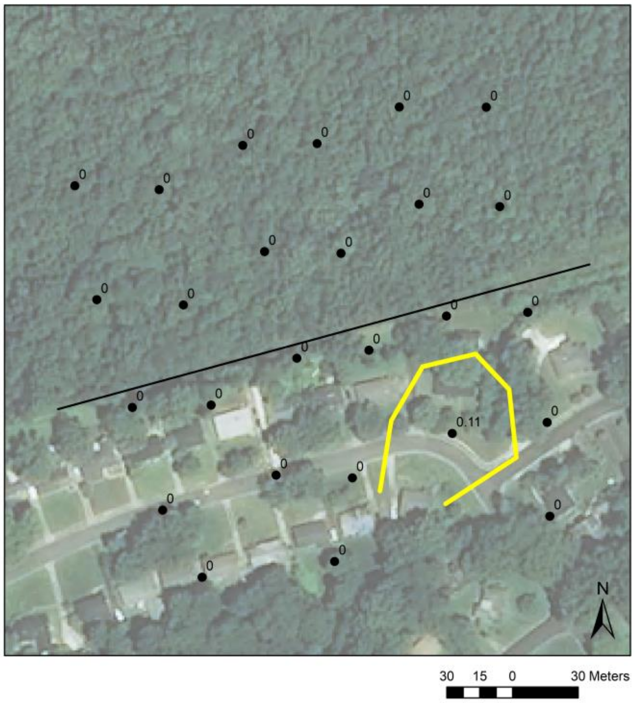

Figure S90. Urban_large_Chlaenius tricolor tricolor

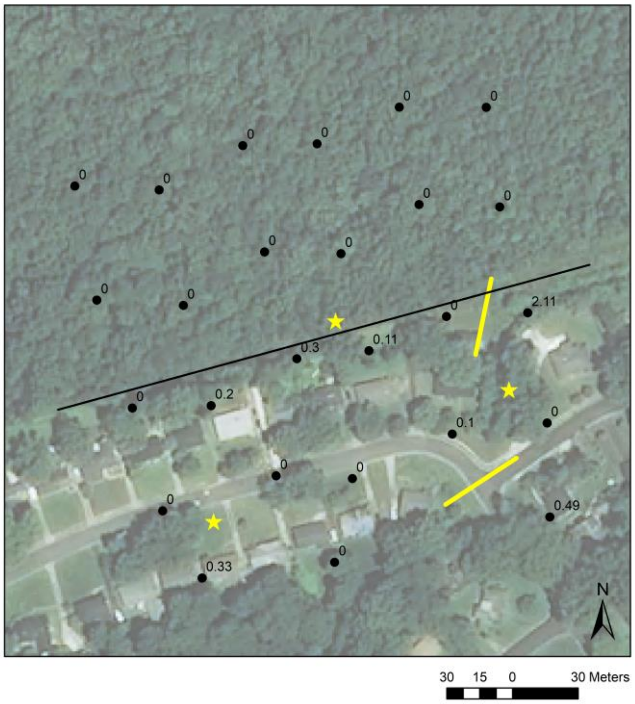

Figure S91. Urban_large_Harpalus pensylvanicus

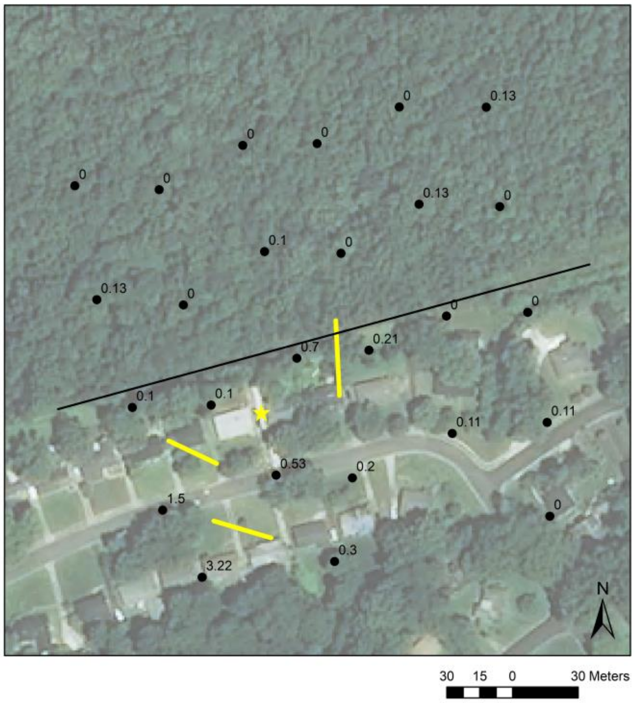

Figure S92. Urban_large_Sphaeroderus stenostomus lecontei

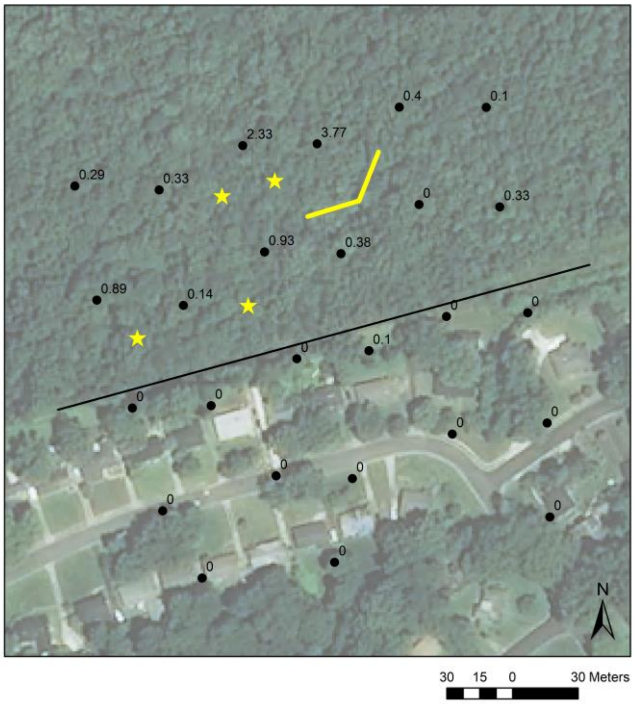

Supplement: Supplemental Information 5 — Boundaries (yellow lines) and singletons (yellow stars) in environmental and ground beetle variables with one or more significant boundary statistics. Figures are named by site (rural, suburban, or urban), spatial scale (small or large), and variable. Black dots are trap locations at the small scale and the centroids of trios of adjacent trap locations at the large scale and are labeled with variable values (temperature in degrees Celsius; humidity as a percentage; microrelief and slope as ordinal indices; leaf litter depth in cm; covers as percentages; all environmental variables as a standardized average of individual variables; abundances and richnesses as numbers of individuals and species, respectively, per trapping period; evennesses as Berger-Parker indices; missing values indicated by −9,999). Edges are indicated by black lines and correspond to the property lines between County-owned forest and private development. Beetle community matrices were analyzed using raw abundances and species abundances weighted by the inverse of the species’ proportion of total abundance at the site. [file peerj-06-4226-s005.pdf]
