# Supplementary material for: Boundaries in ground beetle (Coleoptera: Carabidae) and environmental variables at the edges of forest patches with residential developments
Source: PeerJ. 2018 Jan 8;6:e4226. doi: 10.7717/peerj.4226 (PMC5764035; doi:10.7717/peerj.4226)

Figures S93-S108. Clusters (colored dots), boundaries (yellow lines), and singletons (yellow stars) in environmental and ground beetle variables that exhibited boundaries and singletons at, near, or parallel to edges. Figures are named by site (rural, suburban, or urban), spatial scale (small or large), and variable. Edges are indicated by black lines and correspond to the property lines between County-owned forest and private development.

Figure S93. Rural_small_Forb cover

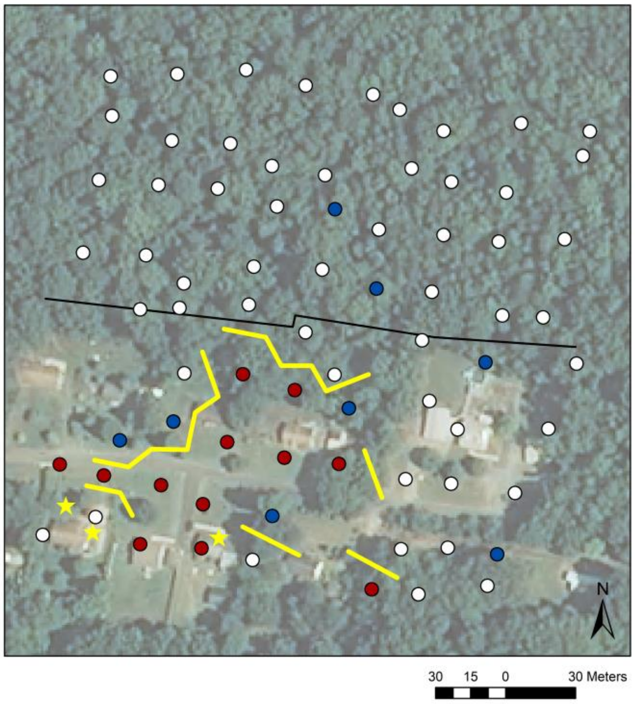

Figure S94. Rural_small_Vine cover

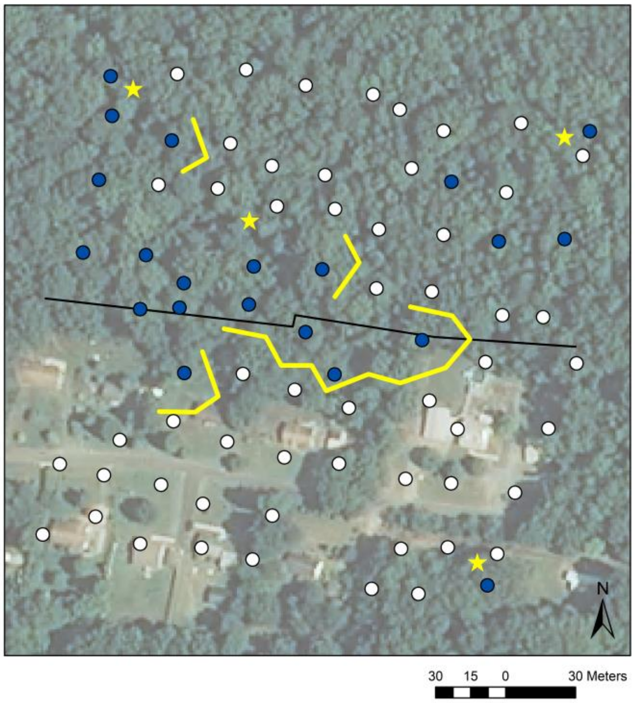

Figure S95. Rural_small_Total evenness

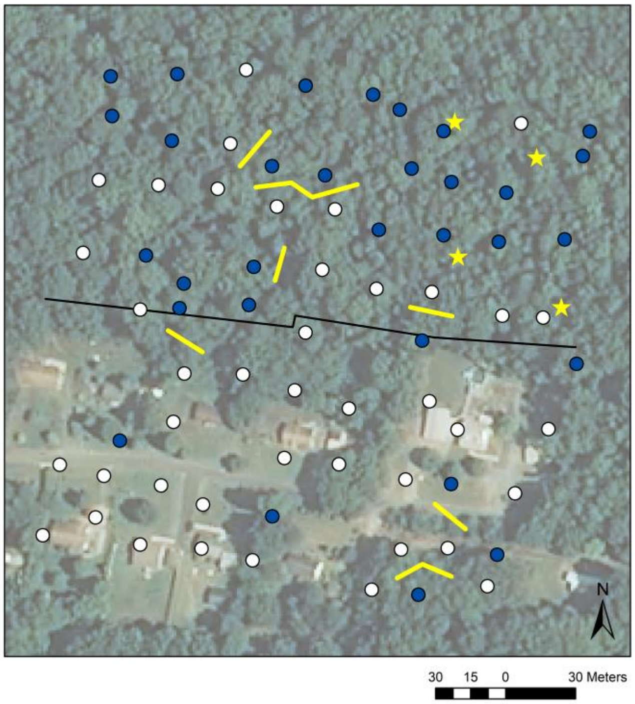

Figure S96. Rural_large_Temperature

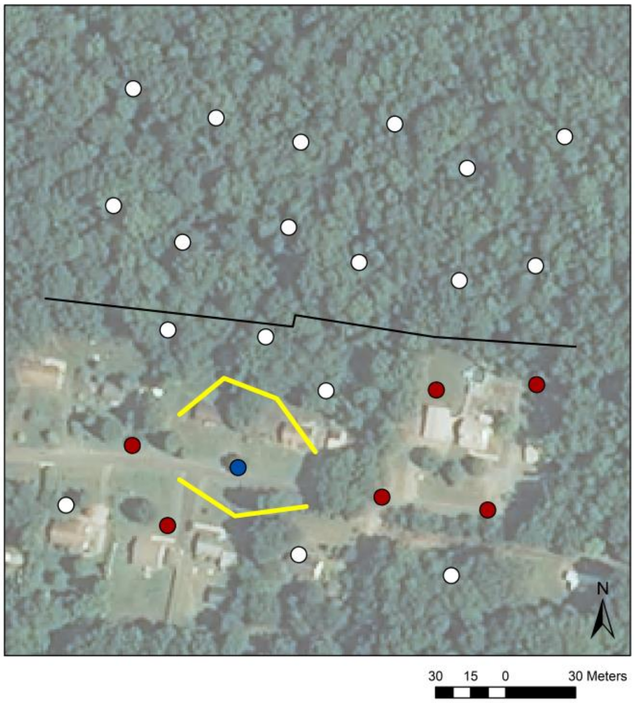

Figure S97. Rural_large_Humidity

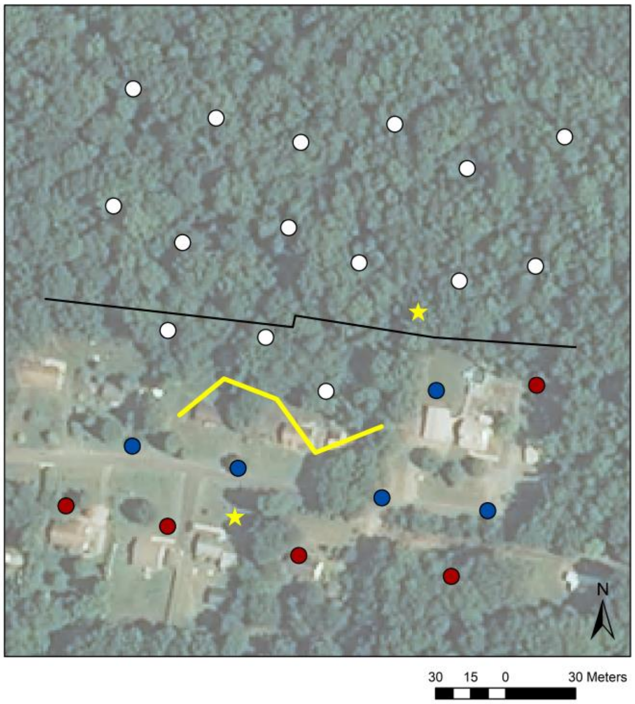

Figure S98. Rural_large_Total evenness

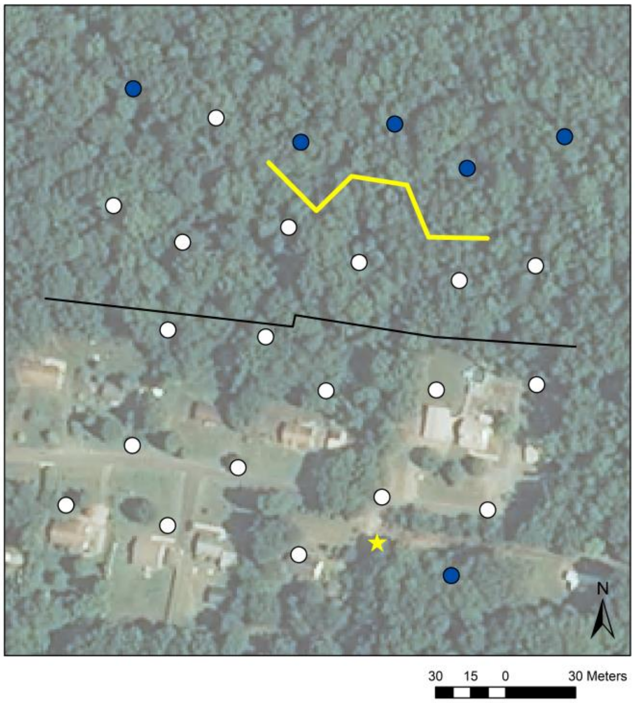

Figure S99. Suburban_small_Leaf litter depth

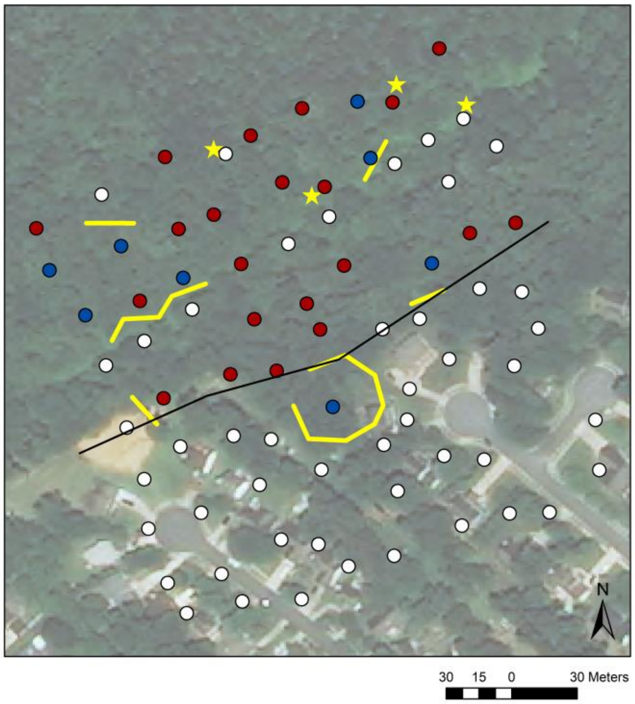

Figure S100. Suburban_small_Shrub cover

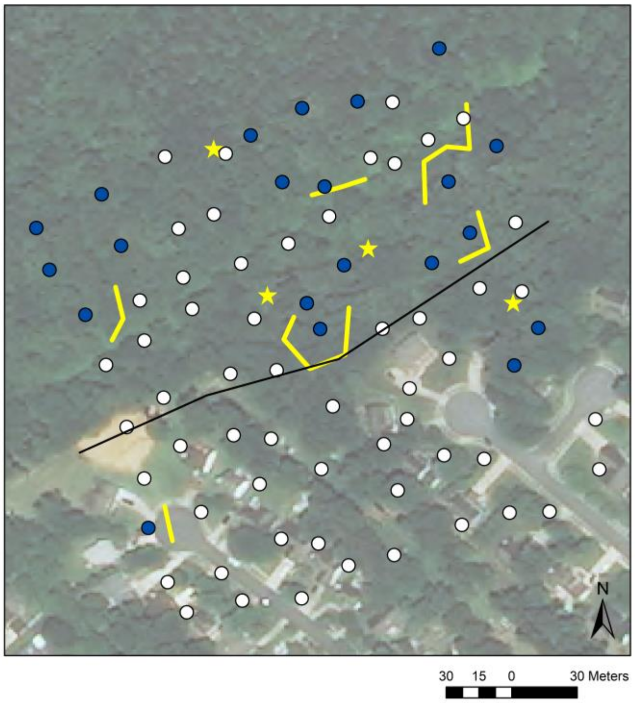

Figure S101. Suburban_small_All environmental variables

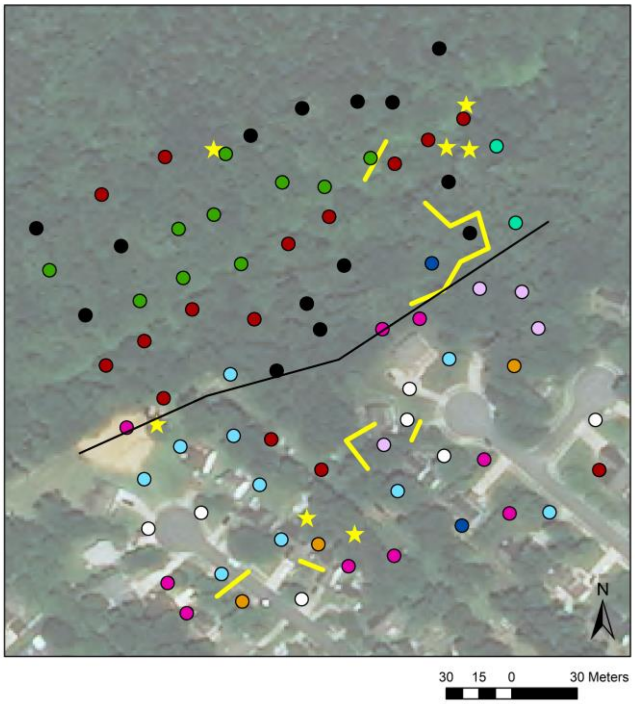

Figure S102. Urban_small_Slope

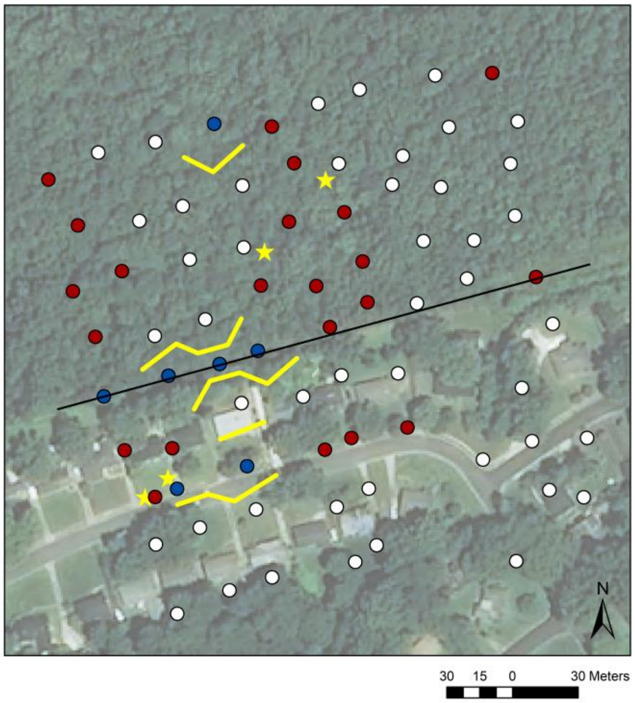

Figure S103. Urban_small_Canopy cover

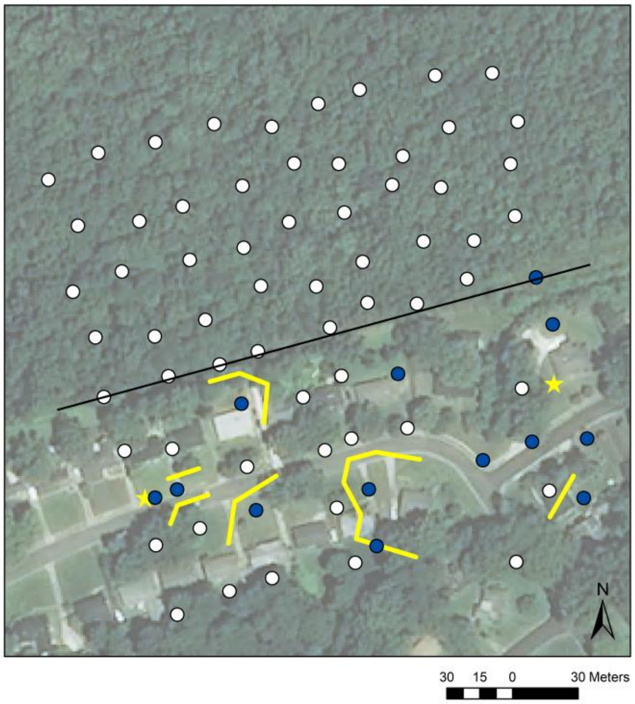

Figure S104. Urban_small_Shrub cover

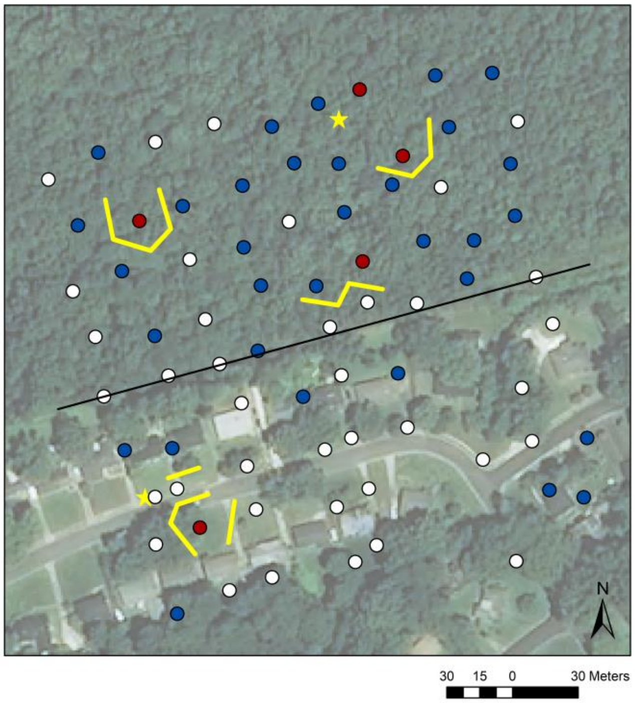

Figure S105. Urban_small_Vine cover

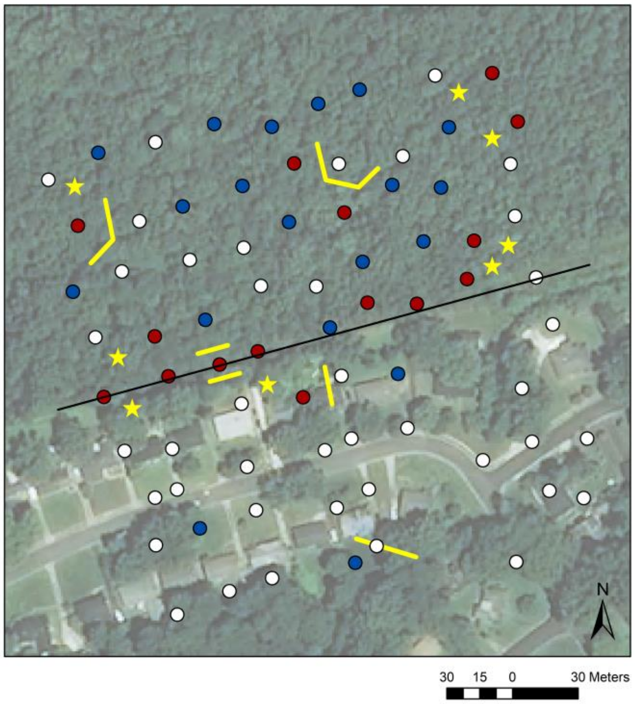

Figure S106. Urban_small_Total evenness

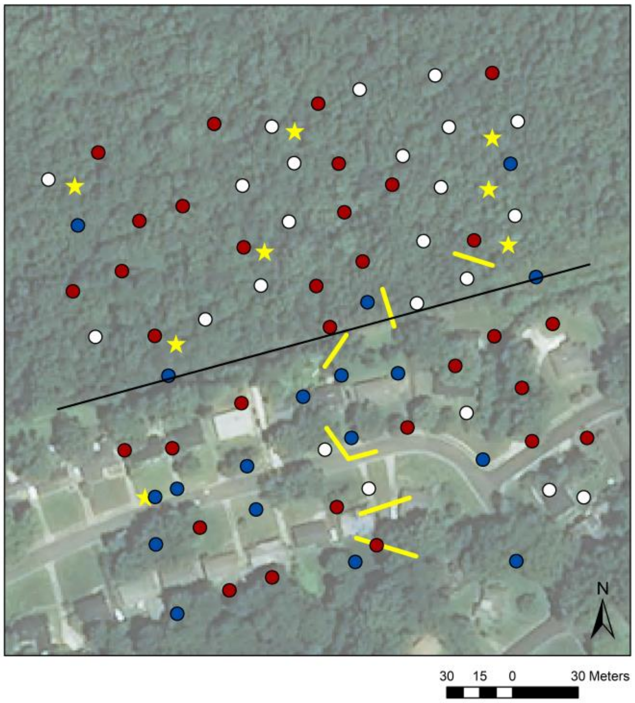

Figure S107. Urban_small_Generalist evenness

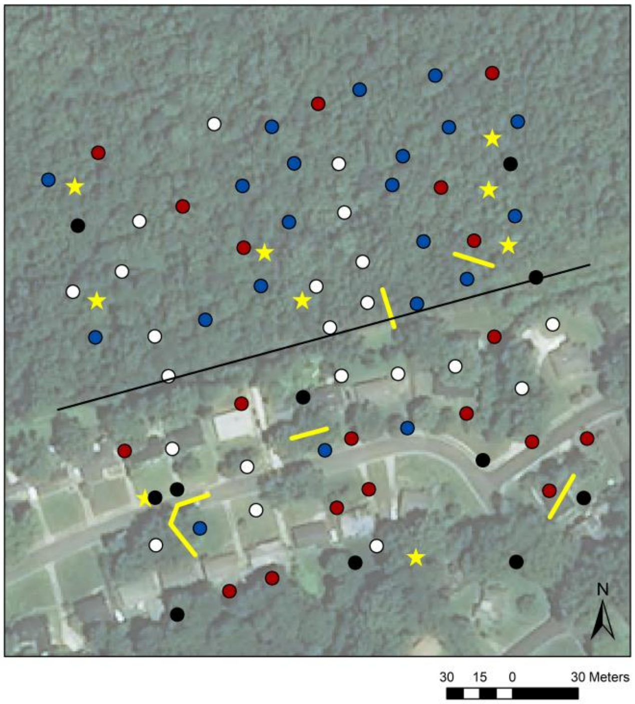

Figure S108. Urban_large_Microrelief

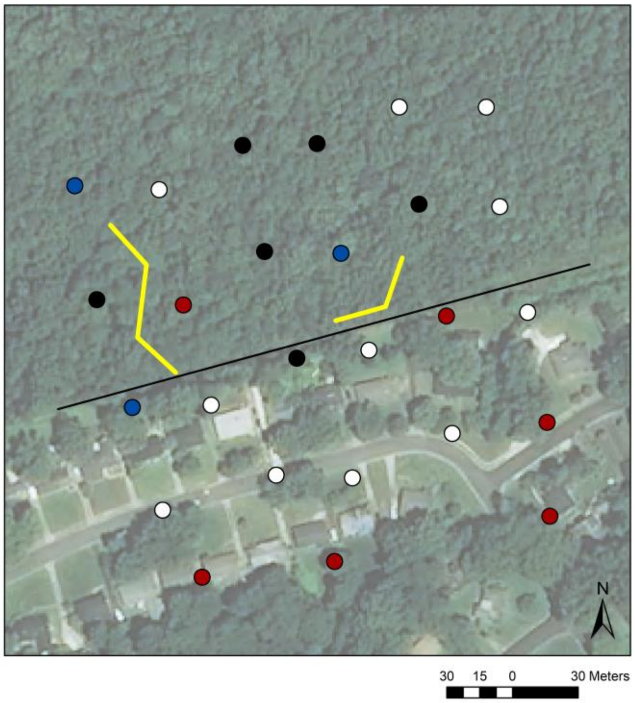

Supplement: Supplemental Information 6 — Clusters (colored dots), boundaries (yellow lines), and singletons (yellow stars) in environmental and ground beetle variables that exhibited boundaries and singletons at, near, or parallel to edges. Figures are named by site (rural, suburban, or urban), spatial scale (small or large), and variable. Edges are indicated by black lines and correspond to the property lines between County-owned forest and private development. [file peerj-06-4226-s006.pdf]
